# Safety and efficacy of a GLP-1 and glucagon receptor dual agonist mazdutide (IBI362) 9 mg and 10 mg in Chinese adults with overweight or obesity: A randomised, placebo-controlled, multiple-ascending-dose phase 1b trial

**DOI:** 10.1016/j.eclinm.2022.101691

**Published:** 2022-10-07

**Authors:** Linong Ji, Leili Gao, Hongwei Jiang, Jing Yang, Lei Yu, Jie Wen, Chenghang Cai, Huan Deng, Liqi Feng, Baili Song, Qingyang Ma, Lei Qian

**Affiliations:** a Department of Endocrinology and Metabolism, Peking University People's Hospital, Beijing, China; b The First Affiliated Hospital and Clinical Medicine College, Henan University of Science and Technology, Luoyang, China; c Department of Endocrinology, The First Hospital of Shanxi Medical University, Taiyuan, China; d Department of Endocrinology, Bengbu Medical College, Bengbu, China; e Innovent Biologics, Inc., Suzhou, China

**Keywords:** Glucagon-like peptide-1, Dual agonist, Overweight, Obesity, Metabolic disorders

## Abstract

**Background:**

Mazdutide (also known as IBI362 or LY3305677), a novel once-weekly glucagon-like peptide-1 (GLP-1) and glucagon receptor dual agonist, achieved 12-week body weight loss up to 6.4% at doses up to 6 mg in Chinese adults with overweight or obesity. We further explored the safety and efficacy of mazdutide dosed up to 9 mg and 10 mg.

**Methods:**

In this randomised, placebo-controlled, multiple-ascending-dose phase 1b trial, we enrolled adults (aged 18-75 years, both inclusive) with overweight (body-mass index [BMI] ≥24 kg/m^2^) accompanied by hyperphagia and/or at least one obesity-related comorbidity or obesity (BMI ≥28 kg/m^2^) from five hospitals in China. Eligible participants were randomly assigned (2:1) within each cohort by using an interactive web-response system to receive once-weekly subcutaneous mazdutide or placebo for 12 weeks in the 9 mg cohort (3 mg weeks 1-4; 6 mg weeks 5-8; 9 mg weeks 9-12) and for 16 weeks in the 10 mg cohort (2.5 mg weeks 1-4; 5 mg weeks 5-8; 7.5 mg weeks 9-12; 10 mg weeks 13-16). The participants, investigators, study site personnel involved in treating and assessing participants in each cohort and sponsor personnel were masked to treatment allocation. The primary outcomes were safety and tolerability of mazdutide, assessed from baseline to end of follow-up in all participants who received at least one dose of the study treatment. The secondary outcomes included the change from baseline to week 12 or week 16 in body weight, waist circumference and BMI. This trial is registered with ClinicalTrials.gov, NCT04440345.

**Findings:**

Between Mar. 1, 2021 and Mar. 26, 2021, a total of 24 participants were enrolled, with eight randomly assigned to mazdutide and four to placebo in each cohort. One participant receiving mazdutide and two receiving placebo in the 10 mg cohort withdrew consent and quitted the study. No serious adverse event was reported. All treatment-emergent adverse events (TEAEs) were mild or moderate in severity and most commonly-reported TEAEs were upper respiratory tract infection, diarrhoea, decreased appetite, nausea, urinary tract infection, abdominal distension and vomiting. The mean percent change from baseline to week 12 in body weight were −11.7% (SE 1.5) for participants receiving mazdutide in the 9 mg cohort and −1.8% (1.6) for participants receiving placebo (estimated treatment difference [ETD]: −9.8%; 95% confidence interval [CI]: −14.4, −5.3; *P* = 0.0002). The mean percent change from baseline to week 16 in body weight were −9.5% (SE 1.7) for participants receiving mazdutide in the 10 mg cohort and −3.3% (1.9) for participants receiving placebo (ETD: −6.2%; 95% CI: −11.5, −0.9; *P* = 0.024). In addition, compared with placebo, mazdutide achieved more profound reductions in waist circumference and BMI.

**Interpretation:**

Mazdutide dosed up to 9 mg and 10 mg was both well tolerated and showed a favourable safety profile. High-dose mazdutide showed promising 12-week body weight loss, holding great potential for the treatment of moderate-to-severe obesity. A larger and longer phase 2 trial will further evaluate the efficacy and safety of high-dose mazdutide in Chinese adults with obesity.

**Funding:**

Innovent Biologics, Inc.

**Research in context**

**Evidence before this study**

A PubMed search on July 4, 2022, with the terms “glucagon-like peptide-1″, [AND] “glucagon receptor”, [AND] “dual agonist”, in the Title or Abstract yielded 31 results. Several GLP-1 and glucagon receptor dual agonists, including cotadutide, SAR425899, JNJ-64565111 and mazdutide, showed overall favourable safety profiles and varying body weight loss efficacy in individuals with overweight or obesity. Mazdutide dosed up to 6 mg achieved 12-week mean body weight loss up to 6.4% in Chinese adults with overweight or obesity.

**Added value of this study**

Mazdutide dosed up to 9 mg and 10 mg was both well tolerated and showed a favourable safety profile similar to that observed with low-dose mazdutide. The estimated treatment difference versus placebo in mean body weight change from baseline was −6.2% with mazdutide 10 mg at week 16 and −9.8% with mazdutide 9 mg at week 12.

**Implication of all the available evidence**

GLP-1 and glucagon dual agonists were promising body weight loss medications but have yet to demonstrate superior body weight loss efficacy to GLP-1 receptor mono-agonists. High-dose mazdutide demonstrated robust 12-week body weight loss, holding great potential for the treatment of moderate-to-severe obesity and related metabolic disorders. A phase 2 trial will further evaluate the efficacy and safety of mazdutide 9 mg in Chinese adults with obesity.

Alt-text: Unlabelled box

## Introduction

Obesity has become a public health threat that accounts for a large proportion of premature mortality and non-communicable diseases.^1^ The prevalence of obesity has increased rapidly in the past several decades in China, where few pharmacotherapy was available despite an overall unsatisfactory body weight control by lifestyle intervention alone.^1^^,^^2^

Glucagon-like peptide-1 (GLP-1) receptor agonists emerged as an efficacious and safe treatment option for obesity.^3^ The rationale for combining agonism at multiple gastrointestinal hormone receptors came from the observed changes in gut hormonal milieu accompanying with substantial metabolic improvements following bariatric surgery.^4^ Oxyntomodulin, a natural proglucagon fragment that stimulates both GLP-1 and glucagon receptors, can simultaneously reduce glucose concentration and body weight when administered pharmacologically.^5^ Given the evidence that glucagon receptor agonism increased energy expenditure,^6^ several GLP-1 and glucagon dual agonists were under development but have yet to demonstrate superior body weight loss efficacy to GLP-1 receptor agonism alone.^3^^,^^7^, ^8^, ^9^

Mazdutide (also known as IBI362 or LY3305677) is a once-weekly mammalian oxyntomodulin analogue. Mazdutide potently bound to human and mouse GLP-1 receptors and glucagon receptors *in vitro*. In mice, mazdutide improved glucose control, decreased body weight in both *Gcgr* knockout (KO) and *Glp1r* KO settings and increased energy expenditure.^10^ In a phase 1b clinical trial in Chinese adults with overweight or obesity, mazdutide dosed up to 6 mg achieved 12-week mean body weight loss up to 6.4%.^11^ Given the favourable safety profile and the robust body weight loss achieved in the low-dose cohorts, we further assessed the safety and efficacy of mazdutide dose up to 9 mg and 10 mg in Chinese adults with overweight or obesity in two additional cohorts.

## Methods

### Study design

This randomised, placebo-controlled, multiple-ascending-dose study was designed to evaluate the safety, tolerability, pharmacokinetics and efficacy of mazdutide in Chinese adults with overweight or obesity. The 9 mg and 10 mg cohorts were added to the study after the completion of the previous cohorts and enrolled participants from five hospitals in China. The study was done in accordance with local laws, the International Conference on harmonization Good Clinical Practice guidelines, and the ethical principles outlined in the Declaration of Helsinki. Ethics committees of participating study centres reviewed the study protocol and approved the study. The results of the study were reported in adherence to the CONSORT reporting guidelines.

### Participants

Adults (aged 18-75 years, both inclusive) with overweight (body-mass index [BMI] ≥24 kg/m^2^) accompanied by hyperphagia and/or at least one obesity-related comorbidity (pre-diabetes, hypertension, dyslipidaemia, fatty liver, weight-bearing arthralgia or dyspnoea, obstructive sleep apnoea syndrome) or obesity (BMI ≥28 kg/m^2^) and with less than 5% body weight loss by diet and exercise 12 weeks or longer prior to screening were eligible for this study. The key exclusion criteria were previous use of GLP-1 receptor agonists, use of body weight loss or anti-obesity agents three months prior to screening. All participants provided written informed consent before study entry.

### Randomisation and masking

An interactive web-response system generated identification numbers that were used to randomly assign eligible participants 2:1 to receive mazdutide or placebo in each cohort. Randomisation list was generated by an in-house statistician who was not involved in the clinical operations of the study. The study drugs and placebo were identically labelled and indistinguishable in appearance. As such, the participants, investigators, study site personnel involved in treating and assessing participants in each cohort and sponsor personnel were masked to treatment allocation.

### Procedures

The study included a 3-week screening period, a 12-week or 16-week treatment period and an 8-week safety follow-up period. During the treatment period, mazdutide or placebo was subcutaneously administered once weekly with one of the following dose-escalation regimens: 9 mg cohort (3 mg weeks 1-4; 6 mg weeks 5-8; 9 mg weeks 9-12) and 10 mg cohort (2.5 mg weeks 1-4; 5 mg weeks 5-8; 7.5 mg weeks 9-12; 10 mg weeks 13-16) (Figure S1). The escalation regimen in the 10 mg cohort provided relatively slower titration (2.5 mg every 4 weeks) to the target dose of 10 mg, and the escalation regimen in the 9 mg cohort adopted faster titration (3 mg every 4 week). Participants were required to maintain the original diet, exercise and lifestyle during the trial. Investigators and authorized study personnel in each centre recorded the study data into the electronic case report forms (eCRFs), which were reviewed and validated by clinical research associates. The data in the eCRFs were submitted to the electronic data capture database. HbA_1c_ tests were performed in a central laboratory (Wuxi AppTec Inc., *Shanghai*).

### Outcomes

The primary outcomes were safety and tolerability of mazdutide. Treatment-emergent adverse events (TEAEs) were monitored from baseline to the end of safety follow-up and were categorized according to the Medical Dictionary for Regulatory Activities system organ class and preferred terms. The severity of the TEAEs (mild, moderate or severe) and the association between an event and the study drug was assessed by investigators based on pre-specified criteria.

Secondary outcomes included pharmacokinetics, assessed by maximum observed plasma concentration (C_max_), time at which C_max_ was observed (T_max_), terminal elimination half-life (t_½_), and area under the curve from time zero to 168 h after the first dose (AUC_0–168 h_) and immunogenicity, assessed by titters of anti-drug antibodies (ADAs) and neutralizing antibodies. Secondary efficacy outcomes included the change from baseline to week 12 (9 mg cohort) or week 16 (10 mg cohort) in body weight, waist circumference, BMI, as well as the change from baseline in HbA_1c_, lipids, transaminase and serum uric acid levels.

### Statistical analysis

The sample size of 12 participants per cohort was empirically determined to provide preliminary safety, tolerability, pharmacokinetics data while minimizing the number of participants. No formal hypothesis testing was planned. Participants receiving placebo were pooled for analysis on the change from baseline in body weight, waist circumference and BMI.

Safety analyses were done in the safety population (defined as all participants who received at least one dose of the study treatment). Pharmacokinetic analyses were done in the pharmacokinetic population (defined as all participants who received at least one dose of mazdutide and had at least one valid plasma concentration assessment). Efficacy analyses were done in the efficacy population (defined as all participants who received at least one dose of the study treatment and had at least one post-baseline assessment). Immunogenicity analyses were done in the immunogenicity population (defined as all patients who received at least one dose of the study treatment and had at least one post-baseline ADA result).

The analyses for the change from baseline in body weight, waist circumference and BMI were performed using mixed-effect model for repeated measures (MMRM), with least squares mean change or mean percent change from baseline at each follow-up time analysed using a restricted maximum likelihood (REML)‐based repeated measures approach in combination with the Newton Raphson Algorithm. The MMRM included the corresponding baseline value, treatment, visit and treatment-by-visit as fixed effects and patient as a random effect, with an unstructured covariance matrix used. In case of non-converge, matrix like Variance Components and Compound Symmetry will be tested in a subsequent order until model‐convergence is achieved. The Kenward-Roger degree of freedom was used in estimating fixed effects. No missing data imputation was conducted. Point estimate of treatment difference versus placebo at week 12 for the 9 mg cohort and at week 16 for the 10 mg cohort were provided, together with corresponding 95% CI and nominal p value.

Summaries for other efficacy and safety variables included descriptive statistics for continuous measures (mean and standard deviation or standard error) and for categorical measures (sample size, frequency and percentages).

All statistical analyses were done using SAS version 9.4. This study is registered with ClinicalTrials.gov, number NCT04440345.

### Role of the funding source

The funder of the study was involved in the study design, data collection, data review, data analysis, data interpretation and writing of the report. All authors verified that this study was done according to the protocol and was attested for data accuracy and completeness. All authors had full access to all the data in the study, contributed to writing and review of the report, and approved the final submitted version. The corresponding authors had final responsibility for the decision to submit for publication.

## Results

### Participants

**Figure 1:**
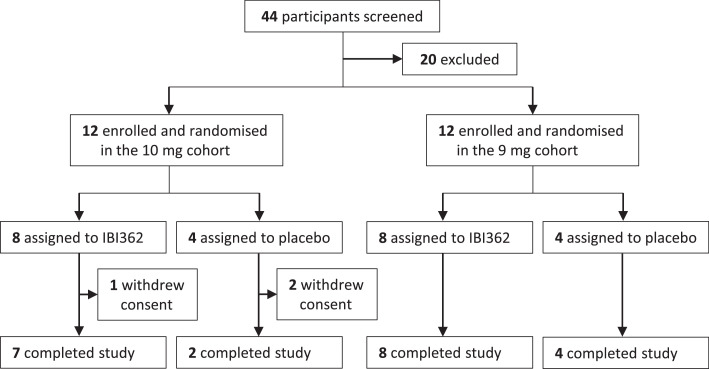
**Trial profile**.

A total of 44 participants were screened for eligibility, of whom 24 were enrolled. In each cohort, 12 participants were randomised, with eight assigned to mazdutide and four assigned to placebo. One participant receiving mazdutide and two receiving placebo in the 10 mg cohort withdrew consent and quitted the study. The remaining 21 participants (87.5%) completed the study (Figure 1). All randomised participants (*n* = 24) were included in the safety, efficacy and immunogenicity population. All participants receiving mazdutide (*n* = 16) were include in the pharmacokinetic population. Baseline body weight, waist circumference and BMI were essentially balanced between treatment groups (Table 1).

**Table 1:** Demographics and baseline characteristics.

	10 mg cohort		9 mg cohort		Pooled placebo (*n* = 8)
	Mazdutide (*n* = 8)	Placebo (*n* = 4)	Mazdutide (*n* = 8)	Placebo (*n* = 4)	
Age (years)	36.0 (9.1)	34.3 (14.0)	37.9 (9.7)	45.5 (6.4)	39.9 (11.7)
Sex					
Female	6 (75.0%)	3 (75.0%)	6 (75.0%)	2 (50.0%)	5 (62.5%)
Male	2 (25.0%)	1 (25.0%)	2 (25.0%)	2 (50.0%)	3 (37.5%)
BMI (kg/m^2^)	31.8 (5.1)	29.1 (4.6)	30.1 (3.8)	30.1 (1.7)	29.6 (3.3)
Body weight (kg)	82.8 (14.1)	77.2 (18.6)	79.8 (15.5)	83.6 (7.3)	80.4 (13.5)
Waist circumference (cm)	101.1 (11.5)	95.0 (11.7)	97.2 (13.7)	105.1 (7.7)	100.0 (10.6)
Systolic blood pressure (mm Hg)	119.4 (8.8)	108 (8.7)	118.3 (10.2)	118.0 (11.1)	113.0 (10.7)
Diastolic blood pressure (mm Hg)	84.8 (7.5)	77.0 (6.1)	80.5 (8.1)	82.0 (8.1)	79.5 (7.1)

Data are presented as n (%) or mean (standard deviation). BMI = body-mass index.

### Safety

Mazdutide was well tolerated and showed a favourable safety profile (Table 2 and S1). No participant discontinued the study due to adverse events. No dose adjustment of the study drug was made. No serious adverse event was reported. Treatment-emergent adverse events were reported in all participants (100%) receiving mazdutide and seven (87.5%) receiving placebo, all mild or moderate in severity. The most commonly-reported TEAEs were upper respiratory tract infection, diarrhoea, decreased appetite, nausea, urinary tract infection, abdominal distension and vomiting. The incidence of gastrointestinal adverse events (diarrhoea, nausea and vomiting) was higher in participants receiving mazdutide in the 9 mg cohort and declined over time in both cohorts during the up-titration (Figure S2).

**Table 2:** Treatment-emergent adverse events.

	10 mg cohort		9 mg cohort		Pooled placebo (*n* = 8)
	Mazdutide (*n* = 8)	Placebo (*n* = 4)	Mazdutide (*n* = 8)	Placebo (*n* = 4)	
**Any TEAE**	8 (100)	4 (100)	8 (100)	3 (75)	7 (87.5)
**Serious TEAE**	0	0	0	0	0
**TEAE leading to study discontinuation**	0	0	0	0	0
**Most commonly-reported TEAE**^a^					
Upper respiratory tract infection	4 (50)	2 (50)	3 (37.5)	1 (25)	3 (37.5)
Diarrhoea	4 (50)	2 (50)	2 (25)	2 (50)	4 (50)
Decreased appetite	1 (12.5)	1 (25)	5 (62.5)	1 (25)	2 (25)
Nausea	1 (12.5)	1 (25)	5 (62.5)	0	1 (12.5)
Urinary tract infection	2 (25)	0	4 (50)	1 (25)	1 (12.5)
Abdominal distension	0	2 (50)	4 (50)	1 (25)	3 (37.5)
Vomiting	3 (37.5)	0	3 (37.5)	0	0
**Other TEAE of clinical interest**^a^					
Alanine aminotransferase increased	1 (12.5)	0	1 (12.5)	0	0
Aspartate aminotransferase increased	1 (12.5)	0	1 (12.5)	0	0
Lipase increased	1 (12.5)	0	0	1 (25)	1 (12.5)
Ventricular extrasystoles	0	1 (25)	1 (12.5)	0	1 (12.5)

Data are presented as n (%).

a By the Medical Dictionary for Regulatory Activities (MedDRA, version 24.0) preferred term. TEAE = treatment-emergent adverse event.

Slight increase in lipase level were observed in participants receiving mazdutide in both cohorts. One participant receiving mazdutide in the 10 mg cohort had lipase increased from 153 U/L at baseline to 739 U/L at week 16 (upper limit of normal is 300 U/L), and returned to normal at the safety follow-up visit. The participant had adverse event of lipase increased. Amylase levels were below the upper limit of normal for all participants throughout the study. No investigator-suspected pancreatitis was reported. Calcitonin levels remained within normal reference range for all participants and there was no report of thyroid tumours, neoplasms or C-cell hyperplasia events.

One participant receiving mazdutide in the 9 mg cohort and one receiving mazdutide in the 10 mg cohort reported adverse events of alanine aminotransferase increased and aspartate aminotransferase increased. Transaminase elevation in both participants were mild (within three times upper limit of normal) and transient.

Adverse events of ventricular extrasystoles were reported in one participant receiving mazdutide in the 9 mg cohort and one receiving placebo, both mild in severity. Heart rate increase was evident in participants receiving mazdutide in both cohorts. The mean change from baseline in heart rate were up to 17.4 beats per minute for participants receiving mazdutide in the 9 mg cohort and 11.6 beats per minute for those in the 10 mg cohort, compared with 4.3 beats per minute for those receiving placebo (Figure S3). Meanwhile, decrease in systolic blood pressure (mean change from baseline up to −12 mm Hg) was observed in participants receiving mazdutide in both cohorts. Similar trend was observed in diastolic blood pressure (Figure S3).

Two participants receiving mazdutide in the 9 mg cohort and four receiving mazdutide in the 10 mg cohort developed treatment-induced anti-mazdutide antibodies. One participant receiving mazdutide in the 10 mg cohort developed neutralizing antibodies that attenuated cAMP generation in CHO cells over-expressing human recombinant glucagon receptors.

### Pharmacokinetics

Mazdutide demonstrated slow absorption with peak concentrations (C_max_) achieved ranging from 12.1 hours to 170.2 hours, and the median T_max_ is approximately 72 hours. After C_max_ was reached, mazdutide concentrations declined slowly over several weeks with half-life (t_1/2_) ranging from 174.8 hours to 1075.7 hours (7.3 days to 44.8 days) (Table 3).

**Table 3:** Non-compartmental pharmacokinetic parameters following the first dose.

	Mazdutide 2.5 mg (*n* = 8)	Mazdutide 3 mg (*n* = 8)
C_max_ (ng/mL)	179 (25.3)	226.1 (20.5)
T_max_ (h)	72.3 (12.1–119.3)	72.4 (47.9–170.2)
AUC_0-168h_ (ng·h/mL)	21816.4 (34)	31359.8 (24.9)

Data are geometric mean (CV%) for C_max_ and AUC, median (range) for T_max_. AUC_0-168h_ = area under the concentration versus time curve from time zero to 168 hours; C_max_ =maximum observed drug concentration; CV% = coefficient of variation; T_max_ = time of C_max_.

### Efficacy

**Figure 2:**
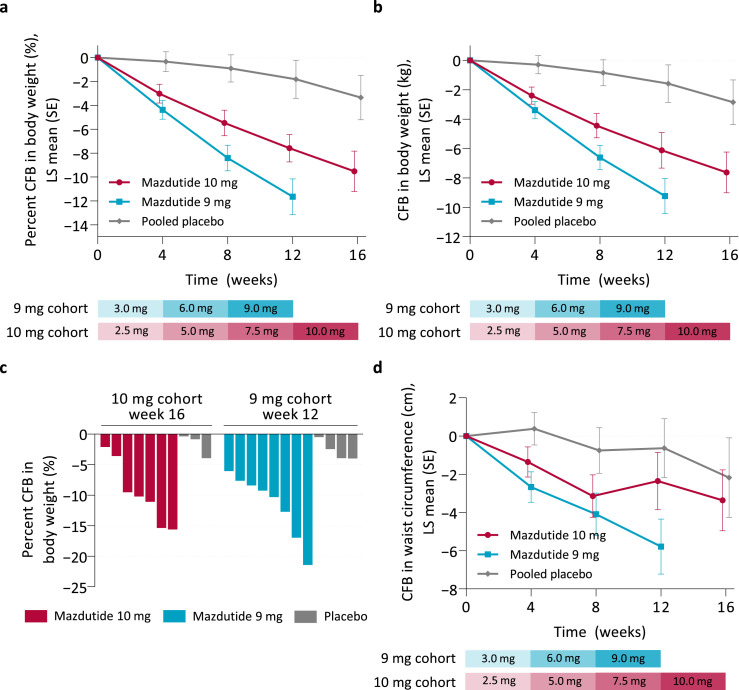
**Change from baseline in body weight and waist circumference**. **a.** Percent change from baseline in bodyweight over time. **b.** Change from baseline in bodyweight over time. **c.** Percent change from baseline at week 12 (9 mg cohort) or week 16 (10 mg cohort) for each participant. **d.** Change from baseline in waist circumference over time. Data in **a, b, d** are plotted as LS means +/− SEM from an MMRM model. Mazdutide 10 mg *n* = 8; Mazdutide 9 mg *n* = 8; Pooled placebo *n* = 8. CFB = change from baseline; LS = least squares; SE = standard error of the mean.

Both dose regimens of mazdutide markedly reduced body weight and BMI, with more pronounced reduction achieved with the 9 mg dose regimen (Figure 2a and b, Table 4). The mean percent change from baseline to week 12 in body weight were −11.7% (SE 1.5) for participants receiving mazdutide in the 9 mg cohort and −1.8% (1.6) for participants receiving placebo (estimated treatment difference [ETD]: −9.8%; 95% confidence interval [CI]: −14.4, −5.3; *P* = 0.0002). The mean percent change from baseline to week 16 in body weight were −9.5% (SE 1.7) for participants receiving mazdutide in the 10 mg cohort and −3.3% (1.9) for participants receiving placebo (ETD: −6.2%; 95%CI: −11.5, −0.9; *P* = 0.024). Four participants (50%) receiving mazdutide in each cohort achieved 10% body weight loss or more and two (25%) receiving mazdutide in each cohort achieved 15% body weight loss or more during the study, while no participant receiving placebo achieved 5% body weight loss or more (Figure 2c).

**Table 4:** Key efficacy outcomes.

	Week 16				Week 12			
	Mazdutide 10 mg (*n* = 8)	Placebo (*n* = 4)	ETD	P*	Mazdutide 9 mg (*n* = 8)	Pooled placebo (*n* = 8)	ETD	P*
Percent CFB in body weight (%)	−9.5 (1.7)	−3.3 (1.9)	−6.2 (−11.5, −0.9)	0.024	−11.7 (1.5)	−1.8 (1.6)	−9.8 (−14.4, −5.3)	0.0002
CFB in body weight (kg)	−7.62 (1.39)	−2.85 (1.52)	−4.76 (−9.10, −0.43)	0.033	−9.23 (1.2)	−1.58 (1.28)	−7.65 (−11.32, −3.98)	0.0003
CFB in BMI (kg/m^2^)	−2.96 (0.56)	−1.07 (0.61)	−1.89 (−3.63, −0.15)	0.035	−3.51 (0.46)	−0.55 (0.50)	−2.96 (−4.37, −1.54)	0.0003
CFB in waist circumference (cm)	−3.36 (1.59)	−2.18 (2.08)	−1.18 (−7.65, 5.29)	0.67	−5.79 (1.45)	−0.64 (1.55)	−5.15 (−9.63, −0.67)	0.027

Data are presented as least square means with standard errors or 95% confidence intervals.

⁎ All *P* values are based on two-sided t-test from the MMRM model and not adjusted for multiple comparisons. BMI = body-mass index; CFB = change from baseline; ETD = estimated treatment difference.

In accordance with the decrease in body weight, mazdutide 9 mg significantly reduced waist circumference (Figure 2d and Table 4). The mean change from baseline to week 12 in waist circumference were −5.79 cm (SE 1.45) with mazdutide and −0.64 cm (1.55) with placebo (ETD: −5.15 cm; 95%CI: −9.63, −0.67; *P* = 0.027). Waist circumference reduction was less pronounced with mazdutide 10 mg and did not reach statistical significance compared with placebo (Table 4).

Notably, in participants without type 2 diabetes, mazdutide showed promising effects on HbA_1c_ reduction (Figure S4). A 0.4% HbA_1c_ reduction from baseline was achieved in two participants receiving mazdutide in the 10 mg cohort at week 16 and three participants receiving mazdutide in the 9 mg cohort at week 12. Furthermore, a 0.8% HbA_1c_ reduction from baseline was achieved in two participants receiving mazdutide in the 9 mg cohort at week 12. One participant receiving mazdutide in the 9 mg cohort and one receiving placebo reported adverse event of hypoglycaemia, both mild in severity.

Greater improvements in multiple metabolic parameters including triglycerides, low density lipoprotein cholesterol, total cholesterol and serum uric acid were observed with both dose regimens of mazdutide, as compared with placebo (Figure S5 and S6).

## Discussion

Taken together, the high-dose cohorts of the randomised, placebo-controlled phase 1b study demonstrated the safety and preliminary efficacy of mazdutide 9 mg and 10 mg in Chinese adults with overweight or obesity. These data support future development of high-dose mazdutide as a promising GLP-1 and glucagon-based anti-obesity drug.

Consistent with the safety profile in low-dose cohorts of the study, gastrointestinal adverse events were most commonly reported.^11^ In low-dose cohorts, we observed that gastrointestinal adverse events were more common with higher initiation doses and faster up-titration regimens (4.5 and 6 mg cohorts).^11^ In high-dose cohorts, increase in the incidence of gastrointestinal adverse events were observed, most notably in nausea (up to 62.5% in high dose cohorts vs. up to 37.5% in low dose cohorts), abdominal distension (50.0% vs 12.5%), diarrhoea (50.0% vs. 37.5%) and vomiting (37.5% vs. 12.5%). Of note, although numerically higher incidence of gastrointestinal adverse events was observed in participants receiving mazdutide in the 9 mg cohort, given the overall low incidence of these events in each study week and small sample size in each cohort, the observed difference should be evaluated in a larger study.

Due to potential heart rate increase upon glucagon receptor agonism, GLP-1 and glucagon receptor dual agonists may lead to more pronounced heart rate increase than GLP-1 agonists.^12^^,^^13^ The effect of mazdutide on heart rate was evidenced in the low-dose cohorts.^11^ With higher initiation doses and faster up-titration schemes, the high doses of 9 mg and 10 mg did not further increase the heart rate and the cardiac disorders were less frequently reported. Meanwhile, prominent and concurrent decrease in blood pressure may alleviate the cardiac burden and confer cardiovascular benefits.

With balanced GLP-1 receptor and glucagon receptor agonism, mazdutide demonstrated promising efficacy on body weight loss in Chinese adults with overweight or obesity. In low-dose cohorts, mazdutide dosed up to 6 mg cohorts achieved body weight loss up to 6.4%.^11^ In high-dose cohorts, mazdutide dosed with the 3-6-9 mg regimen achieved a placebo-adjusted 12-week body weight loss of 9.8%, which, to the best of our knowledge and at the time of writing, numerically surpassed any other single-agent anti-obesity treatments in terms of short-term body weight loss and has the potential to provide a comparable body weight loss benefit as bariatric surgery.^3^^,^^9^^,^^14^ The faster body weight loss achieved in the 9 mg cohort may be attributed to the faster up-titration with greater dose increment, while the relative potency of the two regimens should be evaluated in a long-term setting.

It is postulated that the effect of glucagon receptor stimulation might limit the glucose-lowering strength of GLP-1 and glucagon receptor dual agonists.^4^ Mazdutide was designed to balance the activation of GLP-1 receptor and glucagon receptor in order to avoid the glucagon receptor-induced hyperglycaemia while maintaining the desired effects of HbA_1c_ reduction and body weight loss. The glucose-lowering effect of low-dose mazdutide had been validated in another phase 1b study in Chinese patients with type 2 diabetes.^15^ While high-dose mazdutide showed promising efficacy on body weight loss, two non-diabetic participants receiving mazdutide had slight elevation in end-of-treatment HbA1c levels, and the safety and strength of these high-dose regimens in patients with insulin resistance and/or type 2 diabetes warrant further investigation.

Apart from the body weight loss and glucose-lowering efficacy, comprehensive metabolic improvements were frequently reported with GLP-1 receptor agonists and dual agonists. While the effect of mazdutide on lipids was essentially similar to those of GLP-1 receptor agonists, the reduction in serum uric acid concentration may be unique to the drug class.^16^ The reduction in serum uric acid was evident with both low-dose^11^ and high-dose mazdutide, while the potential underlying mechanism awaits further investigation.

The limitation of this study included small sample size and short duration of treatment. Due to limited access to the instruments in the study centres and limited number of participants in each cohort, the body fat content data were incomplete and not presented in this report. Moreover, analysis method of MMRM for efficacy outcomes of body weight, BMI and waist circumference was not pre-defined in the protocol and these analyses were informal. A phase 2 study had been launched to evaluate the efficacy and safety of mazdutide in all doses assessed in this phase 1b study in Chinese adults with overweight or obesity.

In summary, mazdutide dosed up to 9 mg or 10 mg was both well tolerated and showed a favourable safety profile. Mazdutide dosed with the 3-6-9 mg escalation regimen demonstrated robust efficacy on 12-week body weight loss, suggesting that mazdutide would be a promising body weight loss agent as the next generation GLP-1-based dual agonist.

## Contributors

LJ, LG, HD and LQ designed the study. LJ, LG, HJ, JY, LY, JW and CC did the trial and collected the data. JW, CC, HD, LF, BS, QM and LQ analysed the data. LJ, JW, CC, HD, LF, BS, QM and LQ interpreted the data. QM wrote the manuscript. LJ, LG and LQ assessed and verified the data. All authors had full access to all the data in the study and had critically reviewed the manuscript and approved the final manuscript. All authors vouch for data accuracy and fidelity to the protocol.

## Data sharing statement

The data supporting the analyses contained in the manuscript will be made available upon reasonable written request from researchers whose proposed use of the data for a specific purpose has been approved.

## Declaration of interests

LJ, LG, HJ, JY and LY received research funding from Innovent Biologics, Inc., during the conduct of the study. JW, CC, HD, LF, BS, QM and LQ were employees of Innovent Biologics, Inc.

## Appendix Supplementary materials

Image, application 1
