# Supplementary material for: Safety and efficacy of a GLP-1 and glucagon receptor dual agonist mazdutide (IBI362) 9 mg and 10 mg in Chinese adults with overweight or obesity: A randomised, placebo-controlled, multiple-ascending-dose phase 1b trial
Source: eClinicalMedicine. 2022 Oct 7;54:101691. doi: 10.1016/j.eclinm.2022.101691 (PMC9561728; doi:10.1016/j.eclinm.2022.101691)

Supplementary Materials

Safety and efficacy of a GLP-1 and glucagon receptor dual agonist mazdutide (IBI362) 9 mg and 10 mg in Chinese adults with overweight or obesity: a randomised, placebo-controlled, multiple-ascending-dose phase 1b trial

Fig. S1: Study design

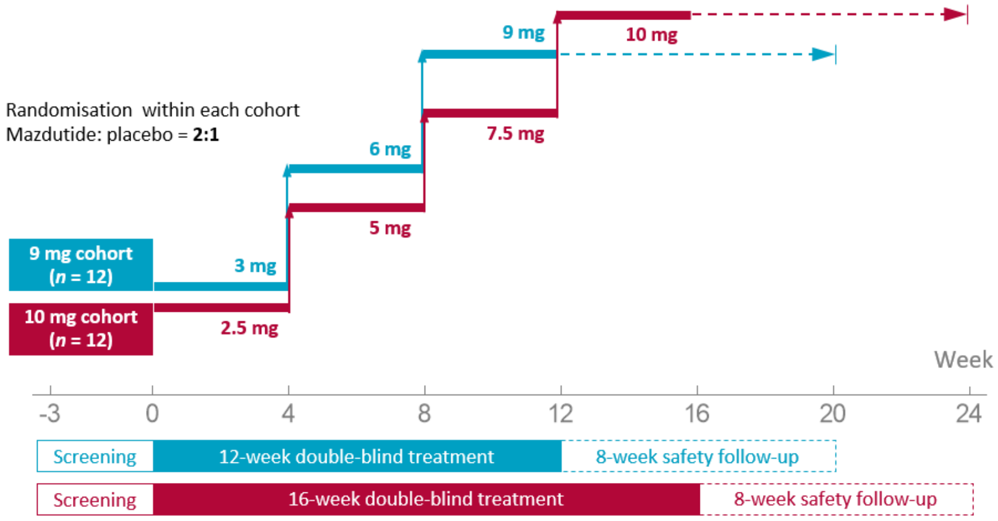

Fig. S2: By-week incidence of diarrhoea, nausea and vomiting in the 10 mg (a) and 9 mg (b) cohort.

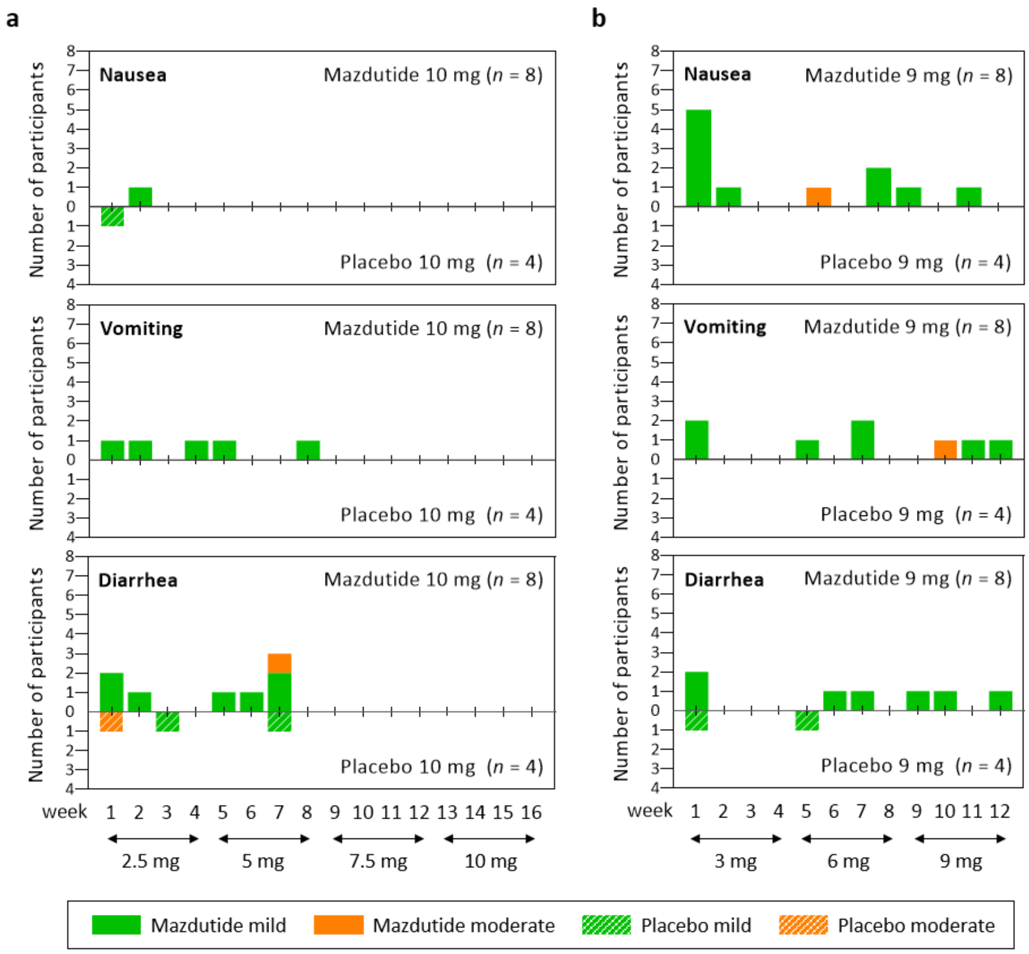

Fig. S3: Change from baseline in heart rate and blood pressure over time
Data are plotted as means +/− SE. CFB = change from baseline; DBP = diastolic blood pressure; SBP = systolic blood pressure; SE = standard error of the mean.

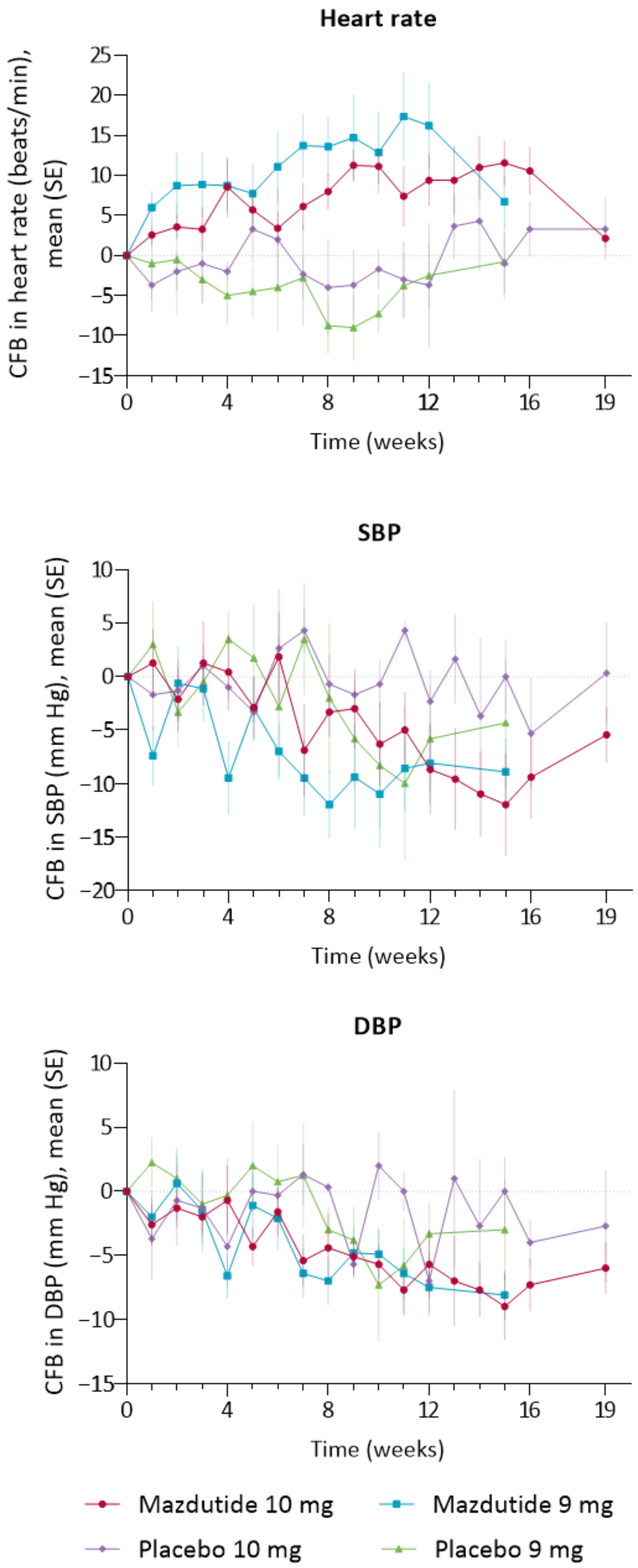

Fig. S4: HbA_{1c} levels at baseline, as well as CFB at week 12 (9 mg cohort) or week 16 (10 mg cohort) for each participant

CFB = change from baseline; HbA_{1c} = glycated haemoglobin A_{1c}.

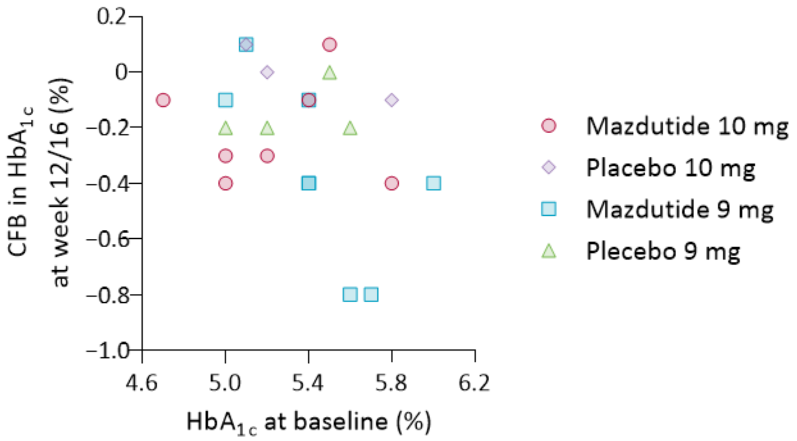

Fig. S5: Change from baseline in triglycerides, LDL cholesterol and total cholesterol levels over time

Data are plotted as individual values and means +/− SD. CFB = change from baseline; LDL = low density lipoprotein; SD = standard deviation.

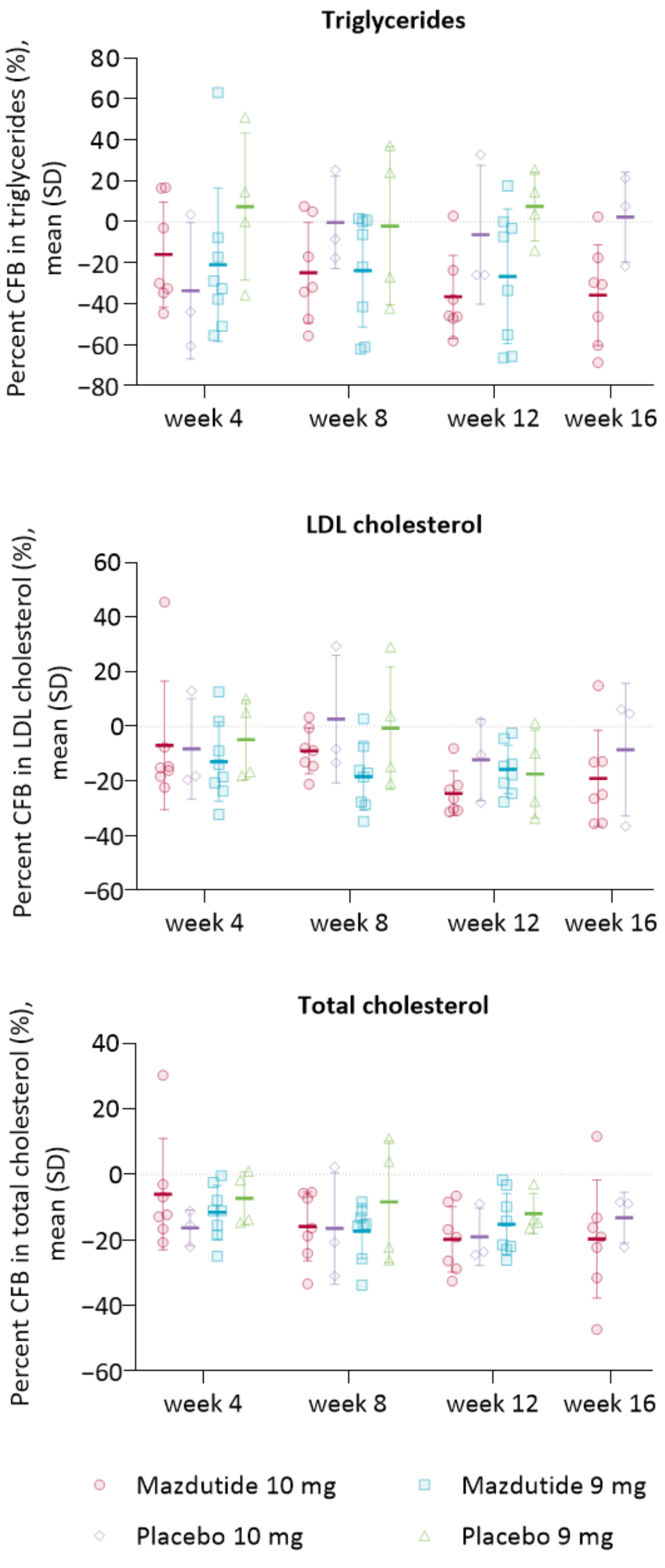

Fig. S6: Change from baseline in serum uric acid levels

Data are plotted as individual values and means +/− SD. CFB = change from baseline; SD = standard deviation.

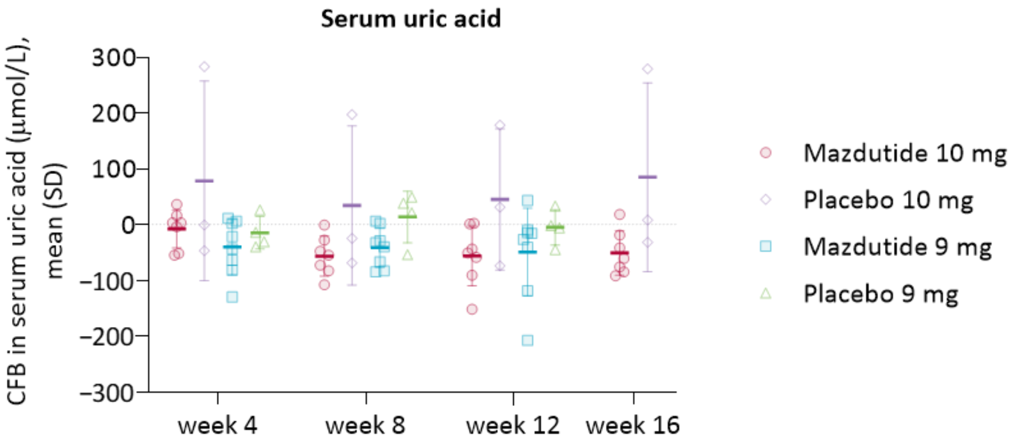

Table S1: Treatment-emergent adverse events

MedDRA System Organ Class<br>Preferred Term	10 mg cohort		9 mg cohort		Pooled placebo (n = 8)
	Mazdutide (n = 8)	Placebo (n = 4)	Mazdutide (n = 8)	Placebo (n = 4)	
**Individuals with ≥1 TEAE**	**8 (100)**	**4 (100)**	**8 (100)**	**3 (75.0)**	**7 (87.5)**
**Gastrointestinal disorders**	**5 (62.5)**	**4 (100)**	**7 (87.5)**	**3 (75.0)**	**7 (87.5)**
Vomiting	3 (37.5)	0	3 (37.5)	0	0
Nausea	1 (12.5)	1 (25.0)	5 (62.5)	0	1 (12.5)
Diarrhoea	4 (50.0)	2 (50.0)	2 (25.0)	2 (50.0)	4 (50.0)
Abdominal distension	0	2 (50.0)	4 (50.0)	1 (25.0)	3 (37.5)
Abdominal pain upper	1 (12.5)	0	0	0	0
Constipation	0	0	1 (12.5)	0	0
Eructation	1 (12.5)	0	0	0	0
Dyspepsia	0	0	1 (12.5)	1 (25.0)	1 (12.5)
Burning mouth syndrome	0	0	1 (12.5)	0	0
Flatulence	1 (12.5)	0	0	0	0
Abdominal discomfort	0	0	1 (12.5)	0	0
Abdominal pain	0	1 (25.0)	0	1 (25.0)	2 (25.0)
**Infections and infestations**	**5 (62.5)**	**2 (50.0)**	**6 (75.0)**	**2 (50.0)**	**4 (50.0)**
Upper respiratory tract infection	4 (50.0)	2 (50.0)	3 (37.5)	1 (25.0)	3 (37.5)
Urinary tract infection	2 (25.0)	0	4 (50.0)	1 (25.0)	1 (12.5)
Nasopharyngitis	1 (12.5)	0	0	0	0

MedDRA System Organ Class<br>Preferred Term	10 mg cohort		9 mg cohort		Pooled placebo (*n* = 8)
	Mazdutide (*n* = 8)	Placebo (*n* = 4)	Mazdutide (*n* = 8)	Placebo (*n* = 4)	
**Metabolism and nutrition disorders**	**3 (37.5)**	**2 (50.0)**	**5 (62.5)**	**2 (50.0)**	**4 (50.0)**
Decreased appetite	1 (12.5)	1 (25.0)	5 (62.5)	1 (25.0)	2 (25.0)
Hypokalaemia	1 (12.5)	0	1 (12.5)	0	0
Hyperuricemia	2 (25.0)	0	0	0	0
Hypoglycaemia	0	1 (25.0)	1 (12.5)	0	1 (12.5)
Hyperlipidaemia	1 (12.5)	0	0	1 (25.0)	1 (12.5)
**Investigations**	**2 (25.0)**	**0**	**4 (50.0)**	**1 (25.0)**	**1 (12.5)**
Alanine aminotransferase increased	1 (12.5)	0	1 (12.5)	0	0
Aspartate aminotransferase increased	1 (12.5)	0	1 (12.5)	0	0
C-reactive protein increased	0	0	1 (12.5)	0	0
Urobilinogen urine increased	0	0	1 (12.5)	0	0
Blood urine present	0	0	1 (12.5)	0	0
White blood cells urine positive	1 (12.5)	0	0	0	0
Lipase increased	1 (12.5)	0	0	1 (25.0)	1 (12.5)
Lipoprotein (a) increased	1 (12.5)	0	0	0	0
Platelet count increased	0	0	1 (12.5)	0	0
**Nervous system disorders**	**0**	**0**	**3 (37.5)**	**0**	**0**
Dizziness	0	0	1 (12.5)	0	0
Headache	0	0	1 (12.5)	0	0

MedDRA System Organ Class<br>Preferred Term	10 mg cohort		9 mg cohort		Pooled placebo (*n* = 8)
	Mazdutide (*n* = 8)	Placebo (*n* = 4)	Mazdutide (*n* = 8)	Placebo (*n* = 4)	
Paraesthesia	0	0	1 (12.5)	0	0
Hypoesthesia	0	0	1 (12.5)	0	0
**General disorders and administration site conditions**	**0**	**0**	**2 (25.0)**	**2 (50.0)**	**2 (25.0)**
Asthenia	0	0	1 (12.5)	1 (25.0)	1 (12.5)
Fatigue	0	0	1 (12.5)	1 (25.0)	1 (12.5)
**Musculoskeletal and connective tissue disorders**	**0**	**0**	**1 (12.5)**	**0**	**0**
Back pain	0	0	1 (12.5)	0	0
**Respiratory, thoracic and mediastinal disorders**	**0**	**1 (25.0)**	**1 (12.5)**	**1 (25.0)**	**2 (25.0)**
Hiccups	0	1 (25.0)	1 (12.5)	1 (25.0)	2 (25.0)
**Cardiac disorders**	**0**	**1 (25.0)**	**1 (12.5)**	**0**	**1 (12.5)**
Ventricular extrasystoles	0	1 (25.0)	1 (12.5)	0	1 (12.5)
**Eye disorders**	**1 (12.5)**	**0**	**0**	**0**	**0**
Vision blurred	1 (12.5)	0	0	0	0
**Psychiatric disorders**	**0**	**0**	**1 (12.5)**	**0**	**0**
Mental fatigue	0	0	1 (12.5)	0	0
**Hepatobiliary disorders**	**0**	**0**	**1 (12.5)**	**0**	**0**
Cholelithiasis	0	0	1 (12.5)	0	0
**Blood and lymphatic system disorders**	**0**	**0**	**1 (12.5)**	**0**	**0**
Anaemia	0	0	1 (12.5)	0	0

MedDRA System Organ Class<br>Preferred Term	10 mg cohort		9 mg cohort		Pooled placebo<br>(*n* = 8)
	**Mazdutide (*n* = 8)**	**Placebo (*n* = 4)**	**Mazdutide (*n* = 8)**	**Placebo (*n* = 4)**	
**Vascular disorders**	**0**	**0**	**1 (12.5)**	**0**	**0**
Hypertension	0	0	1 (12.5)	0	0
**Skin and subcutaneous tissue disorders**	**0**	**0**	**0**	**1 (25.0)**	**1 (12.5)**
Hyperhidrosis	0	0	0	1 (25.0)	1 (12.5)

By MedDRA (version 24.0). Data are presented n (%).

MedDRA = The Medical Dictionary for Regulatory Activities. TEAE = treatment-emergent adverse event.

Clinical Study Protocol

**Study Title:**	Multiple Dose Tolerability and Pharmacokinetic/Pharmacodynamic Study of IBI362 in Overweight or Obese Chinese Subjects
**Protocol Number:**	CIBI362B101
**Version date and version number:**	Version 2.1, 14 Dec 2020
**Product Name:**	IBI362 (glucagon-like peptide-1 receptor/glucagon receptor (GLP-1R/GCGR) dual agonist)
**Study Phase:**	Phase Ib/II
**Sponsor:**	Innovent Biologics (Suzhou) Co., Ltd.<br>168 Dongping Street, Suzhou Industrial Park, Jiangsu Province, China
**Sponsor Contact:**	Lei Qian, Senior Medical Director<br>021-31837215<br>lei.qian@innoventbio.com

Confidentiality Statement

This document contains confidential information of Innovent Biologics (Suzhou) Co., Ltd.

The contents of this document should not be disclosed to anyone other than the investigator, study consultant or associated personnel, Institutional Review Board/Independent Ethics Committee.

The information in this document may not be used for any purpose other than the evaluation or conduct of this clinical study without written permission from the Sponsor.

Investigator Signature Page

Study Title: Multiple Dose Human Tolerability and Pharmacokinetic/Pharmacodynamic Study of IBI362 in Overweight or Obese Chinese Subjects

Protocol Number: CIBI362B101

This protocol is a trade secret of Innovent Biologics (Suzhou) Co., Ltd. I have read and fully understand this protocol and undertake to conduct this study in accordance with this protocol and the requirements of Good Clinical Practice and in compliance with applicable laws and regulations and the Declaration of Helsinki. At the same time, I undertake not to disclose any confidential information in this study to any third party without the written consent of Innovent Biologics (Suzhou) Co., Ltd.

Instructions for Investigators: Please sign and date this signature page, print the investigator's name, professional title and the name of the study site, and return to Innovent Biologics (Suzhou) Co., Ltd. After signing.

I have read the entire contents of this protocol and warrant that this study will be conducted as required:

Investigator Signature: ____________ Date: ________

Printed Name: _____________________________

Title of Investigator: _____________________________

Site Name/Address: __________________________

Sponsor Signature Page

Study Title: Multiple Dose Human Tolerability and Pharmacokinetic/Pharmacodynamic Study of IBI362 in Overweight or Obese Chinese Subjects

Protocol Number: CIBI362B101

Title	Name	Signature (Print)	Date
Senior Medical Director	Lei Qian	____________________	____________________
Senior Director, Biostatistics	Xing Sun	____________________	____________________

Protocol Summary

**Protocol Number**	CIBI362B101
**Sponsor**	Innovent Biologics (Suzhou) Co., Ltd.
**Investigational drug**	IBI362
**Study Title**	Multiple Dose Tolerability and Pharmacokinetic/Pharmacodynamic Study of IBI362 in Overweight or Obese Chinese Subjects
**Staging**	Phase Ib/II
**Version No./Date**	Version 2.1, 14 Dec 2020
**Planned number of cases**	60 participants
**Subject Population**	Overweight or obese subjects with less than 5% weight change controlled by diet and exercise for at least 12 weeks.
**Study Objectives**	**Primary Objective**<br>• To investigate the safety and tolerability of multiple subcutaneous injections of IBI362 in overweight or obese subjects and to determine the safe dose range for clinical use.<br>**Secondary Objectives**<br>• To investigate the pharmacokinetic/pharmacodynamic (PK/PD) parameters of multiple subcutaneous injections of IBI362 in overweight or obese subjects.
**Study Endpoints**	**Primary Endpoint**<br>• Safety and tolerability: incidence of various adverse events (including complaints of subjects, physical examination, laboratory tests (hematology, blood biochemistry, blood lipids, coagulation function, urinalysis, myocardial enzymes, serum amylase and lipase, thyroid function, serum calcitonin, etc.), vital signs (pulse, respiration, blood pressure, body temperature), 12-lead ECG abnormalities and hypoglycemic events, etc.) in different dose periods during the escalation process, The name, clinical characteristics, severity, onset and end time, treatment and outcome of adverse events were recorded, the correlation between adverse events and the study drug was determined, and the medication compliance was analyzed.

	• Maximum tolerated dose: if "dose escalation stopping criteria" are met at a given dose level and dose exploration is stopped, the previous dose is the maximum tolerated dose; If the "dose escalation stopping criteria" are still not met when the maximum escalation dose is reached, the maximum tolerated dose is greater than or equal to the maximum escalation dose.<br>**Secondary Endpoints**<br>• To assess the pharmacokinetic parameters of IBI362 in overweight or obese subjects, including but not limited to: T_{max}, C_{max}, Area Under Curve (AUC), volume of distribution (V_d), half-life ($T_{1/2}$), clearance (CL), accumulation coefficient (AR);<br>• To evaluate the pharmacodynamic parameters of fasting plasma glucose, fasting glucagon, fasting insulin, fasting C-peptide, endogenous oxyntomodulin (OXM) and Glucagon Like Peptide 1 (GLP-1) before and after administration of multiple doses of IBI362;<br>• Changes from baseline in fasting body weight, waist-to-hip ratio, BMI, blood pressure, pulse rate, blood lipids and HbA_{1c} at steady state in each dose period during dose escalation;<br>• Occurrence of anti-drug antibody (ADA) and neutralizing antibody (NAb) against IBI362 in serum before and after administration;<br>• Pancreatic β-cell function and insulin resistance (HOMA model) were evaluated.<br>• To assess the effect of IBI362 on serum uric acid and alanine aminotransferase in overweight or obese subjects.<br>**Exploratory Endpoints**<br>• Evaluate the total body fat content, waist fat content, hip fat content and waist-hip fat ratio measured by dual-energy X-ray absorptiometry (DEXA), calculate lean body mass (lean mass = body weight-fat weight), and compare the changes of each indicator from baseline after 12 weeks and 16 weeks of administration;<br>• Evaluate intra-abdominal fat area (VFA), subcutaneous fat area (SFA) and total abdominal fat area (TFA) measured by MRI, and compare the changes of each index from baseline after 12 weeks and 16 weeks of administration.
**Study Design**	This is the first study to assess the safety, tolerability, and PK/PD of multiple injections of IBI362 in Chinese overweight or obese subjects. A multicenter, randomized, double-blind (subject, investigator) and placebo-controlled trial design was used. Thirty-six overweight or obese subjects (Cohort 1, Cohort 2, and

Cohort 3) controlled with diet and exercise for at least 12 weeks with a weight change of less than 5% have been enrolled in this study, and all subjects were safe and well tolerated (see Sections 1.3. 2 and 1.3. 3 for details). Based on the current PK data of subjects in Cohorts 1-3 showing that the plasma concentration in Chinese overweight/obese population is lower than the prespecified plasma concentration in the study, two additional cohorts 4 and 5 will be further explored based on the PK data of IBI362 in China, with 12 subjects in each cohort and an estimated total of 24 subjects. The entire trial period consisted of a 3-week screening period, a 12-to 16-week double-blind treatment period, and an 8-week follow-up period after the last dose.1.3.21.3.3

The double-blind treatment phase was divided into five cohorts, Cohort 1 (n=12), Cohort 2 (n=12), Cohort 3 (n=12), Cohort 4 (n=12), and Cohort 5 (n=12), and subjects in each cohort were randomized in a 2: 1 ratio to IBI362 treatment (n=8) and placebo (n=4). The SC dosing regimens of IBI362 or placebo in Cohort 1, Cohort 2, Cohort 3, Cohort 4, and Cohort 5 are described as follows:

Cohort 1

The starting dose is 1.0 mg once weekly for consecutive 4 weeks. If the subject is well tolerated #, the dose will be up-titrated to 2.0 mg once weekly for consecutive 4 weeks. The tolerability will be observed again. If the subject is well tolerated #, the dose will be up-titrated to 3.0 mg once weekly for consecutive 4 weeks. (The dose will be increased by 1mg every 4 weeks to the target dose).

Cohort 2

The starting dose is 1.5 mg, administered once a week for consecutive 4 weeks. If the subject is well tolerated #, the dose will be up-titrated to 3.0 mg, administered once a week for consecutive 4 weeks. The tolerability of the subject will be observed again. If the subject is well tolerated #, the dose will be up-titrated to 4.5 mg, administered once a week for consecutive 4 weeks (the dose will be increased by 1.5 mg every 4 weeks to the target dose).

If subjects in Cohort 2 are intolerant to 3.0 mg or 4.5 mg, the dose should be adjusted according to the criteria in Table 1.**1**

Cohort 3

The subjects in Cohort 2 can only start the administration after the subjects in Cohort 2 have completed the administration of 1.5 mg for 4 weeks and are well tolerated; If 1.5 mg is not tolerated by subjects in Cohort 2, 2.0 mg and higher will not be explored for subjects in Cohort 3.

In this cohort, the starting dose is 2.0 mg once weekly for consecutive 4 weeks. If the subject has good tolerance #, the dose will be increased to 4.0 mg once weekly for consecutive 4 weeks. The tolerability will be observed again. If

	the subject has good tolerance #, the dose will be increased to 6.0 mg once weekly for consecutive 4 weeks. (The dose will be increased by 2mg every 4 weeks to the target dose).<br>**Cohort 4**<br>The starting dose of the subjects is 2.5 mg, once a week, after continuous administration for 4 weeks; If the subject is well tolerated #, the dose will be up-titrated to 5.0 mg once a week for consecutive 4 weeks; Observe the tolerance of the subjects again. If the tolerance is good #, the dose will be increased to 7.5 mg once a week for consecutive 4 weeks; Observe the tolerability of the subjects again. If the tolerability is good #, the dose will be increased to 10.0 mg once a week for consecutive 4 weeks (the dose will be increased by 2.5 mg every 4 weeks to the target dose).<br>**Cohort 5**<br>The starting dose of the subjects is 3.0 mg, once a week, after continuous administration for 4 weeks; If the subject is well tolerated #, the dose will be up-titrated to 6.0 mg, once a week for consecutive 4 weeks; Observe the tolerability of the subjects again. If the tolerability is good #, the dose will be increased to 9.0 mg once a week for consecutive 4 weeks (increase the dose by 3.0 mg every 4 weeks to the target dose).<br>(# Refer to Section 3.2. 1 for single subject intolerance criteria)<br>**(I) Stop criteria for dose escalation: If any of the following criteria are met, the dose escalation will be terminated.**<br>• Heart rate > 100 beats per minute occurred in > 50% of subjects taking IBI362 within the same dose.<br>• Occurrence of at least one treatment-related serious adverse event (other than those expected to be related to the pharmacological properties of IBI362, such as hypoglycemia).<br>• "No less than 50% of subjects experienced symptomatic hypoglycemic events with plasma glucose levels < 2.8 mmol/L at a given dose level, and these events were considered related to IBI362."<br>• No less than 2 subjects who receive IBI362 develop persistent (more than one week) symptoms characteristic of acute pancreatitis.<br>• Non-serious AEs of severe intensity that are related to IBI362 (refer to Section 7.3 of the protocol) occurring in no less than 2 subjects in the same dose group, regardless of whether or not the event is associated with the same organ or system (other than gastrointestinal effects).<br>**Note: In case of AE related to dose escalation stop, please report to the sponsor as per Appendix 6.**

	5<br>**(II) Intolerance criteria for a single subject:**<br>• If venous plasma glucose < 2.8 mmol/L; Or if the venous plasma glucose does not reach the standard of < 2.8 mmol/L, but the symptoms of hypoglycemia are obvious and cannot be recovered spontaneously (within 15min), and the re-test before the next dose still meets the above standard, it is considered that the patient is intolerant at this dose;<br>• If there are other intolerance conditions related to the study drug that the investigator considers to be related to the study drug, and the investigator thinks it is necessary to stop the exploration of the subject at this dose after discussion with the sponsor, this case is considered to be intolerable at this dose.
**Main Inclusion Criteria**	1. Age 18 ~ 75 years (both inclusive), male or female;<br>Obese: BMI ≥ 28.0 kg/m^2; Or overweight: 24 ≤BMI < 28.0 kg/m^2 with at least one of the following manifestations: i. Strong appetite, unbearable hunger before meal, and more food intake per meal; ii. Patients with one or more of pre-diabetes (impaired fasting glucose and/or impaired glucose tolerance), hypertension, dyslipidemia (see appendix 4 for reference standards), and fatty liver (within 6 months prior to screening); iii. Combined weight-bearing joint pain; iv. Obesity-induced dyspnea or obstructive sleep apnea syndrome;<br>2. 3<br>3. Controlled by diet and exercise alone for at least 12 weeks at screening, with body weight change of less than 5%;<br>4. Able to understand the procedures and methods of this study, willing to strictly comply with the clinical trial protocol to complete this trial, and voluntarily sign the informed consent form.
**Main Exclusion Criteria**	1. Subjects who are suspected by the investigator to be allergic or have allergic constitution to the study drug or ingredients;<br>2. Use of any of the following medications or treatments prior to screening:<br>1) Previous use of GLP-1 receptor (GLP-1R) agonists or GLP-1R/GCGR agonists;<br>2) Use of drugs that affect body weight within 3 months prior to screening, including systemic steroids (intravenous, oral or intra-articular administration), metformin, SGLT2 inhibitors, thiazolidinediones (TZDs), tricyclic antidepressants, psychiatric drugs or sedative drugs (such as imipramine, amitriptyline, mirtazapine, paroxetine, phenelzine,

	chlorpromazine, thioridazine, clozapine, olanzapine, valproic acid, valproic acid derivatives, lithium salts);<br>3) Use of Chinese herbal medicine or health products affecting body weight within 3 months prior to screening;<br>4) Have used or are currently using weight loss drugs within 3 months prior to screening, such as: sibutramine hydrochloride, orlistat, phentermine, phenylpropanolamine, chlorpheniramine, phentermine, bupropion, lorcaserin, phentermine/topiramate mixture, naltrexone/bupropion mixture, etc.;<br>5) Participation in other clinical trials (treated with an investigational drug) within 3 months prior to screening.<br>3. History or evidence of any of the following prior to screening:<br>1) Subjects diagnosed with diabetes mellitus according to WHO1999 criteria;<br>2) Fasting venous blood glucose ≥ 7. 0 mmol/L at screening or venous blood glucose ≥ 11. 1 mmol/L 2 hours after 75g oral glucose tolerance test (OGTT) glucose load (for subjects with fasting blood glucose of 6.1-7.0 mmol/L at screening, venous blood glucose 2 hours after OGTT glucose load should be collected for confirmation);<br>3) Subjects with retinopathy in the past or at screening;<br>4) Obesity caused by secondary diseases or drugs, including: increased cortisol hormone (such as Cushing's syndrome), obesity caused by pituitary gland and hypothalamus injury, obesity caused by reduction/withdrawal of weight-reducing drugs, etc.;<br>5) Previous bariatric surgery or acupuncture for weight loss within 1 year before screening;<br>6) History of depression in the past; Or have a history of severe mental illness in the past, such as: schizophrenia, bipolar disorder, etc.;<br>7) Uncontrolled hypertension at screening after treatment with antihypertensive drugs for at least 4 weeks, defined as: systolic blood pressure > 140mmHg and/or diastolic blood pressure > 100mmHg;<br>8) Systolic blood pressure < 90mmHg and/or diastolic blood pressure < 50mmHg at screening;<br>9) History of malignancy (except cured basal cell carcinoma of the skin and carcinoma in situ of the cervix) at the time of screening;<br>10) Heart-related diseases (such as angina pectoris, myocardial infarction, cardiomyopathy, acute and chronic heart failure, etc.) at screening;<br>11) Hemorrhagic or ischemic stroke or transient ischemic attack within 6 months prior to screening;

	12) History of thyroid C-cell carcinoma, MEN (multiple endocrine neoplasia) 2A or 2B syndrome, or relevant family history at screening;<br>13) History of acute or chronic pancreatitis, gallbladder disease, or pancreatic injury at screening;<br>14) Chronic gastrointestinal disease, systemic disease that may affect gastrointestinal motility at screening, or use of drugs that may alter gastrointestinal motility, appetite or absorption within 3 months prior to screening;<br>15) Existence of limb deformity or disability, unable to accurately determine height, weight and other indicators;<br>16) Major and medium-sized surgery, severe trauma, severe infection within 1 month prior to screening, which is not suitable for participation in the study as judged by the investigator;<br>17) Previous suicidal tendency or suicidal behavior;<br>18) Anticipated surgery during the trial, except for outpatient surgery that has no effect on the safety of subjects and trial results as judged by the investigator;<br>19) Subjects who are positive for human immunodeficiency virus (HIV) antibody or hepatitis B surface antigen (HBsAg) or hepatitis C (HCV) antibody or syphilis antibody at screening;<br>20) History of alcohol abuse within 1 month prior to screening. Average weekly alcohol intake of more than 21 units for men and 14 units for women, or unwillingness to stop drinking 24 hours before the dosing day and throughout the study (1 unit = 360ml of beer, or 150ml of red wine, or 45ml of distilled spirits/liquor);<br>21) Positive urine screening test for drugs and drugs of abuse at screening.<br>4. Any laboratory test indicator meeting the following criteria (if there is a clear reason for retest at screening, it can be retested within one week, and the investigator should record the reason for retest):<br>1) Serum calcitonin ≥ 15 ng/L at screening;<br>2) Alanine aminotransferase ≥ 2.0 × ULN and/or aspartate aminotransferase ≥ 2.0 × ULN and/or total bilirubin ≥ 1.0 × ULN and/or alkaline phosphatase ≥ 2.0 × ULN at screening;<br>3) Glomerular filtration rate eGFR < 60 mL/min/1. 73 m^2 at screening, as estimated by the CKD-EPI equation (see Appendix 2)<br>4) Abnormal thyroid function (FT3, FT4, or TSH) at screening;<br>5) Fasting triglycerides ≥ 5. 64 mmol/L (500 mg/dl) at screening, if the subject is on lipid-modifying therapy, the drug dose must be stable for 30 days prior to screening;

	6) Blood amylase or lipase > 2.0 × ULN at screening;<br>7) International normalized ratio (INR) of prothrombin time greater than the upper limit of normal at screening.<br>5. Heart rate < 50 beats/min or > 90 beats/min on 12-lead ECG at screening; Subjects with the following clinically significant 12-lead electrocardiograms (ECGs) abnormalities at screening: second or third degree atrioventricular block without a pacemaker, long QT syndrome or QTcF > 450ms (see Appendix 3 for calculation formula), PR interval < 120ms or PR interval > 220ms, QRS > 120ms, left or right bundle branch block, pre-excitation syndrome, or serious arrhythmia requiring treatment;<br>6. 2<br>7. Pregnant or lactating females, males or females of childbearing potential not willing to use contraception throughout the study;<br>8. Blood donation and/or blood loss ≥ 400 mL or bone marrow donation within 3 months prior to screening, or presence of hemoglobinopathy, hemolytic anemia, sickle cell anemia, or hemoglobin < 110g/L (male) or < 100g/L (female);<br>9. The subject has any other factors that may affect the efficacy or safety evaluation of this study, and is not suitable for participation in this study in the opinion of the investigator.
**Study Drug Strength and Mode of Administration**	**Drug strength: The drug product strength is 2 mg/vial, and the placebo strength matches the drug product;**<br>**Mode of administration: subcutaneous injection, once weekly,**<br>• Cohort 1: 1.0 mg for 4 weeks + 2.0 mg for 4 weeks + 3.0 mg for 4 weeks;<br>• Cohort 2: 1.5 mg for 4 weeks + 3.0 mg for 4 weeks + 4.5 mg for 4 weeks;<br>• Cohort 3: 2.0 mg for 4 weeks + 4.0 mg for 4 weeks + 6.0 mg for 4 weeks;<br>• Cohort 4: 2.5 mg for 4 weeks + 5.0 mg for 4 weeks + 7.5 mg for 4 weeks + 10.0 mg for 4 weeks;<br>• Cohort 5: 3.0 mg for 4 weeks + 6.0 mg for 4 weeks + 9.0 mg for 4 weeks.
**Statistical Methods**	**This was a multicenter, randomized, double-blind (subject, investigator), placebo-controlled study.**<br>**Statistical Analysis Methods**<br>Descriptive statistics will only be performed for efficacy endpoints in this study. For continuous efficacy endpoints, the corresponding mean and two-sided 95% confidence interval will be calculated for IBI362 subjects and all placebo subjects within each dose group, respectively. The p-value for each dose group of IBI362 versus placebo will be calculated using a two-sample t-test and the corresponding point estimate and 95% confidence interval for the difference will be presented. Missing values will be imputed using the LOCF method. The rates

	within each dose group will be calculated and 95% confidence intervals will be calculated using Clopper-Pearson for the typed efficacy endpoints, and the chi-square test will be used to compare the differences between IBI362 and placebo groups and the corresponding 95% confidence intervals will be calculated. Missing values for typing endpoints will be imputed using the default non-response method.<br>**Safety Analysis**<br>The number of subjects with each AE was summarized by Medical Dictionary for Regulatory Activities (MedDRA) system organ class, MedDRA preferred term, and adverse event grade. The number and percentage of subjects with each category of adverse events (including causality, severity, SAE, etc.) will be summarized, and the events within each category will be further summarized by MedDRA system organ class and preferred term.<br>Safety indicators include: laboratory tests, ECG, vital signs, immunogenicity indicators, hypoglycemic events and injection site reactions.<br>**Laboratory Tests**<br>Measured and changed values of hematology, blood biochemistry, blood lipid, coagulation function, urinalysis, myocardial enzyme spectrum, blood amylase and lipase, thyroid function, serum calcitonin and fasting plasma glucose (tested by central laboratory) before and after treatment were described using mean ± standard deviation, maximum, minimum and median, and normal and abnormal changes before and after treatment were described using cross classification table.<br>Urinalysis: cross classification table will be used to describe the changes of normal and abnormal before and after treatment.<br>Describe the proportion of "abnormal and clinically significant" among subjects with abnormal changes, where the abnormality is clinically significant or not as judged by the investigator.<br>**Other**<br>Details of subjects who died were listed.<br>Describe ECG measurements and changes. The changes between normal and abnormal before and after treatment will be described using cross-categorical tables. Descriptive statistics will be provided for changes in vital signs of subjects.<br>**Pharmacokinetics and pharmacodynamics**<br>Each PK parameter, including but not limited to T_{max}, C_{max}, product under the concentration-time curve (AUC), volume of distribution (V_d), half-life ($T_{1/2}$),

	clearance (CL) and accumulation coefficient (AR), will be summarized separately for each dose group.<br>Pharmacodynamic parameters, including fasting plasma glucose, fasting glucagon, fasting insulin, fasting C-peptide, endogenous OXM, and GLP-1, were summarized by group at baseline and at each time point after dosing, and changes from baseline at each time point were summarized.<br>The changes from baseline in fasting body weight, waist-to-hip ratio, BMI, blood pressure, pulse rate and blood lipids before administration and at 4 weeks (D29), 8 weeks (D57), 12 weeks (D85) and 16 weeks (D113) after administration were summarized and analyzed by groups. The change from baseline in HbA_{1c} at pre-dose and at 12 and 16 weeks post-dose (D113) was analyzed in a pooled manner.<br>Changes from baseline in islet function (HOMA model) at each time point after treatment were summarized by group.<br>Changes from baseline in serum uric acid and alanine aminotransferase levels at each time point after treatment were summarized by group.<br>**Immunogenicity**<br>The occurrence of anti-IBI362 antibodies (ADA) and neutralizing antibodies (NAb) in the serum of subjects before and after dosing will be summarized.<br>**Exploratory Analysis**<br>Changes from baseline in lean body mass (lean mass = body mass-fat mass) were summarized for total body fat content, waist fat content, hip fat content, and waist-to-hip fat ratio measured by dual-energy X-ray absorptiometry (DEXA) and for intra-abdominal fat area (VFA), subcutaneous fat area (SFA), and total abdominal fat area (TFA) measured by MRI after 12 and 16 weeks of dosing.

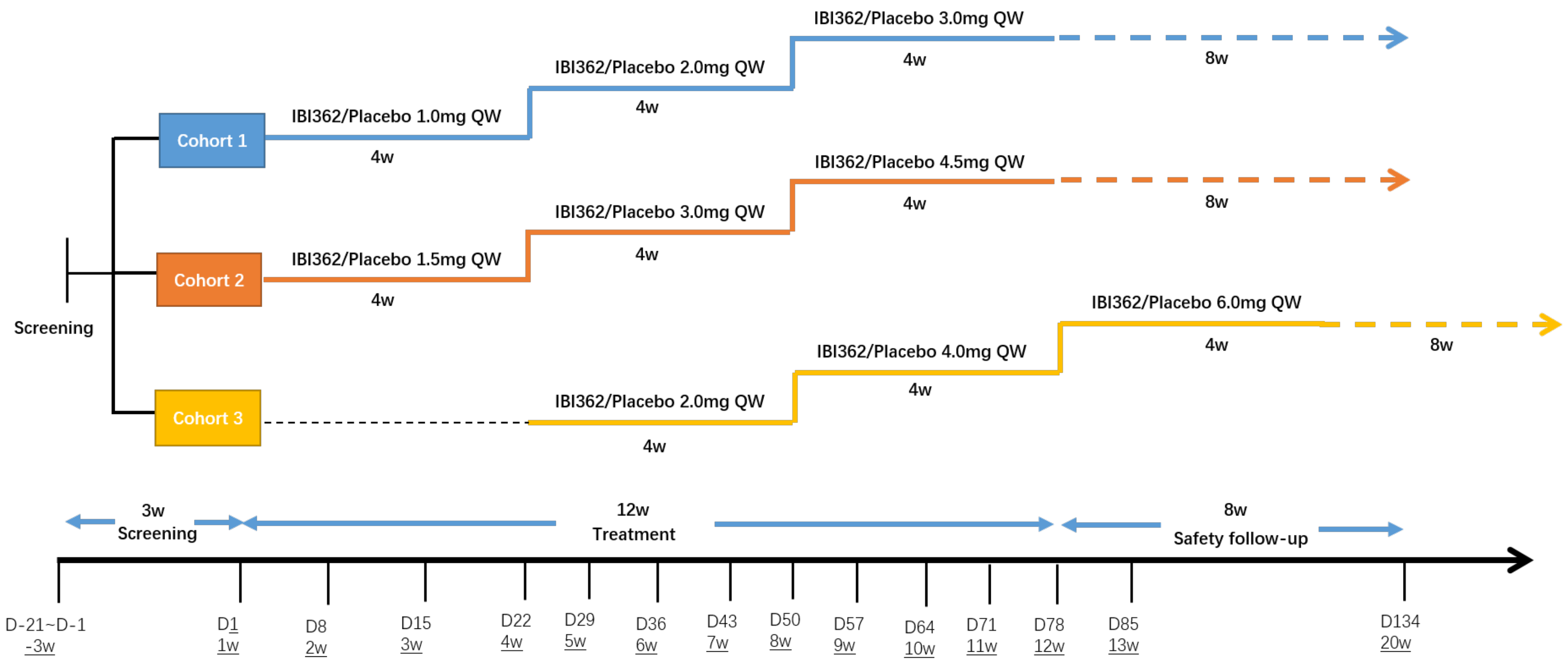

1Figure 1. Study Trial Design Diagram (Cohort 1, Cohort 2, and Cohort 3)

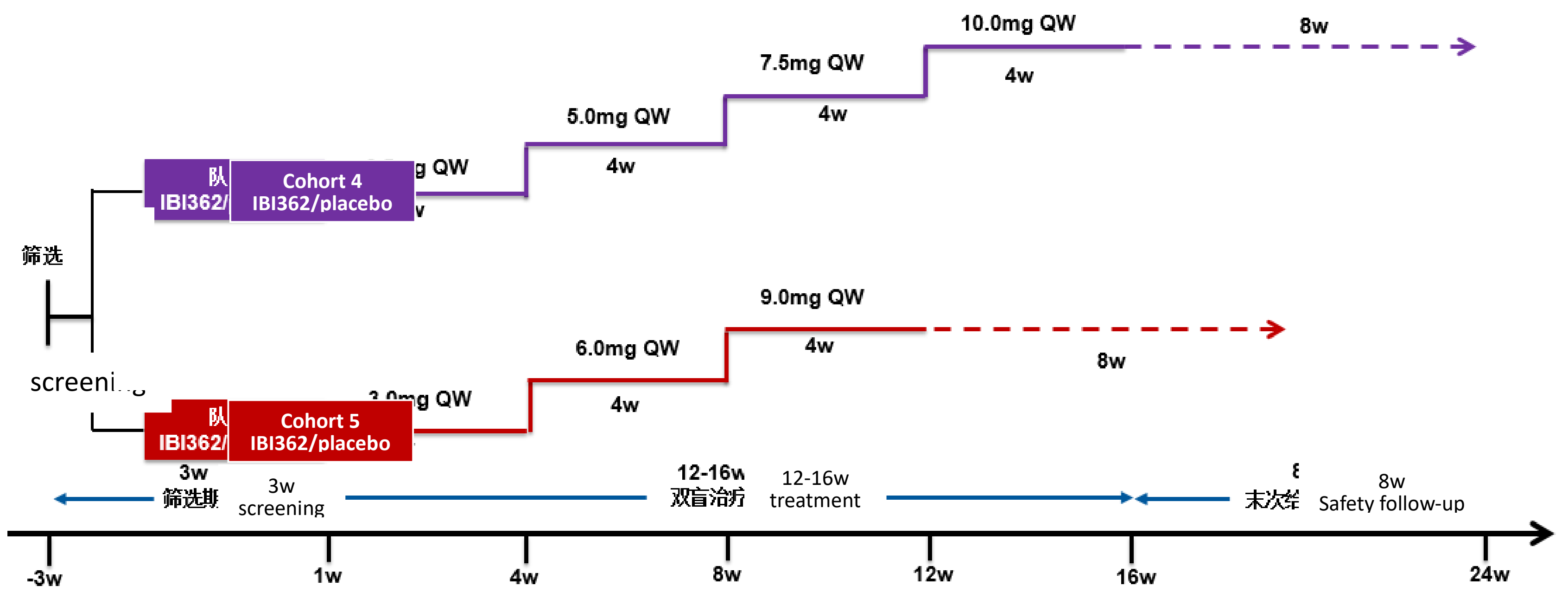

2Figure 2. Study Design Diagram (Cohort 4 and Cohort 5)

1Table 1. Reference Table for Dose and Adjustment during Escalation (Cohort 1, Cohort 2 and Cohort 3)

**Weeks**		**1**	**2**	**3**	**4**	**5**	**6**	**7**	**8**	**9**	**10**	**11**	**12**				
Cohort 1 (N=12)	IBI362/Placebo	1.0 mg QW				2.0 mg QW				3.0 mg QW							
**Weeks**		**1**	**2**	**3**	**4**	**5**	**6**	**7**	**8**	**9**	**10**	**11**	**12**				
Cohort 2 (N=12)	IBI362/Placebo	1.5 mg QW				3mg QW				4.5 mg QW							
**Weeks**		**1**	**2**	**3**	**4**	**5**	**6**	**7**	**8**	**9**	**10**	**11**	**12**				
Cohort 2 (backup 1) *	IBI362/Placebo	1.5 mg QW				3mg	0mg	2.25 mg	2.25 mg	3mg QW							
**Weeks**		**1**	**2**	**3**	**4**	**5**	**6**	**7**	**8**	**9**	**10**	**11**	**12**				
Cohort 2 (backup 2) #	IBI362/Placebo	1.5 mg QW				3mg QW				4.5 mg	0mg	3.75 mg	3.75 mg				
**Weeks**		**-4**	**-3**	**-2**	**-1**	**1**	**2**	**3**	**4**	**5**	**6**	**7**	**8**	**9**	**10**	**11**	**12**
Cohort 3 (N=12) &	IBI362/Placebo					2.0 mg QW				4.0 mg QW				6.0 mg			

Note:

* If the subjects in Cohort 2 cannot tolerate 3mg during escalation, the dose should be reduced to 2.25 mg after 1 week of discontinuation in Cohort 2 (backup1), and subsequent dose escalation will be performed in Cohort 2 (backup1);

# If the subjects in cohort 2 cannot tolerate 4.5 mg during the escalation process, the dose should be reduced to 3.75 mg after 1 week of discontinuation as in cohort 2 (backup2), and the dose should be maintained until the end of the study;

& Subjects in Cohort 3 will not start dosing until subjects in Cohort 2 complete the 4-week tolerance test of 1.5 mg. If subjects in Cohort 2 cannot tolerate 1.5 mg, the dose of 2.0 mg and higher will not be explored for subjects in Cohort 3.

3.2.1Δ Intolerance criteria for a single subject refer to protocol Section 3.2. 1.

2Table 2. Trial Procedures and Schedule of Assessments (Cohort 1, Cohort 2, and Cohort 3)

**Study Phases and Procedures**	**Screening Period [1]**	**Double-Blind Treatment Period [2]**																			**Follow-up**	**Early Withdrawal**
Study Visits	V1	V2	V3	V4	V5	V6	V7	V8	V9	V10	V11	V12	V13	V14	V15	V16	V17	V18	V19	V20	V21	
Study duration (weeks)	-3 ~ 0	1	2	3	4		5		6	7	8		9		10	11	12			13	20	
Study Duration (days)	-21 ~-1	1	8	15	22	23	29	30	36	43	50	51	57	58	64	71	78	79	80	85	134	
Window (days)	-	-	± 2	± 2	± 2	-	± 2	-	± 2	± 2	± 2	-	± 2	-	± 2	± 2	± 2	-	-	± 2	± 3	
Informed Consent	X																					
Inclusion/Exclusion Criteria	X																					
Demographics/Past Medical History/Prior Therapy	X																					
Vital signs[3]	X	X	X	X	X	X	X	X	X	X	X	X	X	X	X	X	X	X	X	X	X	X
Physical Examination[4]	X	X	X	X	X		X		X	X	X		X		X	X	X			X	X	X
Height	X																					
Weight [5]	X	X	X	X	X	X	X	X	X	X	X	X	X	X	X	X	X	X	X	X	X	X
Waist circumference [6]	X	X					X						X							X		X
Hip circumference [7]	X	X					X						X							X		X
Laboratory tests[8]	X						X						X							X		X
Alcohol breath test	X																					
Urine Drug Screening[9]	X																					
12-lead ECG[10]	X	X	X	X	X	X	X	X	X	X	X	X	X	X	X	X	X	X	X	X	X	X
Serological testing for infectious diseases [11]	X																					

**Study Phases and Procedures**	**Screening Period [1]**	**Double-Blind Treatment Period [2]**																			**Follow-up**	**Early Withdrawal**
Study Visits	V1	V2	V3	V4	V5	V6	V7	V8	V9	V10	V11	V12	V13	V14	V15	V16	V17	V18	V19	V20	V21	
Study duration (weeks)	-3 ~ 0	1	2	3	4		5		6	7	8		9		10	11	12			13	20	
Study Duration (days)	-21 ~-1	1	8	15	22	23	29	30	36	43	50	51	57	58	64	71	78	79	80	85	134	
Window (days)	-	-	± 2	± 2	± 2	-	± 2	-	± 2	± 2	± 2	-	± 2	-	± 2	± 2	± 2	-	-	± 2	± 3	
Serum calcitonin	X																			X		X
Blood pregnancy test[12]	X						X						X							X		X
Thyroid function [13]	X																					
Glycosylated hemoglobin [14]	X	●																		●		●
OGTT[15]	X																					
Dual energy X-ray absorptiometry (DEXA) [16]		X																		X		X
Abdominal MRI (hepatobiliary pancreatic splenic renal) [16]		X																		X		X
Adverse Events/Concomitant Medications	X	X	X	X	X	X	X	X	X	X	X	X	X	X	X	X	X	X	X	X	X	X
Study drug administration		X	X	X	X		X		X	X	X		X		X	X	X					
PCSK9 Concentration[17]		●																		●		●
FGF21 Concentration[17]		●																		●		●
Immunogenicity [18]		●					●						●							●	●	●

Study Phases and Procedures	Screening Period [1]	Double-Blind Treatment Period [2]																			Follow-up	Early Withdrawal
Study Visits	V1	V2	V3	V4	V5	V6	V7	V8	V9	V10	V11	V12	V13	V14	V15	V16	V17	V18	V19	V20	V21	
Study duration (weeks)	-3 ~ 0	1	2	3	4		5		6	7	8		9		10	11	12			13	20	
Study Duration (days)	-21 ~-1	1	8	15	22	23	29	30	36	43	50	51	57	58	64	71	78	79	80	85	134	
Window (days)	-	-	± 2	± 2	± 2	-	± 2	-	± 2	± 2	± 2	-	± 2	-	± 2	± 2	± 2	-	-	± 2	± 3	
PK/PD Sampling[19]		●	●		●	●	●	●	●		●	●	●	●	●		●	●	●	●		●
Injection site reaction		X	X	X	X		X		X	X	X		X		X	X	X					

Notes:

1. If the examination at screening is more than 5 days away from the day of administration, it should be confirmed again within 5 days before administration, including vital signs, physical examination, hematology, blood biochemistry, lipid profile, coagulation function, urinalysis, myocardial enzymes, serum amylase and lipase, thyroid function, serum calcitonin, ECG and pregnancy test.
2. During the whole treatment period, the dose should be adjusted according to the tolerability of the subjects. Please refer to the dose escalation adjustment table for details.
3. Window for vital signs: to be completed within 1 hour prior to dosing (except for non-dosing visits).
4. Physical examination: in the physical examination of skin and mucosa after administration, attention should be paid to the observation of abnormalities at the injection site. In addition to the examination time points listed in the table, the local skin and mucous membrane at the injection site should be observed for abnormalities immediately (± 2 min), 20 min (± 5 min), 40 min (± 5 min) and 60 min (± 5 min) after administration, including but not limited to skin erythema/redness, swelling, pain/tenderness, congestion/hemorrhage, etc.
5. Body weight: The subjects were required to take off their coat after urination and fasting at each measurement. The same subject used the same scale each time and avoided strenuous exercise before measurement.
6. Waist circumference: (1) standing position, relaxed shoulders and abdomen, smooth breathing, feet 25-30cm apart; (2) The horizontal position of measurement: the midpoint of the line between the anterior superior iliac spine and the inferior margin of the 12th costal line on the midaxillary line; (3) Use a tape ruler around the abdomen in the above-mentioned horizontal position, with the tape ruler closely adhering to the skin, but not strangling the skin; (4) The measurement should not be made consciously with the abdomen closed or lifted, and the measurement should be taken at the calm end of expiration, with the waist circumference in cm to the nearest mm (e.g., 89.3 cm).
7. Hip circumference: (1) The subject stands naturally, with shoulders relaxed, arms naturally drooping and moderately open, legs together, legs evenly loaded, hips relaxed, and eyes ahead; (2) Horizontal positions measured: symphysis pubis anteriorly and greater trochanter of femur posteriorly; (3) Generally equivalent to the most protruding part of the buttock; (4) Wrap the buttocks horizontally with a tape ruler and record the values.
8. 6.2.1Laboratory tests include: (refer to Section 6.2. 1)

1) Hematology: White Blood Cell Count (WBC), Red Blood Cell Count (RBC), Platelet (PLT), Hemoglobin (HGB), Hematocrit (HCT), differential white blood cell count (Absolute Neutrophil (ANC), Basophil Cell (BASO), Eosinophil Count (EOS), Monocyte (MONO) and Lymphocyte (LYM));
2) Blood biochemistry: Aspartate Amino Transferase (AST), Alanine Transaminase (ALT), Total Bilirubin (TBIL), Direct Bilirubin (DBIL), Albumin (ALB), Total Protein (TP), Glutamyl Transpeptidase (GGT), Alkaline Phosphatase (ALP), Lactate Dehydrogenase (LDH), serum potassium, serum sodium, serum calcium, serum chloride, Uric acid (UA), urea (Urea), creatinine (Cr), Fasting Blood Glucos (FBG);
3) Blood lipids: Total cholesterol (TC), Triglyceride (TG), High-density lipoprotein cholesterol (HDL-C), Low-density lipoprotein cholesterol (LDL-C);
4) Coagulation function: Prothrombin Time (PT), Activated Partial Thromboplastin Time (APTT) and International Normalized Ratio (INR);
5) Urinalysis: urine pH, urine protein (URPO), urine glucose (UGLU), urine red blood cells (URBC), urine white blood cells (UWBC);
6) Myocardial enzyme spectrum: creatine kinase (CK), creatine kinase isoenzyme (CK-MB);
7) Blood amylase and blood lipase;

9. Urine drug screen should include: morphine, dimethylenedioxyamphetamine, ketamine, tetrahydrocannabinolic acid, methamphetamine.

10. 212-lead electrocardiogram (ECG) parameters include: RR interval, PR interval, heart rate (HR), QT interval, QTcF (refer to Appendix 3 for calculation formula). Time points of examination: 1W: screening period, within 1h before the first administration, 12h after administration, 24h after administration (D2), 48h after administration (D3), 72h after administration (D4); 2W: within 1h before the second administration; 3W: within 1h before the third administration; 4W: within 1h before the 4th administration and 24h after administration (D23); 5W: within 1h before the 5th administration and 24h after administration (D30); 6W: within 1h before the 6th administration; 7W: within 1h before the 7th administration; 8W: within 1h before and 24h after the 8th administration (D51); 9W: within 1h before the 9th administration and 24h after administration (D58); 10W: within 1h before the 10th administration; 11W: within 1h before the 11th administration; 12W: within 1h before the 12th administration, 24h (D79) and 48h (D80) after administration; 168h after the last dose (D85), at the last follow-up visit (D134). If the subject has abnormal ECG, the investigator can perform additional ECG according to the actual situation. The window period for ECG examination at each visit refers to the window period for PK sampling at the same time point.
11. Serological detection of infectious diseases: including Human Immunodeficiency Virus (HIV) antibody, hepatitis B virus (HBV), hepatitis C virus (HCV) antibody and syphilis antibody.
12. 5.4.2Blood pregnancy test for women of childbearing potential (refer to Section 5.4. 2 for the definition of childbearing potential)
13. Thyroid function indicators include: Thyroid Stimulating Hormone (TSH), free triiodothyronine (Free Triiodothyronine, FT3) and free thyroxine (Free Thyroxine, FT4).
14. Glycosylated hemoglobin test in central laboratory: blood collection points are at V2 visit (D1) and V20 visit (D85) and unscheduled. 2 mL of whole blood will be collected at each blood collection point. Refer to the central laboratory manual for detailed operation procedures.

15. 4For subjects with fasting blood glucose of 6.1-7.0 mmol/L at screening, venous blood glucose 2 hours after OGTT glucose load should be collected for confirmation. Refer to Appendix 5 for OGTT method.
16. Dual-energy X-ray (DEXA) and abdominal MRI are recommended to be performed at sites where conditions permit. DEXA method determination: The subject lies on the examination table, uses the DEXA standard mode, the scanning frame moves from the head side to the foot side, and scans. The scanning conditions were voltage (76 ± 3) KV and current 0.15 mA, tube voltage of two X-rays was 38 V and 70 KV, respectively, the scanning particle range was 197 cmx60 ca, the width was fixed at 60 cm, the scanning time was about 5 min, and the radiation absorber was 0.002 mGy. At the end of the measurement, DEXA device automatically recorded the fat weight of the whole body, waist and buttocks and other parameters, and calculated the waist-buttock fat ratio: waist fat weight (g)/buttock fat weight (g). PLODIGY dual-energy X-ray absorptiometry (Lunar, GE Company, USA) was recommended as the equipment used.
17. PCSK9 and FGF21 test: The blood collection points are V2 visit (D1), V20 visit (D85) and unscheduled, 3 mL of whole blood will be collected at each blood collection point. See the central laboratory manual for detailed operation procedures.
18. Immunogenicity sampling points: Week 1 (within 1h before dosing on Day1), Week 5 (within 1h before dosing on Day29), Week 9 (within 1h before dosing on Day57), Week 13 (168 h ± 12 h after dosing on Day78) and Follow-up Visit on Week 20. If a subject withdraws early, whenever possible, 1 immunogenicity sample will be collected at the early withdrawal visit. 5 mL of whole blood will be collected at each blood collection point. Detailed procedures will be described in the central laboratory manual.
19. **33**Refer to Table 3-1 and Table 3-2 for PK/PD sampling points.
20. In this study, fasting blood collection was required to be fasted for at least 8 hours, and the drug was administered after fasting blood collection and then fed.
21. Eligible subjects should be admitted to the ward before the first dose. During hospitalization, they should have a unified diet and be prohibited from strenuous exercise, coffee and strong tea. They should be hospitalized for at least 3 consecutive days, i.e., complete PK/PD and ECG and other relevant examinations required on D3. They can temporarily leave the hospital after assessment by the investigator. However, subsequent visits should be conducted according to the time specified in the protocol. If conditions permit, it is recommended to be hospitalized before and after the 4th dose, before and after the 5th dose, before and after the 8th dose, before and after the 9th dose, and before and after the 12th dose to complete the examinations required by the visit.
22. ● Central laboratory testing.

3Table 3-1. PK/PD Sampling Schedule (1-5w)-Cohort 1, Cohort 2 and Cohort 3

**Weeks of Dosing**	**1W**									**2W**	**4W**		**5W**			
Dosing Days	D1				D2	D3	D4	D5	D6	D8	D22	D23	D29			D30
Blood sampling time	Within-1h	4h	8h	12h	24h	48h	72h	96 h	120 h	Within-1h	Within-1h	24h	Within-1h	4h	8h	24h
Blood sampling time window	–	± 10 min	± 20 min	± 30 min	± 1 h	± 2 h	± 3 h	± 4 h	± 5 h	_	-	± 1 h	_	± 10 min	± 20 min	± 1 h
Pre-dose	√									√	√		√			
Post-dose		√	√	√	√	√	√	√	√			√		√	√	√
Vital Signs	●	●	●	●	●	●	●	●	●	●	●	●	●	●	●	●
PK sampling	●	●	●	●	●	●	●	●	●	●	●	●	●	●	●	●
Fasting plasma glucose	●					●				●			●			
Fasting insulin/fasting C-peptide	●					●				●			●			
Fasting glucagon	●					●				●			●			
Endogenous GLP-1	●					●				●			●			
Endogenous OXM	●					●				●			●			

Table 3-2. PK/PD Sampling Schedule (6-13w)-Cohort 1, Cohort 2 and Cohort 3

**Weeks of Dosing**	**6W**	**8W**		**9W**				**10W**	**12W**			**13W**
Dosing Days	D36	D50	D51	D57			D58	D64	D78	D79	D80	D85
Blood sampling time	Within-1h	Within-1h	24h	Within-1h	4h	8h	24h	Within-1h	Within-1h	24h	48h	168h after last dose
Blood sampling time window	–		± 1 h	–	± 10 min	± 20 min	± 1 h	–	–	± 1 h	± 2 h	± 12 h
Pre-dose	√	√		√				√	√			
Post-dose			√		√	√	√			√	√	√
Vital Signs	●	●	●	●	●	●	●	●	●	●	●	●
PK sampling	●	●	●	●	●	●	●	●	●	●	●	●
Fasting plasma glucose				●					●		●	●
Fasting insulin/fasting C-peptide				●					●		●	●
Fasting glucagon				●					●		●	●
Endogenous GLP-1				●					●		●	●
Endogenous OXM				●					●		●	●

Notes:

1. PK Sampling Points

Sampling points in Week 1 (intensive PK): within 1 h before dosing, 4 h ± 10 min, 8 h ± 20 min, 12 h ± 30 min, 24 h ± 1 h (Day 2), 48 h ± 2 h (Day 3), 72 h ± 3 h (Day 4), 96 h ± 4 h (Day 5), 120 h ± 5 h (Day 6) after dosing on D1. Sampling in Week 2: within 1h before administration on D8. Sampling in Week 4: within 1h before and 24h ± 1h after administration on D22. Sampling in Week 5: within 1 h before administration and 4 h ± 10 min, 8 h ± 20 min and 24 h ± 1 h after administration on D29. Sampling at Week 6: within 1h before dosing at D36. Sampling in Week 8: within 1h before and 24h ± 1h after D50 administration. Sampling in Week 9: within 1 h before administration and 4 h ± 10 min, 8 h ± 20 min and 24 h ± 1 h after administration on D57. Sampling at Week 10: within 1h before D64 administration. Sampling at Week 12: within 1h before administration and 24h ± 1 h and 48h ± 2 h after administration on D78. Week 13 sampling: 168 h ± 12 h post-dose on D78 (Day 85). If a subject fails to complete the study and prematurely withdraws from the study, PK samples will be collected at the Early Termination Visit. 3mL of whole blood will be collected at each blood collection point. Refer to the central laboratory manual for detailed operation procedures.

2. PD (fasting insulin/fasting C-peptide/fasting plasma glucose/fasting glucagon/OXM/GLP-1) sampling points

Sampling points in Week 1: within 1h before administration (Day 1) and 48 h ± 2 h after administration (Day 3) on D1. Sampling in Week 2: within 1h before administration on D8. Sampling in Week 5: within 1h before administration on D29. Sampling at Week 9: within 1h before dosing on D57. Sampling at Week 12: within 1 h before dosing on D78 and 48 h ± 2 h after dosing on D78 (Day 80). Sampling at Week 13: 168 h ± 12 h post-dose on D78 (Day 85). If a subject fails to complete the study and prematurely withdraws from the study, a PD sample will be collected at the Early Termination Visit. All PD sampling was performed under fasted conditions. A total of 9 mL of whole blood will be collected at each blood collection point. Detailed procedures will be described in the central laboratory manual.

4Table 4. Trial Procedures and Schedule of Assessments (Cohort 4)

**Study Phases and Procedures**	**Screening Period** [1]	**Double-Blind Treatment Period**																											**Safety Follow-up**		**Early Withdrawal**
Study Visits	V1	V2	V3	V4	V5	V6	V7	V8	V9	V10	V11	V12	V13	V14	V15	V16	V17	V18	V19	V20	V21	V22	V23	V24	V25	V26	V27	V28	V29	V30	
Study duration (weeks)	-3 ~ 0	1	2	3	4		5		6	7	8		9		10	11	12			13		14	15	16				17	20	24	
Study Duration (days)	-21 ~-1	1	8	15	22	23	29	30	36	43	50	51	57	58	64	71	78	79	80	85	86	92	99	106	107	108	109	113	134	162	
Window (days)	-	-	±2	±2	±2	-	±2	-	±2	±2	±2	-	±2	-	±2	±2	±2	-	-	±2	-	±2	±2	±2	-	-	-	±2	±3	±3	
Informed Consent	X																														
Inclusion/Exclusion Criteria	X																														
Demographics/Past Medical History/Prior Therapy	X																														
Vital signs[2]	X	X	X	X	X	X	X	X	X	X	X	X	X	X	X	X	X	X	X	X	X	X	X	X	X	X	X	X	X	X	X
Physical Examination [3]	X	X	X	X	X		X		X	X	X		X		X	X	X			X		X	X	X				X	X	X	X
Height	X																														
Weight[4]	X	X	X	X	X	X	X	X	X	X	X	X	X	X	X	X	X	X	X	X	X	X	X	X	X	X	X	X	X	X	X
Waist circumference [5]	X	X					X						X							X								X	X	X	X

Study Phases and Procedures	Screening Period [1]	Double-Blind Treatment Period																											Safety Follow-up		Early Withdrawal
Study Visits	V1	V2	V3	V4	V5	V6	V7	V8	V9	V10	V11	V12	V13	V14	V15	V16	V17	V18	V19	V20	V21	V22	V23	V24	V25	V26	V27	V28	V29	V30	
Study duration (weeks)	-3 ~ 0	1	2	3	4		5		6	7	8		9		10	11	12			13		14	15	16				17	20	24	
Study Duration (days)	-21 ~-1	1	8	15	22	23	29	30	36	43	50	51	57	58	64	71	78	79	80	85	86	92	99	106	107	108	109	113	134	162	
Window (days)	-	-	±2	±2	±2	-	±2	-	±2	±2	±2	-	±2	-	±2	±2	±2	-	-	±2	-	±2	±2	±2	-	-	-	±2	±3	±3	
Hip circumference [6]	X	X					X						X							X								X	X	X	X
Laboratory tests[7]	X						X						X							X								X		X	X
Alcohol breath test	X																														
Urine Drug Screen[8]	X																														
12-lead ECG[9]	X	X	X	X	X	X	X	X	X	X	X	X	X	X	X	X	X	X	X	X	X	X	X	X	X	X	X	X	X	X	X
Serological testing for infectious diseases [10]	X																														
Serum calcitonin	X																											X			X
Blood pregnancy test[11]	X						X						X							X								X			X
Thyroid function[12]	X																											X			

Study Phases and Procedures	Screening Period [1]	Double-Blind Treatment Period																											Safety Follow-up		Early Withdrawal
Study Visits	V1	V2	V3	V4	V5	V6	V7	V8	V9	V10	V11	V12	V13	V14	V15	V16	V17	V18	V19	V20	V21	V22	V23	V24	V25	V26	V27	V28	V29	V30	
Study duration (weeks)	-3 ~ 0	1	2	3	4		5		6	7	8		9		10	11	12			13		14	15	16				17	20	24	
Study Duration (days)	-21 ~-1	1	8	15	22	23	29	30	36	43	50	51	57	58	64	71	78	79	80	85	86	92	99	106	107	108	109	113	134	162	
Window (days)	-	-	±2	±2	±2	-	±2	-	±2	±2	±2	-	±2	-	±2	±2	±2	-	-	±2	-	±2	±2	±2	-	-	-	±2	±3	±3	
Glycosylated haemoglobin [13]	X	●																		●								●			●
OGTT[14]	X																														
Dual energy X-ray absorptiometry (DEXA) [15]		X																										X			X
Abdominal MRI (hepatobiliary pancreatic splenic renal) [15]		X																										X			X
Adverse Events/Concomitant Medications	X	X	X	X	X	X	X	X	X	X	X	X	X	X	X	X	X	X	X	X	X	X	X	X	X	X	X	X	X	X	X

**Study Phases and Procedures**	**Screening Period[1]**	**Double-Blind Treatment Period**																											**Safety Follow-up**		**Early Withdrawal**
Study Visits	V1	V2	V3	V4	V5	V6	V7	V8	V9	V10	V11	V12	V13	V14	V15	V16	V17	V18	V19	V20	V21	V22	V23	V24	V25	V26	V27	V28	V29	V30	
Study duration (weeks)	-3 ~ 0	1	2	3	4		5		6	7	8		9		10	11	12			13		14	15	16				17	20	24	
Study Duration (days)	-21 ~-1	1	8	15	22	23	29	30	36	43	50	51	57	58	64	71	78	79	80	85	86	92	99	106	107	108	109	113	134	162	
Window (days)	-	-	±2	±2	±2	-	±2	-	±2	±2	±2	-	±2	-	±2	±2	±2	-	-	±2	-	±2	±2	±2	-	-	-	±2	±3	±3	
Study drug administration		X	X	X	X		X		X	X	X		X		X	X	X			X		X	X	X							
Immunogenicity[16]		●					●						●							●								●		●	●
PK/PD sampling[17]		●	●		●	●	●	●	●		●	●	●	●	●		●	●	●	●	●	●		●	●	●		●			●
Injection site reaction		X	X	X	X		X		X	X	X		X		X	X	X			X		X	X	X							

Notes:

1. If the examination at screening is more than 5 days away from the day of administration, it should be confirmed again within 5 days before administration, including vital signs, physical examination, hematology, blood biochemistry, lipid profile, coagulation function, urinalysis, myocardial enzymes, serum amylase and lipase, thyroid function, serum calcitonin, ECG and pregnancy test.
2. Window for vital signs: to be completed within 1 hour prior to dosing (except for non-dosing visits).
3. Physical examination: in the physical examination of skin and mucosa after administration, attention should be paid to the observation of abnormalities at the injection site. In addition to the examination time points listed in the table, the local skin and mucous membrane at the injection site should be observed for abnormalities immediately (± 2 min), 20 min (± 5 min), 40 min (± 5 min) and 60 min (± 5 min) after administration, including but not limited to skin erythema/redness, swelling, pain/tenderness, congestion/hemorrhage, etc.
4. Body weight: The subjects were required to take off their coat after urination and fasting at each measurement. The same subject used the same scale each time and avoided strenuous exercise before measurement.
5. Waist circumference: (1) standing position, relaxed shoulders and abdomen, smooth breathing, feet 25-30cm apart; (2) The horizontal position of measurement: the midpoint of the

line between the anterior superior iliac spine and the inferior margin of the 12th costal line on the midaxillary line; (3) Use a tape ruler around the abdomen in the above-mentioned horizontal position, with the tape ruler closely adhering to the skin, but not strangling the skin; (4) The measurement should not be made consciously with the abdomen closed or lifted, and the measurement should be taken at the calm end of expiration, with the waist circumference in cm to the nearest mm (e.g., 89.3 cm).

6. Hip circumference: (1) The subject stands naturally, with shoulders relaxed, arms naturally drooping and moderately open, legs together, legs evenly loaded, hips relaxed, and eyes ahead; (2) Horizontal positions measured: symphysis pubis anteriorly and greater trochanter of femur posteriorly; (3) Generally equivalent to the most protruding part of the buttock; (4) Wrap the buttocks horizontally with a tape ruler and record the values.
7. 6.2.1Laboratory tests include: (refer to Section 6.2. 1)

1) Hematology: white blood cell count (WBC), red blood cell count (RBC), platelets (PLT), hemoglobin (HGB), hematocrit (HCT), differential white blood cell count (neutrophils (ANC), basophils (BASO), eosinophils (EOS), monocytes (MONO) and lymphocytes (LYM));
2) Blood biochemistry: aspartate aminotransferase (AST), alanine aminotransferase (ALT), total bilirubin (TBIL), direct bilirubin (DBIL), albumin (ALB), total protein (TP), glutamyl transpeptidase (GGT), alkaline phosphatase (ALP), lactate dehydrogenase (LDH), serum potassium, serum sodium, serum calcium, serum chloride, uric acid (UA), urea (Urea), creatinine (Cr), fasting serum glucose (FBG);
3) Blood lipids: total cholesterol (TC), triglyceride (TG), high-density lipoprotein cholesterol (HDL-C), low-density lipoprotein cholesterol (LDL-C);
4) Coagulation function: prothrombin time (PT), activated partial thromboplastin time (APTT), international normalized ratio (INR);
5) Urinalysis: urine pH, urine protein (URPO), urine glucose (UGLU), urine red blood cells (URBC), urine white blood cells (UWBC);
6) Myocardial enzyme spectrum: creatine kinase (CK), creatine kinase isoenzyme (CK-MB);
7) Blood amylase, blood lipase.

8. Urine drug screen should include: morphine, dimethylenedioxyamphetamine, ketamine, tetrahydrocannabinolic acid, methamphetamine.

9. 212-lead electrocardiogram (ECG) parameters include: RR interval, PR interval, heart rate (HR), QT interval, QTcF (refer to Appendix 3 for calculation formula). Time points of examination: 1W: screening period, within 1h before the first administration, 12h after administration, 24h after administration (D2), 48h after administration (D3), 72h after administration (D4); 2W: within 1h before the second administration; 3W: within 1h before the third administration; 4W: within 1h before the 4th administration and 24h after administration (D23); 5W: within 1h before the 5th administration and 24h after administration (D30); 6W: within 1h before the 6th administration; 7W: within 1h before the 7th administration; 8W: within 1h before and 24h after the 8th administration (D51); 9W: within 1h before the 9th administration and 24h after administration (D58); 10W: within 1h before the 10th administration; 11W: within 1h before the 11th administration; 12W: within 1h before the 12th administration, 24h (D79) and 48h (D80) after administration; 13W: within 1h before the 13th administration and 24h after administration (D86); 14W: within 1h before the 14th administration; 15W: within 1h before the 15th administration; 16W: within 1h before the 16th administration, 24h after administration (D107), 48h after administration (D108) and 72h after administration (D109, time window ± 2 h); 168h after last dose (D113), safety follow-up (D134, D162). If the subject has abnormal ECG, the investigator can perform additional ECG according to the actual situation. The window period for ECG examination at each visit refers to the window period for PK sampling at the same time point.
10. Serological detection of infectious diseases: including human immunodeficiency virus (HIV) antibody, hepatitis B virus (HBV), hepatitis C (HCV) antibody and syphilis antibody.
11. 5.4.2Blood pregnancy test for women of childbearing potential (refer to Section 5.4. 2 for the definition of childbearing potential)
12. Thyroid function measures include: thyroid stimulating hormone (TSH), free triiodothyronine (FT3), and free thyroxine (FT4).
13. Glycosylated hemoglobin test in central laboratory: blood collection points are V2 visit (D1), V20 visit (D85), V27 visit (D113) and unscheduled visit. 2 mL of whole blood will be collected at each blood collection point. Refer to the central laboratory manual for detailed operation procedures.

14. 4For subjects with fasting blood glucose of 6.1-7.0 mmol/L at screening, venous blood glucose 2 hours after OGTT glucose load should be collected for confirmation. Refer to Appendix 5 for OGTT method.
15. Dual-energy X-ray (DEXA) and abdominal MRI are recommended to be performed at sites where conditions permit. DEXA method determination: The subject lies on the examination table, uses the DEXA standard mode, the scanning frame moves from the head side to the foot side, and scans. The scanning conditions were voltage (76 ± 3) KV and current 0.15 mA, tube voltage of two X-rays was 38 V and 70 KV, respectively, the scanning particle range was 197 cmx60 ca, the width was fixed at 60 cm, the scanning time was about 5 min, and the radiation absorber was 0.002 mGy. At the end of the measurement, DEXA device automatically recorded the fat weight of the whole body, waist and buttocks and other parameters, and calculated the waist-buttock fat ratio: waist fat weight (g)/buttock fat weight (g). PLODIGY dual-energy X-ray absorptiometry (Lunar, GE Company, USA) was recommended as the equipment used.
16. Immunogenicity sampling points: Week 1 (within 1h before dosing on Day1), Week 5 (within 1h before dosing on Day29), Week 9 (within 1h before dosing on Day57), Week 13 (within 1h before dosing on Day85), Week 17 (168 h ± 12 h after dosing on Day106) and Week 24 Follow-up Visit. If a subject withdraws early, whenever possible, 1 immunogenicity sample will be collected at the early withdrawal visit. 5 mL of whole blood will be collected at each blood collection point. Detailed procedures will be described in the central laboratory manual.
17. **55**Refer to Table 5-1 and Table 5-2 for PK/PD sampling points.
18. In this study, fasting blood collection was required to be fasted for at least 8 hours, and the drug was administered after fasting blood collection and then fed.
19. Eligible subjects should be admitted to the ward before the first dose. During hospitalization, they should have a unified diet and be prohibited from strenuous exercise, coffee and strong tea. They should be hospitalized for at least 3 consecutive days, i.e., complete PK/PD and ECG and other relevant examinations required on D3. They can temporarily leave the hospital after assessment by the investigator. However, subsequent visits should be conducted according to the time specified in the protocol. If possible, it is recommended to be hospitalized before and after the 4th dose, before and after the 5th dose, before and after the 8th dose, before and after the 9th dose, before and after the 12th dose, and before and after the 16th dose to complete the examinations required by the visit.
20. ● Central laboratory testing.

5Table 5-1. PK/PD Sampling Schedule (1-8w)--Cohort 4

**Weeks of Dosing**	**1W**						**2W**	**4W**		**5W**		**6W**	**8W**	
Dosing Days	D1	D2	D3	D4	D5	D6	D8	D22	D23	D29	D30	D36	D50	D51

Blood sampling time	Within-1h	4h	8h	12h	24h	48h	72h	96 h	120 h	Within-1h	Within-1h	24h	Within-1h	4h	8h	24h	Within-1h	Within-1h	24h
Blood sampling time window	–	± 10 min	± 20 min	± 30 min	± 1 h	± 2 h	± 3 h	± 4 h	± 5 h	–	-	± 1 h	–	± 10 min	± 20 min	± 1 h	-	–	± 1 h
Pre-dose	√									√	√		√				√	√	
Post-dose		√	√	√	√	√	√	√	√			√		√	√	√			√
Vital Signs	●	●	●	●	●	●	●	●	●	●	●	●	●	●	●	●	●	●	●
PK sampling	●	●	●	●	●	●	●	●	●	●	●	●	●	●	●	●	●	●	●
Fasting plasma glucose	●					●				●			●						
Fasting insulin/fasting C-peptide	●					●				●			●						
Fasting glucagon	●					●				●			●						
Endogenous GLP-1	●					●				●			●						
Endogenous OXM	●					●				●			●						

Table 5-2. PK/PD Sampling Schedule (9-16w)--Cohort 4

**Weeks of Dosing**	**9W**	**10W**	**12W**	**13W**	**14W**	**16W**	**17W**

Dosing Days	D57			D58	D64	D78	D79	D80	D85			D86	D92	D106	D107	D108	D113
Blood sampling time	Within-1h	4h	8h	24h	Within-1h	Within-1h	24h	48h	-1h	4h	8h	24h	Within-1h	Within-1h	24h	48h	168h
Blood sampling time window	_	± 10 min	± 20 min	± 1 h	_	_	± 1 h	± 2 h	_	± 10 min	± 20 min	± 1 h	_	_	± 1 h	± 2 h	± 12 h
Pre-dose	√				√	√			√				√				
Post-dose		√	√	√			√	√		√	√	√			√	√	√
Vital Signs	●	●	●	●	●	●	●	●	●	●	●	●	●	●	●	●	●
PK sampling	●	●	●	●	●	●	●	●	●	●	●	●	●	●	●	●	●
Fasting plasma glucose	●					●		●	●								
Fasting insulin/fasting C-peptide	●					●		●	●								
Fasting glucagon	●					●		●	●								
Endogenous GLP-1	●					●		●	●								
Endogenous OXM	●					●		●	●								

Notes:

1. PK Sampling Points

Sampling points in Week 1 (intensive PK): within 1 h before dosing, 4 h ± 10 min, 8 h ± 20 min, 12 h ± 30 min, 24 h ± 1 h (Day 2), 48 h ± 2 h (Day 3), 72 h ± 3 h (Day 4), 96 h ± 4 h (Day 5), 120 h ± 5 h (Day 6) after dosing on D1. Sampling in Week 2: within 1h before administration on D8. Sampling in Week 4: within

1h before and 24h ± 1h after administration on D22. Sampling in Week 5: within 1 h before administration and 4 h ± 10 min, 8 h ± 20 min and 24 h ± 1 h after administration on D29. Sampling at Week 6: within 1h before dosing at D36. Sampling in Week 8: within 1h before and 24h ± 1h after D50 administration. Sampling in Week 9: within 1 h before administration and 4 h ± 10 min, 8 h ± 20 min and 24 h ± 1 h after administration on D57. Sampling at Week 10: within 1h before D64 administration. Sampling at Week 12: within 1h before administration and 24h ± 1 h and 48h ± 2 h after administration on D78. Sampling in Week 13: within 1 h before administration and 4 h ± 10 min, 8 h ± 20 min and 24 h ± 1 h after administration on D85 (D86). Sampling at Week 14: within 1h before administration at D92. Sampling at Week 16: within 1 h before D106 administration, 24 h ± 1 h and 48 ± 2 h after D106 administration, and 168 h ± 12 h after D106 administration (Day 113). If a subject fails to complete the study and prematurely withdraws from the study, PK samples will be collected at the Early Termination Visit. 3mL of whole blood will be collected at each blood collection point. Refer to the central laboratory manual for detailed operation procedures.

2. PD (fasting insulin/fasting C-peptide/fasting plasma glucose/fasting glucagon/OXM/GLP-1) sampling points

Sampling points in Week 1: within 1h before administration (Day 1) and 48 h ± 2 h after administration (Day 3) on D1. Sampling in Week 2: within 1h before administration on D8. Sampling in Week 5: within 1h before administration on D29. Sampling at Week 9: within 1h before dosing on D57. Sampling at Week 12: within 1 h before dosing on D78 and 48 h ± 2 h after dosing on D78 (Day 80). Sampling at Week 13: within 1h before D85 administration. If a subject fails to complete the study and prematurely withdraws from the study, a PD sample will be collected at the Early Termination Visit. All PD sampling was performed under fasted conditions. A total of 9 mL of whole blood will be collected at each blood collection point. Detailed procedures will be described in the central laboratory manual.

6Table 6. Trial Procedures and Schedule of Assessments (Cohort 5)

**Study Phases and Procedures**	**Screening Period** [1]	**Double-Blind Treatment Period**																				**Safety Follow-up**		**Early Withdrawal**
Study Visits	V1	V2	V3	V4	V5	V6	V7	V8	V9	V10	V11	V12	V13	V14	V15	V16	V17	V18	V19	V20	V21	V22	V23	
Study duration (weeks)	-3 ~ 0	1	2	3	4		5		6	7	8		9		10	11	12				13	16	20	
Study Duration (days)	-21 ~-1	1	8	15	22	23	29	30	36	43	50	51	57	58	64	71	78	79	80	81	85	106	134	
Window (days)	-	-	±2	±2	±2	-	±2	-	±2	±2	±2	-	±2	-	±2	±2	±2	-	-	-	±2	±3	±3	
Informed Consent	X																							
Inclusion/Exclusion Criteria	X																							
Demographics/Past Medical History/Prior Therapy	X																							
Vital signs[2]	X	X	X	X	X	X	X	X	X	X	X	X	X	X	X	X	X	X	X	X	X	X	X	X
Physical Examination[3]	X	X	X	X	X		X		X	X	X		X		X	X	X				X	X	X	X
Height	X																							
Weight[4]	X	X	X	X	X	X	X	X	X	X	X	X	X	X	X	X	X	X	X	X	X	X	X	X
Waist circumference [5]	X	X					X						X								X	X	X	X
Hip circumference [6]	X	X					X						X								X	X	X	X
Laboratory tests[7]	X						X						X								X		X	X
Alcohol breath test	X																							
Urine Drug Screen[8]	X																							

Study Phases and Procedures	Screening Period[1]	Double-Blind Treatment Period																				Safety Follow-up		Early Withdrawal
Study Visits	V1	V2	V3	V4	V5	V6	V7	V8	V9	V10	V11	V12	V13	V14	V15	V16	V17	V18	V19	V20	V21	V22	V23	
Study duration (weeks)	-3 ~ 0	1	2	3	4		5		6	7	8		9		10	11	12				13	16	20	
Study Duration (days)	-21 ~-1	1	8	15	22	23	29	30	36	43	50	51	57	58	64	71	78	79	80	81	85	106	134	
Window (days)	-	-	±2	±2	±2	-	±2	-	±2	±2	±2	-	±2	-	±2	±2	±2	-	-	-	±2	±3	±3	
12-lead ECG[9]	X	X	X	X	X	X	X	X	X	X	X	X	X	X	X	X	X	X	X	X	X	X	X	X
Serological testing for infectious diseases [10]	X																							
Serum calcitonin	X																				X			X
Blood pregnancy test[11]	X						X						X								X			X
Thyroid function[12]	X																				X			
Glycosylated haemoglobin [13]	X	●																			●			●
OGTT[14]	X																							
Dual energy X-ray absorptiometry (DEXA) [15]		X																			X			X
Abdominal MRI (hepatobiliary pancreatic splenic renal) [15]		X																			X			X
Adverse Events/Concomitant Medications	X	X	X	X	X	X	X	X	X	X	X	X	X	X	X	X	X	X	X	X	X	X	X	X

**Study Phases and Procedures**	**Screening Period** [1]	**Double-Blind Treatment Period**																				**Safety Follow-up**		**Early Withdrawal**
Study Visits	V1	V2	V3	V4	V5	V6	V7	V8	V9	V10	V11	V12	V13	V14	V15	V16	V17	V18	V19	V20	V21	V22	V23	
Study duration (weeks)	-3 ~ 0	1	2	3	4		5		6	7	8		9		10	11	12				13	16	20	
Study Duration (days)	-21 ~-1	1	8	15	22	23	29	30	36	43	50	51	57	58	64	71	78	79	80	81	85	106	134	
Window (days)	-	-	±2	±2	±2	-	±2	-	±2	±2	±2	-	±2	-	±2	±2	±2	-	-	-	±2	±3	±3	
Study drug administration		X	X	X	X		X		X	X	X		X		X	X	X							
Immunogenicity [16]		●					●						●								●		●	●
PK/PD sampling[17]		●	●		●	●	●	●	●		●	●	●	●	●		●	●	●		●			●
Injection site reaction		X	X	X	X		X		X	X	X		X		X	X	X							

Notes:

1. If the examination at screening is more than 5 days away from the day of administration, it should be confirmed again within 5 days before administration, including vital signs, physical examination, hematology, blood biochemistry, lipid profile, coagulation function, urinalysis, myocardial enzymes, serum amylase and lipase, thyroid function, serum calcitonin, ECG and pregnancy test.
2. Window for vital signs: to be completed within 1 hour prior to dosing (except for non-dosing visits).
3. Physical examination: in the physical examination of skin and mucosa after administration, attention should be paid to the observation of abnormalities at the injection site. In addition to the examination time points listed in the table, the local skin and mucous membrane at the injection site should be observed for abnormalities immediately (± 2 min), 20 min (± 5 min), 40 min (± 5 min) and 60 min (± 5 min) after administration, including but not limited to skin erythema/redness, swelling, pain/tenderness, congestion/hemorrhage, etc.
4. Body weight: The subjects were required to take off their coat after urination and fasting at each measurement. The same subject used the same scale each time and avoided strenuous exercise before measurement.
5. Waist circumference: (1) standing position, relaxed shoulders and abdomen, smooth breathing, feet 25-30cm apart; (2) The horizontal position of measurement: the midpoint of the line between the anterior superior iliac spine and the inferior margin of the 12th costal line on the midaxillary line; (3) Use a tape ruler around the abdomen in the above-mentioned horizontal position, with the tape ruler closely adhering to the skin, but not strangling the skin; (4) The measurement should not be made consciously with the abdomen closed or lifted, and the measurement should be taken at the calm end of expiration, with the waist circumference in cm to the nearest mm (e.g., 89.3 cm).

6. Hip circumference: (1) The subject stands naturally, with shoulders relaxed, arms naturally drooping and moderately open, legs together, legs evenly loaded, hips relaxed, and eyes ahead; (2) Horizontal positions measured: symphysis pubis anteriorly and greater trochanter of femur posteriorly; (3) Generally equivalent to the most protruding part of the buttock; (4) Wrap the buttocks horizontally with a tape ruler and record the values.

7. 6.2.1Laboratory tests include: (refer to Section 6.2. 1)

1) Hematology: white blood cell count (WBC), red blood cell count (RBC), platelets (PLT), hemoglobin (HGB), hematocrit (HCT), differential white blood cell count (neutrophils (ANC), basophils (BASO), eosinophils (EOS), monocytes (MONO) and lymphocytes (LYM));
2) Blood biochemistry: aspartate aminotransferase (AST), alanine aminotransferase (ALT), total bilirubin (TBIL), direct bilirubin (DBIL), albumin (ALB), total protein (TP), glutamyl transpeptidase (GGT), alkaline phosphatase (ALP), lactate dehydrogenase (LDH), serum potassium, serum sodium, serum calcium, serum chloride, uric acid (UA), urea (Urea), creatinine (Cr), fasting serum glucose (FBG);
3) Blood lipids: total cholesterol (TC), triglyceride (TG), high-density lipoprotein cholesterol (HDL-C), low-density lipoprotein cholesterol (LDL-C);
4) Coagulation function: prothrombin time (PT), activated partial thromboplastin time (APTT), international normalized ratio (INR);
5) Urinalysis: urine pH, urine protein (URPO), urine glucose (UGLU), urine red blood cells (URBC), urine white blood cells (UWBC);
6) Myocardial enzyme spectrum: creatine kinase (CK), creatine kinase isoenzyme (CK-MB);
7) Blood amylase, blood lipase.

8. Urine drug screen should include: morphine, dimethylenedioxyamphetamine, ketamine, tetrahydrocannabinolic acid, methamphetamine.

9. 212-lead electrocardiogram (ECG) parameters include: RR interval, PR interval, heart rate (HR), QT interval, QTcF (refer to Appendix 3 for calculation formula). Time points of examination: 1W: screening period, within 1h before the first administration, 12h after administration, 24h after administration (D2), 48h after administration (D3), 72h after administration (D4); 2W: within 1h before the second administration; 3W: within 1h before the third administration; 4W: within 1h before the 4th administration and 24h after administration (D23); 5W: within 1h before the 5th administration and 24h after administration (D30); 6W: within 1h before the 6th administration; 7W: within 1h before the 7th administration; 8W: within 1h before and 24h after the 8th administration (D51); 9W: within 1h before the 9th administration and 24h after administration (D58); 10W: within 1h before the 10th administration; 11W: within 1h before the 11th administration; 12W: within 1h before the 12th administration, 24h after administration (D79), 48h after administration (D80) and 72h after administration (D81, time window ± 2 h); 168h after the last dose (D85), safety follow-up (D106, D134). If the subject has abnormal ECG, the investigator can perform additional ECG according to the actual situation. The window period for ECG examination at each visit refers to the window period for PK sampling at the same time point.
10. Serological detection of infectious diseases: including human immunodeficiency virus (HIV) antibody, hepatitis B virus (HBV), hepatitis C (HCV) antibody and syphilis antibody.
11. 5.4.2Blood pregnancy test for women of childbearing potential (refer to Section 5.4. 2 for the definition of childbearing potential)
12. Thyroid function measures include: thyroid stimulating hormone (TSH), free triiodothyronine (FT3), and free thyroxine (FT4).
13. Glycosylated hemoglobin test in central laboratory: blood collection points are at V2 visit (D1) and V20 visit (D85) and unscheduled. 2 mL of whole blood will be collected at each blood collection point. Refer to the central laboratory manual for detailed operation procedures.

14. 4For subjects with fasting blood glucose of 6.1-7.0 mmol/L at screening, venous blood glucose 2 hours after OGTT glucose load should be collected for confirmation. Refer to Appendix 5 for OGTT method.
15. Dual-energy X-ray (DEXA) and abdominal MRI are recommended to be performed at sites where conditions permit. DEXA method determination: The subject lies on the examination table, uses the DEXA standard mode, the scanning frame moves from the head side to the foot side, and scans. The scanning conditions were voltage (76 ± 3) KV and current 0.15 mA, tube voltage of two X-rays was 38 V and 70 KV, respectively, the scanning particle range was 197 cmx60 ca, the width was fixed at 60 cm, the scanning time was about 5 min, and the radiation absorber was 0.002 mGy. At the end of the measurement, DEXA device automatically recorded the fat weight of the whole body, waist and buttocks and other parameters, and calculated the waist-buttock fat ratio: waist fat weight (g)/buttock fat weight (g). PLODIGY dual-energy X-ray absorptiometry (Lunar, GE Company, USA) was recommended as the equipment used.
16. Immunogenicity sampling points: Week 1 (within 1h before dosing on Day1), Week 5 (within 1h before dosing on Day29), Week 9 (within 1h before dosing on Day57), Week 13 (168 h ± 12 h after dosing on Day78) and Follow-up Visit on Week 20. If a subject withdraws early, whenever possible, 1 immunogenicity sample will be collected at the early withdrawal visit. 5 mL of whole blood will be collected at each blood collection point. Detailed procedures will be described in the central laboratory manual.
17. **77**Refer to Table 7-1 and Table 7-2 for PK/PD sampling points.
18. In this study, fasting blood collection was required to be fasted for at least 8 hours, and the drug was administered after fasting blood collection and then fed.
19. Eligible subjects should be admitted to the ward before the first dose. During hospitalization, they should have a unified diet and be prohibited from strenuous exercise, coffee and strong tea. They should be hospitalized for at least 3 consecutive days, i.e., complete PK/PD and ECG and other relevant examinations required on D3. They can temporarily leave the hospital after assessment by the investigator. However, subsequent visits should be conducted according to the time specified in the protocol. If conditions permit, it is recommended to be hospitalized before and after the 4th dose, before and after the 5th dose, before and after the 8th dose, before and after the 9th dose, and before and after the 12th dose to complete the examinations required by the visit.
20. ● Central laboratory testing.

7Table 7-1. PK/PD Sampling Schedule (1-5w)--Cohort 5

**Weeks of Dosing**	**1W**									**2W**	**4W**		**5W**			
Dosing Days	D1				D2	D3	D4	D5	D6	D8	D22	D23	D29			D30
Blood sampling time	Within-1h	4h	8h	12h	24h	48h	72h	96 h	120 h	Within-1h	Within-1h	24h	Within-1h	4h	8h	24h
Blood sampling time window	–	± 10 min	± 20 min	± 30 min	± 1 h	± 2 h	± 3 h	± 4 h	± 5 h	_	-	± 1 h	_	± 10 min	± 20 min	± 1 h
Pre-dose	√									√	√		√			
Post-dose		√	√	√	√	√	√	√	√			√		√	√	√
Vital Signs	●	●	●	●	●	●	●	●	●	●	●	●	●	●	●	●
PK sampling	●	●	●	●	●	●	●	●	●	●	●	●	●	●	●	●
Fasting plasma glucose	●					●				●			●			
Fasting insulin/fasting C-peptide	●					●				●			●			
Fasting glucagon	●					●				●			●			
Endogenous GLP-1	●					●				●			●			
Endogenous OXM	●					●				●			●			

Table 7-2. PK/PD Sampling Schedule (6-13w)--Cohort 5

**Weeks of Dosing**	**6W**	**8W**		**9W**				**10W**	**12W**			**13W**
Dosing Days	D36	D50	D51	D57			D58	D64	D78	D79	D80	D85
Blood sampling time	Within-1h	Within-1h	24h	Within-1h	4h	8h	24h	Within-1h	Within-1h	24h	48h	168h after last dose
Blood sampling time window	–		± 1 h	–	± 10 min	± 20 min	± 1 h	–	–	± 1 h	± 2 h	± 12 h
Pre-dose	√	√		√				√	√			
Post-dose			√		√	√	√			√	√	√
Vital Signs	●	●	●	●	●	●	●	●	●	●	●	●
PK sampling	●	●	●	●	●	●	●	●	●	●	●	●
Fasting plasma glucose				●					●		●	●
Fasting insulin/fasting C-peptide				●					●		●	●
Fasting glucagon				●					●		●	●
Endogenous GLP-1				●					●		●	●
Endogenous OXM				●					●		●	●

Notes:

1. PK Sampling Points

Sampling points in Week 1 (intensive PK): within 1 h before dosing, 4 h ± 10 min, 8 h ± 20 min, 12 h ± 30 min, 24 h ± 1 h (Day 2), 48 h ± 2 h (Day 3), 72 h ± 3 h (Day 4), 96 h ± 4 h (Day 5), 120 h ± 5 h (Day 6) after dosing on D1. Sampling in Week 2: within 1h before administration on D8. Sampling in Week 4: within 1h before and 24h ± 1h after administration on D22. Sampling in Week 5: within 1 h before administration and 4 h ± 10 min, 8 h ± 20 min and 24 h ± 1 h after administration on D29. Sampling at Week 6: within 1h before dosing at D36. Sampling in Week 8: within 1h before and 24h ± 1h after D50 administration. Sampling in Week 9: within 1 h before administration and 4 h ± 10 min, 8 h ± 20 min and 24 h ± 1 h after administration on D57. Sampling at Week 10: within

1h before D64 administration. Sampling at Week 12: within 1h before administration and 24h ± 1 h and 48h ± 2 h after administration on D78. Week 13 sampling: 168 h ± 12 h post-dose on D78 (Day 85). If a subject fails to complete the study and prematurely withdraws from the study, PK samples will be collected at the Early Termination Visit. 3mL of whole blood will be collected at each blood collection point. Refer to the central laboratory manual for detailed operation procedures.

2. PD (fasting insulin/fasting C-peptide/fasting plasma glucose/fasting glucagon/OXM/GLP-1) sampling points

Sampling points in Week 1: within 1h before administration (Day 1) and 48 h ± 2 h after administration (Day 3) on D1. Sampling in Week 2: within 1h before administration on D8. Sampling in Week 5: within 1h before administration on D29. Sampling at Week 9: within 1h before dosing on D57. Sampling at Week 12: within 1 h before dosing on D78 and 48 h ± 2 h after dosing on D78 (Day 80). Week 13 sampling: 168 h ± 12 h post-dose on D78 (Day 85). If a subject fails to complete the study and prematurely withdraws from the study, a PD sample will be collected at the Early Termination Visit. All PD sampling was performed under fasted conditions. A total of 9 mL of whole blood will be collected at each blood collection point. Detailed procedures will be described in the central laboratory manual.

Table of Contents

Investigator Signature Page 2
Sponsor Signature Page 3
Protocol Summary 4
Table of Contents 45
Table of Tables 49
List of Figures 49
List of Abbreviations and Definitions of Terms 50
1 Introduction 52
1.1 Study Background 52
1.1.1 GLP-1R/GCGR Dual Agonists 52
1.1.2 Obesity and overweight 53
1.2 Study Rationale 54
1.3 Potential Risks and Benefits of Treatment Options 55
1.3.1 Potential Risks for IBI362 Identified Based on Preclinical Safety Studies .. 55
1.3.2 Potential Risks for IBI362 Based on Clinical Safety Studies 56
1.3.3 Potential Benefits of IBI362 Based on Clinical Safety Studies 62
2 Study Objectives and Endpoints 63
2.1 Study Objectives 63
2.1.1 Primary Objective 63
2.1.2 Secondary Objectives 64
2.2 Study Endpoints 64
2.2.1 Primary Endpoint 64
2.2.2 Secondary Endpoints 64
2.2.3 Exploratory Endpoints 64
3 Overall Study Design 65
3.1 Design Rationale 65
3.1.1 Dose Selection Rationale 65
3.2 Phase Ib/II Study Design 66
3.2.1 Overall Study Design 66
3.2.2 Actions Taken to Avoid Bias 68
3.2.3 Definition of End of Study 68
4 Study Population 68
4.1 Inclusion Criteria 69
4.2 Exclusion Criteria 69

4.3 Restrictions during the Study 72
4.4 Subject Screening 72
4.4.1 Enrollment Procedure 72
4.4.2 Handling Procedures for Incorrectly Enrolled Subjects 73
4.4.3 Randomization and Blinding 73
4.5 Subject Replacement, Discontinuation, and Withdrawal Criteria 73
4.5.1 Subject Replacement 73
4.5.2 Discontinued medication 73
4.5.3 Withdrawal Criteria 74
4.5.4 Exit Procedure 75
4.6 Subject Withdrawal from the Study 75
5 Study Drug and Other Treatments 75
5.1 Treatment Assignment 75
5.1.1 Subject Number and Treatment Assignment 75
5.1.2 Randomization and Blinding 75
5.2 Study drug 76
5.2.1 Physical and chemical characteristics of the study drug 76
5.2.2 Study Drug Strength and Manufacturer 76
5.2.3 Storage 76
5.2.4 Mode of administration 76
5.3 Concomitant Therapy 76
5.3.1 Prohibited Medications 76
5.4 Dosing during pregnancy, childbearing potential, or lactation 77
5.4.1 Pregnancy 77
5.4.2 Childbearing age 77
5.4.3 Lactation 78
5.5 Treatment compliance 78
5.6 Drug Recovery and Destruction 78
5.7 Records of Study Drug 78
5.8 Complaint Handling 79
6 Study Assessments 79
6.1 Safety and Tolerability Assessments 79
6.2 Safety and Other Assessments 79
6.2.1 Laboratory Tests 79
6.2.2 Physical examination 80

6.2.3 Vital Signs........81
6.2.4 ECG........81
6.2.5 Fasting body weight measurement........82
6.2.6 Pregnancy test........82
6.3 PK/PD assessment variables........83
6.3.1 PK Sample Collection........83
6.3.2 Pharmacodynamic Sample Collection........83
6.3.3 PK/PD variables........83
6.4 Immunogenicity evaluation indicators........83
6.4.1 Immunogenicity blood collection points........83
6.4.2 Immunogenicity indicators........83
7 Safety Reporting and Adverse Event Management........83
7.1 Definition of Adverse Events........83
7.2 Definition of Serious Adverse Events........84
7.3 Severity assessment of adverse events........85
7.4 Causal relationship judgment between adverse event and investigational drug..85
7.5 Recording of Adverse Events........86
7.5.1 Adverse event collection and time interval........86
7.5.2 Follow-up of adverse events........86
7.5.3 Contents of Adverse Event Records........87
7.6 Expedited Reporting of SAE and Pregnancy........88
7.7 Adverse Events of Special Interest (AESI) and Reporting Process........89
7.7. 1 Hepatic Function Abnormal Events........89
7.7. 2 Hypoglycemic Events........90
7.7. 3 Gastrointestinal Reactions........92
7.7. 4 Acute pancreatitis........92
8 Data Analysis/Statistical Methods........92
8.1 Statistical Hypothesis........92
8.2 Sample Size Estimation........92
8.3 Statistical Analysis Populations........92
8.4 Statistical Analysis........93
8.4. 1 General Methods........93
8.4. 2 Efficacy Analysis........93
8.4. 3 Safety Analysis........93
8.4. 4 Immunogenicity........94

8.4. 5 Analysis of Pharmacokinetic and Pharmacodynamic Parameters.................94
8.4. 6 Exploratory Analyses...95
8.5 Comparison of multiplicity..95
8.6 Control of bias 95
8.6.1 Randomization and blinding..95
8.6.2 Assessment of Blinding Maintenance 95
8.6.3 Unblinding and Emergency Unblinding..95
9 Quality Assurance and Quality Control...96
9.1 Clinical Monitoring 96
9.2 Data Management/Coding..97
9.3 Quality Assurance Audit...98
10 Ethics 98
10.1 Ethics Committee...98
10.2 Ethical Conduct in the Study 99
10.3 Subject Information and Informed Consent 99
10.4 Data Protection 100
10.5 Protocol Violation..100
11 Study Management...100
11.1 Data Handling and Record Retention..100
11.2 Access to Raw Data/Documents..100
11.3 Protocol Amendment 101
11.4 Investigator Responsibilities..101
11.5 Publication Policy..101
11.6 Finance and Insurance 102
12 Protocol Amendment History...103
13 References 104
14 Appendix 106
Appendix 1: Pancreatic Enzyme Monitoring and Management Procedures for Patients without Symptoms of Pancreatitis...106
1Appendix 2: CKD-EPI formula (eGFR estimation formula) 107
2Appendix 3: QTcF Calculation Formula 108
3Appendix 4: Dyslipidemia Reference Standards...109
4Appendix 5: Oral Glucose Tolerance Test (OGTT) Methods......................................110
5Appendix 6: AE Reporting Process Related to Dose Escalation Discontinuation 111

Table of Tables

TABLE 1. REFERENCE TABLE FOR DOSE AND ADJUSTMENT DURING ESCALATION (COHORT 1, COHORT 2 AND COHORT 3) 16

TABLE 2. TRIAL PROCEDURES AND SCHEDULE OF ASSESSMENTS (COHORT 1, COHORT 2, AND COHORT 3) 17

TABLE 3-1. PK/PD SAMPLING SCHEDULE (1-5W)-COHORT 1, COHORT 2 AND COHORT 3 23

TABLE 4. TRIAL PROCEDURES AND SCHEDULE OF ASSESSMENTS (COHORT 4) 26

TABLE 5-1. PK/PD SAMPLING SCHEDULE (1-8W)--COHORT 4 32

TABLE 6. TRIAL PROCEDURES AND SCHEDULE OF ASSESSMENTS (COHORT 5) 36

TABLE 7-1. PK/PD SAMPLING SCHEDULE (1-5W)--COHORT 5 42

TABLE 8. SUMMARY OF ADVERSE EVENTS IN SUBJECTS WITH SINGLE DOSE ESCALATION IN I8P-MC-OXAA STUDY TEAES 56

TABLE 9. SUMMARY OF ADVERSE EVENTS IN SUBJECTS WITH MULTIPLE DOSE ESCALATION IN I8P-MC-OXAB STUDY TEAES 57

TABLE 10. SUMMARY OF TREATMENT-EMERGENT ADVERSE EVENTS (TEAE) BY SYSTEM ORGAN CLASS AND PREFERRED TERM COHORT 1 59

TABLE 11. SUMMARY OF TREATMENT-EMERGENT ADVERSE EVENTS (TEAE) BY SYSTEM ORGAN CLASS AND PREFERRED TERM COHORT 2 60

TABLE 12. SUMMARY OF TREATMENT-EMERGENT ADVERSE EVENTS (TEAE) BY SYSTEM ORGAN CLASS AND PREFERRED TERM COHORT 3 61

TABLE 13. LIVER ENZYME ELEVATIONS AT BASELINE AND DURING THE TREATMENT PERIOD 73

TABLE 14. ROUTINE LABORATORY SAFETY ASSESSMENTS 80

TABLE 15. HEPATIC IMPAIRMENT REQUIRING REPORTING AS AN SAE 90

List of Figures

FIGURE 1. STUDY TRIAL DESIGN DIAGRAM (COHORT 1, COHORT 2, AND COHORT 3) 14

FIGURE 2. STUDY DESIGN DIAGRAM (COHORT 4 AND COHORT 5) 15

FIGURE 3. STUDY I8P-MC-OXAB: CHANGE FROM BASELINE IN BODY WEIGHT 62

FIGURE 4. STUDY CIBI362B101: CHANGE FROM BASELINE IN BODY WEIGHT 63

FIGURE 5. STUDY CIBI362B101: CHANGE FROM BASELINE IN BODY WEIGHT 63

List of Abbreviations and Definitions of Terms

Abbreviations	Full name
ADA	Anti-drug Antibody
AE	Adverse Event
AESI	Adverse Event of Special Interest
ALB	Albumin
ALP	Alkaline Phosphatase
ALT	Alanine Transaminase
ANC	Absolute Neutrophil
APTT	Activated Partial Thromboplastin Time
AR	Accumulation Ratio
AST	Aspartate Amino Transferase
AUC	Area Under Curve
BMI	Body Mass Index
BASO	Basophil Cell
CK	Creatine Kinase
CL	Clearance
Cmax	Maximum Serum Concentration of Drug
Cr	Creatinine
CRA	Clinical Research Associate
CRO	Contract Research Organization
CSR	Clinical Study Report
DBIL	Direct Bilirubin
EC	Ethics Committee
ECG	Electrocardiograms
EDC	Electronic Data Capture
ECRF	Electronic Case Report Form
EOS	Eosinophil
FBG	Fasting Blood Glucos
FT3	Free Triiodothyronine
FT4	Free Thyroxine
GGT	Glutamyl Transpeptidase
GLP-1	Glucagon Like Peptide 1
HCT	Hematocrit
HCV	Hepatitis C Virus
HDL-C	High-Density Lipoprotein Cholesterol
HGB	Hemoglobin
HIV	Human Immunodeficiency Virus

Abbreviations	Full name
HR	Heart Rate
ICF	Informed Consent Form
INR	International Normalized Ratio
LDH	Lactate Dehydrogenase
LDL-C	Low-Density Lipoprotein Cholesterol
LYM	Lymphocyte
MedDRA	Medical Dictionary for Regulatory Activities
MTD	Maximal Tolerated Dose
MONO	Monocyte
NAb	Neutralizing Antibody
NOAEL	No Observed Adverse Effect Level
OXM	Oxyntomodulin
PLT	Platelet
PT	Prothrombin Time
RBC	Red Blood Cell Count
TBIL	Total Bilirubin
TC	Total Cholesterol
TEAE	Treatment Emergent Adverse Event
TG	Triglyceride
TP	Total Protein
TSH	Thyroid Stimulating Hormone
UA	Uric Acid
UGLU	URINARY GLUCOSE
URBC	Urinary Red Blood Cells
Urea	Urea
URPO	Urine Protein
UWBC	Urine Protein
Vd	Distribution Volume
WBC	White Blood Cell Count

1 Introduction

1.1 Study Background

IBI362 is a long-acting synthetic peptide similar to the mammalian oxyntomodulin (OXM) that utilizes a fatty acyl side chain to prolong the duration of action, allowing for once-weekly administration. OXM can improve glucose tolerance and cause weight loss when administered exogenously [1]. In humans, this hormone is thought to exert its biological effects by activating the glucagon-like peptide-1 receptor (GLP-1R) and glucagon receptor (GCGR) [2]. As an OXM analog, the effects of IBI362 are thought to be mediated through binding and activation of GLP-1R and GCGR. In addition to GLP-1R agonists having insulin secretion-promoting and blood glucose-lowering effects, IBI362 is currently found to have a weight-reducing effect, possibly through its effects on GCGR.

The results of a single multiple ascending dose study of IBI362 demonstrated a favorable safety and tolerability profile, as well as a significant weight loss effect, and the Sponsor proposes to develop IBI362 for the treatment of overweight or obese subjects in a weight loss indication.

1.1.1 GLP-1R/GCGR Dual Agonists

Glucagon Like Peptide 1 (GLP-1) is a peptide hormone secreted from the gut that has multiple mechanisms aimed at lowering blood glucose and weight loss, including increased glucose-dependent insulin secretion, suppression of glucagon secretion, delay of gastric emptying, and central appetite suppression.

Glucagon, a hormone secreted by pancreatic islet α cells, consists of a single-chain polypeptide 29 amino acids in length. Glucagon exerts its physiological effects by specifically binding to the glucagon receptor (GCGR) on the surface of target cells in liver and liver and kidney, activating the intracellular adenylate cyclase (Adenylate Cyclase) and increasing the intracellular cAMP level. Glucagon is a catabolic hormone, short-term injection of glucagon can promote glycogenolysis and gluconeogenesis, so that blood glucose increases. However, studies have found that long-term activation of GCGR by glucagon injection can reduce appetite, stimulate fatty acid breakdown, and significantly increase energy expenditure in adipose tissue [3].

Oxyntomodulin (OXM) is a peptide hormone secreted by human intestinal L cells after nutrient ingestion. OXM, a dual agonist of the glucagon-like peptide-1 receptor (GLP1R) and glucagon receptor (GCGR), combines the actions of GLP-1 and glucagon and may be more effective than GLP1R agonists for the treatment of obesity. Simultaneous injection of OXM in humans significantly decreased body weight and appetite and increased energy expenditure.

1.1.2 Obesity and overweight

Obesity refers to excessive accumulation and (or) abnormal distribution of body fat, usually accompanied by weight gain. "China Adult Obesity Prevention Expert Consensus" pointed out [4], in 2005 WHO work report estimated that around 1.6 billion adults (15 years of age or older) are overweight, obesity adults at least 400 million; At least 20 million children under the age of 5 are obese. The 2014 WHO Global Burden of Disease (GDB) study showed that: since 1980, the proportion of obese/overweight adults in the world has increased by 28%, and that of children has increased by 47%. The total number of overweight and obese people in the world has increased from 857 million in 1980 to 2.1 billion in 2013, with the United States ranking first, followed by China [5]. According to the latest data from the CDC: The prevalence of adult obesity was 42.4% in 2017-2018, with 9.2% of severe obesity; The prevalence of obesity in children and minors (aged 2-19 years) is 18.5%, about 13.7 million [6]. As many as 300,000 Americans die from obesity-related diseases every year, and the direct economic losses caused by obesity and inactivity account for about 9.4% of health care expenditure in the United States [7]. China is also facing the obesity pandemic problem. From 1992 to 2015, the overweight rate increased from 13% to 30%, and the obesity rate increased from 3% to 12%. Meanwhile, from 2002 to 2015, the overweight rate of children and adolescents increased from 4.5% to 9.6%, and the obesity rate increased from 2.1% to 6.4% [8]. China has surpassed the United States to become the world's most obese country, according to the 2015 Global Adult Weight Survey. Among them, the number of male obesity in China is 43.2 million, the number of female obesity is 46.4 million, and the number of overweight is nearly 400 million, and the prevalence rate shows a gradually increasing trend [8].

[4][9][10]Obesity can lead to a number of complications or related diseases, which can affect life expectancy or lead to reduced quality of life. In the more severe obese subjects, cardiovascular disease, diabetes and some tumors incidence and mortality significantly increased. The Chinese Obesity Working Group of the China Office of the International Society of Life Sciences summarized and analyzed the relationship between BMI and the prevalence of related diseases based on the large-scale measurement data of Chinese population. The results showed that the risk of hypertension in people with BMI ≥ 24 kg/m^2 was 3-4 times higher than that in people with normal weight (BMI 18.5-23.9 kg/m^2), and the risk of diabetes was 2-3 times higher than that in people with normal weight [4]. Even 5-10% weight loss reduces the risk of developing type 2 diabetes, hypertension, dyslipidemia, and obstructive sleep apnea [9] [10].

Lifestyle intervention is the most important first choice and basic treatment for overweight or obesity, but there are still a considerable number of subjects still can not lose weight for various reasons, or can not achieve the desired weight loss goal, drug-assisted weight loss can be considered. At present, foreign guidelines stipulate that for obese subjects with body mass index (BMI) ≥ 30 kg/m^2 or BMI ≥ 27 kg/m^2 accompanied

by at least one obesity concomitant disease, such as diabetes, hypertension, dyslipidemia, obstructive sleep apnea syndrome, etc., weight loss drugs can be considered. Our guidelines suggest that, on the premise of adequate diet, exercise and behavioral therapy, drug therapy can be taken for those with the following conditions: having a strong appetite and eating a large amount of meals; Complicated with hyperglycemia, hypertension, dyslipidemia and fatty liver; Combined with weight-bearing joint pain; Causing dyspnea or obstructive sleep apnea syndrome; BMI ≥ 24 with the above complications or BMI ≥ 28 with or without complications can not lose 5% of body weight after 3-6 months treatment with diet and exercise alone.

At present, the main drugs approved by FDA for the treatment of obesity include benzphetamine, diethylpropion, methamphetamine, phendimetrazine, phentermine, phentermine resin complex, orlistat and liraglutide injection. At present, only orlistat has been approved to be marketed in China. Although these weight-loss drugs have some weight-loss effect, the side effects are also of concern and limit their use. For example, bupropion may cause tachycardia and insomnia, phentermine may cause insomnia, dry mouth and constipation, the combination of phentermine and fenfluramine may cause primary pulmonary hypertension and heart valve insufficiency (but phentermine alone has no obvious correlation), orlistat may cause fat-soluble vitamin deficiency, flatulence, defecation urgency, steatorrhea, etc.

1.2 Study Rationale

Glucagon-like peptide-1 receptor (GLP-1R) agonists promote glucose-dependent insulin secretion through activation of peripheral GLP-1 receptors, decrease gastric emptying, and reduce food intake, leading to weight loss. Glucagon is a catabolic hormone, which has a strong role in promoting glycogenolysis and gluconeogenesis, so that the blood glucose significantly increased.

Oxyntomodulin, an endogenous gut peptide hormone, combines the anorectic and glucose-lowering effects of GLP-1R agonists with the GCGR-mediated increase in energy expenditure [11] [12]. GLP-1R knockout mice (GLP-1 R −/−) showed a decrease in body weight after slow infusion of OXM, but to a lesser extent compared with wild-type (WT) mice. This suggests that the weight-reducing effect of OXM requires the simultaneous activation of both GLP1R and GCGR receptors [13]. Preclinical data from rodents suggest that GLP-1R/GCGR agonists reduce body weight more effectively than GLP-1R agonists. Likewise, Lao et al reported that their dual GLP-1R/GCGR agonist showed higher weight loss in diet-induced obese rhesus monkeys [14] [15]. Preclinical data for IBI362 also showed significant weight loss (loss of appetite, weight loss) in diet-induced obese mice.

The study drug, IBI362, is an OXM analog (OXM3). Endogenous OXM is thought to activate both GLP-1R and GCGR, which play an important role in blood glucose

regulation and weight loss. Studies have shown that endogenous OXM activates GLP-1R more weakly than GCGR during cAMP signaling-related ERK1/2 phosphorylation, but activates GLP-1R and GCGR at similar ratios during cAMP-mediated Ca2 + influx [16]. [redacted]

[redacted]

[redacted]

[redacted]

1.3 Potential Risks and Benefits of Treatment Options

1.3.1 Potential Risks for IBI362 Identified Based on Preclinical Safety Studies

[redacted]

[redacted]

[redacted]

1.3.2 Potential Risks for IBI362 Based on Clinical Safety Studies

[redacted]

[redacted]

[redacted]

[redacted]	[redacted]							
[redacted]	[redacted]	[redacted]	[redacted]	[redacted]	[redacted]	[redacted]	[redacted]	[redacted]
[redacted]								
[redacted]					[redacted]	[redacted]	[redacted]	[redacted]
[redacted]						[redacted]	[redacted]	[redacted]
[redacted]						[redacted]		[redacted]
[redacted]					[redacted]			
[redacted]								
[redacted]		[redacted]			[redacted]	[redacted]	[redacted]	[redacted]

[redacted]		[redacted]			[redacted]	[redacted]	[redacted]	[redacted]
[redacted]								
[redacted]							[redacted]	[redacted]
[redacted]								
[redacted]	[redacted]							[redacted]
[redacted]								
[redacted]							[redacted]	[redacted]
[redacted]								
[redacted]					[redacted]			[redacted]
[redacted]	[redacted]							[redacted]

[redacted]

[redacted]

[redacted]

[redacted]

[redacted]

[redacted]	[redacted]					[redacted]
	[redacted]	[redacted]	[redacted]	[redacted]	[redacted]	
[redacted]						

[redacted]	[redacted]		[redacted]	[redacted]	[redacted]	[redacted]
[redacted]						
[redacted]				[redacted]	[redacted]	[redacted]
[redacted]	[redacted]	[redacted]	[redacted]	[redacted]		[redacted]
[redacted]	[redacted]		[redacted]		[redacted]	[redacted]
[redacted]			[redacted]	[redacted]		[redacted]
[redacted]			[redacted]	[redacted]		[redacted]
[redacted]			[redacted]			[redacted]
[redacted]					[redacted]	[redacted]
[redacted]	[redacted]					[redacted]
[redacted]					[redacted]	[redacted]
[redacted]				[redacted]		[redacted]
[redacted]				[redacted]		[redacted]
[redacted]						
[redacted]			[redacted]	[redacted]	[redacted]	[redacted]
[redacted]				[redacted]		[redacted]
[redacted]						
[redacted]	[redacted]				[redacted]	[redacted]
[redacted]				[redacted]		[redacted]
[redacted]						
[redacted] [redacted]				[redacted]		[redacted]

[redacted]

[redacted]

[redacted]

[redacted]

[redacted]

[redacted]

	[redacted]		[redacted]		[redacted]	
[redacted]	[redacted]	[redacted]	[redacted]	[redacted]	[redacted]	[redacted]
[redacted]	[redacted]	[redacted]	[redacted]	[redacted]	[redacted]	[redacted]
[redacted]	[redacted]	[redacted]	[redacted]	[redacted]	[redacted]	[redacted]
[redacted]	[redacted]	[redacted]	[redacted]	[redacted]	[redacted]	[redacted]
[redacted]	[redacted]	[redacted]	[redacted]	[redacted]	[redacted]	[redacted]
[redacted]	[redacted]	[redacted]	[redacted]	[redacted]	[redacted]	[redacted]
[redacted]	[redacted]	[redacted]	[redacted]	[redacted]	[redacted]	[redacted]
[redacted]	[redacted]	[redacted]	[redacted]	[redacted]	[redacted]	[redacted]
[redacted]	[redacted]	[redacted]	[redacted]	[redacted]	[redacted]	[redacted]

	[redacted]		[redacted]		[redacted]	
[redacted]	[redacted]	[redacted]	[redacted]	[redacted]	[redacted]	[redacted]
[redacted]	[redacted]	[redacted]	[redacted]	[redacted]	[redacted]	[redacted]

[redacted]

[redacted]

	[redacted]		[redacted]		[redacted]	
[redacted]	[redacted]	[redacted]	[redacted]	[redacted]	[redacted]	[redacted]
[redacted]	[redacted]	[redacted]	[redacted]	[redacted]	[redacted]	[redacted]

	[redacted]		[redacted]		[redacted]	
[redacted]	[redacted]		[redacted]		[redacted]	
	[redacted]	[redacted]	[redacted]	[redacted]	[redacted]	[redacted]
[redacted]	[redacted]	[redacted]	[redacted]	[redacted]	[redacted]	[redacted]
[redacted]	[redacted]	[redacted]	[redacted]	[redacted]	[redacted]	[redacted]
[redacted]	[redacted]	[redacted]	[redacted]	[redacted]	[redacted]	[redacted]
[redacted]	[redacted]	[redacted]	[redacted]	[redacted]	[redacted]	[redacted]
[redacted]	[redacted]	[redacted]	[redacted]	[redacted]	[redacted]	[redacted]
[redacted]	[redacted]	[redacted]	[redacted]	[redacted]	[redacted]	[redacted]
[redacted]	[redacted]	[redacted]	[redacted]	[redacted]	[redacted]	[redacted]

[redacted]

[redacted]

	[redacted]		[redacted]		[redacted]	
[redacted]	[redacted]		[redacted]		[redacted]	[redacted]
	[redacted]	[redacted]	[redacted]	[redacted]	[redacted]	[redacted]
[redacted]	[redacted]	[redacted]	[redacted]	[redacted]	[redacted]	[redacted]
[redacted]	[redacted]	[redacted]	[redacted]	[redacted]	[redacted]	[redacted]
[redacted]	[redacted]	[redacted]	[redacted]	[redacted]	[redacted]	[redacted]
[redacted]	[redacted]	[redacted]	[redacted]	[redacted]	[redacted]	[redacted]
[redacted]	[redacted]	[redacted]	[redacted]	[redacted]	[redacted]	[redacted]
[redacted]	[redacted]	[redacted]	[redacted]	[redacted]	[redacted]	[redacted]
[redacted]	[redacted]	[redacted]	[redacted]	[redacted]	[redacted]	[redacted]
[redacted]	[redacted]	[redacted]	[redacted]	[redacted]	[redacted]	[redacted]
[redacted]	[redacted]	[redacted]	[redacted]	[redacted]	[redacted]	[redacted]
[redacted]	[redacted]	[redacted]	[redacted]	[redacted]	[redacted]	[redacted]
[redacted]	[redacted]	[redacted]	[redacted]	[redacted]	[redacted]	[redacted]
[redacted]	[redacted]	[redacted]	[redacted]	[redacted]	[redacted]	[redacted]
[redacted]	[redacted]	[redacted]	[redacted]	[redacted]	[redacted]	[redacted]
[redacted]	[redacted]	[redacted]	[redacted]	[redacted]	[redacted]	[redacted]
[redacted]	[redacted]	[redacted]	[redacted]	[redacted]	[redacted]	[redacted]
[redacted]	[redacted]	[redacted]	[redacted]	[redacted]	[redacted]	[redacted]
[redacted]	[redacted]	[redacted]	[redacted]	[redacted]	[redacted]	[redacted]
[redacted]	[redacted]	[redacted]	[redacted]	[redacted]	[redacted]	[redacted]

	[redacted]	[redacted]	[redacted]
[redacted]	[redacted]	[redacted]	[redacted]
[redacted]			

[redacted]

[redacted]

[redacted]

[redacted] t

[redacted]

[redacted]

[redacted]

[redacted]

[redacted]

2 Study Objectives and Endpoints

2.1 Study Objectives

2.1.1 Primary Objective

- To investigate the safety and tolerability of multiple subcutaneous injections of IBI362 in overweight or obese subjects and to determine the safe dose range for clinical use.

2.1.2 Secondary Objectives

- To investigate the pharmacokinetic/pharmacodynamic (PK/PD) parameters of multiple subcutaneous injections of IBI362 in overweight or obese subjects.

2.2 Study Endpoints

2.2.1 Primary Endpoint

- Safety and tolerability: incidence of various adverse events (including chief complaints of subjects, physical examination, laboratory tests (hematology, blood biochemistry, blood lipids, coagulation function, urinalysis, myocardial enzymes, serum amylase and lipase, thyroid function, serum calcitonin, etc.), vital signs (pulse, respiration, blood pressure, body temperature), 12-lead ECG abnormalities and hypoglycemic events, etc.) in different dose periods during the escalation process, The name, clinical characteristics, severity, onset and end time, treatment and outcome of adverse events were recorded, the correlation between adverse events and the study drug was determined, and the medication compliance was analyzed.
- Maximum tolerated dose: if "dose escalation stopping criteria" are met at a given dose level and dose exploration is stopped, the previous dose is the maximum tolerated dose; If the "dose escalation stopping criteria" are still not met when the maximum escalation dose is reached, the maximum tolerated dose is greater than or equal to the maximum escalation dose.

2.2.2 Secondary Endpoints

- To assess the pharmacokinetic parameters of IBI362 in overweight or obese subjects, including but not limited to: T_{max}, C_{max}, Area Under Curve (AUC), volume of distribution (V_d), half-life ($T_{1/2}$), clearance (CL), accumulation coefficient (AR);
- To evaluate the pharmacodynamic parameters of fasting plasma glucose, fasting glucagon, fasting insulin, fasting C-peptide, endogenous oxyntomodulin (OXM) and glucagon-like peptide 1 (GLP-1) before and after multiple doses of IBI362;
- Changes from baseline in fasting body weight, waist-to-hip ratio, BMI, blood pressure, pulse rate, blood lipids and HbA1c at steady state in each dose period during dose escalation;
- Occurrence of anti-drug antibody (ADA) and neutralizing antibody (NAb) against IBI362 in serum before and after administration;
- Pancreatic β-cell function and insulin resistance (HOMA model) were evaluated.
- To assess the effect of IBI362 on serum uric acid and alanine aminotransferase in overweight or obese subjects.

2.2.3 Exploratory Endpoints

- Evaluate the total body fat content, waist fat content, hip fat content and waist-hip fat ratio measured by dual-energy X-ray absorptiometry (DEXA), calculate lean body mass

(lean mass = body weight-fat weight), and compare the changes of each indicator from baseline after 12 weeks and 16 weeks of administration;

- Evaluate intra-abdominal fat area (VFA), subcutaneous fat area (SFA) and total abdominal fat area (TFA) measured by MRI, and compare the changes of each index from baseline after 12 weeks and 16 weeks of administration.

3 Overall Study Design

3.1 Design Rationale

This is the first study to assess the safety, tolerability, and PK/PD of multiple injections of IBI362 in Chinese overweight or obese subjects. A multicenter, randomized, double-blind (subject, investigator) and placebo-controlled trial design was used.

3.1.1 Dose Selection Rationale

Five dosing regimens of IBI362 (1mg QW*4+2mg QW*4+3mg QW*4; 1.5 mg QW*4+3mg QW*4+4.5 mg QW*4; 2mg QW*4+4mg QW*4+6mg QW*4) are proposed for Chinese subjects; 2.5 mg QW*4+5mg QW*4+7.5 mg QW*4+10mg QW*4; 3mg QW*4+6mg QW*4+9mg QW*4). The dose selection and interval design for this tolerability study were mainly based on the following considerations:

1) Safe starting dose

The starting doses for the additional cohorts are 2.5 mg and 3mg, respectively, and based on the PK data of IBI362 in Chinese weight-reduced/obese subjects, the maximum starting dose of 3mg results in a (typical) maximum plasma concentration (Cmax) that is < the drug concentration that has been shown to be safe (2.5 mg) in the US study in healthy subjects.

2) Low drug exposure

Phase data indicate that Chinese overweight/obese subjects have lower single-dose drug exposure than healthy US subjects in OXAA or OXAB studies at equivalent doses. The actual drug exposure in Chinese subjects was lower than the exposure prespecified based on the PK profile in healthy US subjects (OXAA and OXAB studies) at the time of the design of the weight loss indication regimen. Based on the actual drug exposure in Chinese overweight and obese subjects, once weekly dosing with slow titration to 10mg was comparable to the pre-specified exposure of 6mg (Cohort 3) prior to the start of the Chinese weight reduction study.

3) Inducible tolerance potential

The results of single-dose (0.03, 0.1, 0.3, 1.0, 2.5, 5.0 mg) escalation studies conducted in Japanese and US subjects showed that 2.5 mg was the maximum tolerated dose (MTD) of single-dose study; However, 4 doses of 1.5 mg QW (weekly), with in vivo exposures > 2.5 mg single dose, still demonstrated a tolerable safety profile,

suggesting an inducible tolerability of IBI362. The results of the multiple-dose study in Chinese subjects further confirmed the inducible tolerability of IBI362.

4) Sophisticated safety monitoring method and slow climbing speed

The major adverse effects of OXM targets are gastrointestinal and easy to monitor. 2.5 mg is the dose that has been verified to be safe for healthy people in the United States. The (typical) maximum plasma concentration (Cmax) produced by 2.5 mg and 3mg in Chinese subjects was < the drug concentration level that has been validated as safe (2.5 mg) in healthy subjects in the United States. Compared with the previous dose group, the increase rate of dose group was 20%, and the increase rate was slow.

5) Potential for better therapeutic benefit

Studies in US subjects have shown that the weight loss effect of IBI362 increases with increasing drug exposure. The body weight loss data for Chinese subjects also showed a dose-dependent trend. While controlling for safety, it is necessary to explore weight loss activity at higher exposures.

3.2 Phase Ib/II Study Design

3.2.1 Overall Study Design

This study has enrolled 36 overweight or obese subjects (Cohort 1, Cohort 2, and Cohort 3) with weight change of less than 5% controlled by diet and exercise for at least 12 weeks. All subjects were safe and well tolerated (see Section 1.3. 2 and 1.3. 3 for details). Based on the current PK data of subjects in Cohorts 1-3 showing that the plasma concentration in Chinese overweight/obese population is lower than the plasma concentration prespecified in the study, Cohort 4 and Cohort 5 will be further explored, with 12 subjects in each group, and an estimated total of 24 subjects will be added based on the Chinese PK data of IBI362. The entire trial period consisted of a 3-week screening period, a 12-to 16-week double-blind treatment period, and an 8-week follow-up period after the last dose.

The double-blind treatment phase was divided into five cohorts, Cohort 1 (n=12), Cohort 2 (n=12), Cohort 3 (n=12), Cohort 4 (n=12), and Cohort 5 (n=12), and subjects in each cohort were randomized in a 2: 1 ratio to IBI362 treatment (n=8) and placebo (n=4). The SC dosing regimens of IBI362 or placebo in Cohort 1, Cohort 2, Cohort 3, Cohort 4, and Cohort 5 are described as follows:

Cohort 1

The starting dose is 1.0 mg once weekly for consecutive 4 weeks. If the subject is well tolerated #, the dose will be up-titrated to 2.0 mg once weekly for consecutive 4 weeks. The tolerability will be observed again. If the subject is well tolerated #, the dose will be up-titrated to 3.0 mg once weekly for consecutive 4 weeks. (The dose will be increased by 1mg every 4 weeks to the target dose).

Cohort 2

The starting dose is 1.5 mg, administered once a week for consecutive 4 weeks. If the subject is well tolerated #, the dose will be up-titrated to 3.0 mg, administered once a week for consecutive 4 weeks. The tolerability of the subject will be observed again. If the subject is well tolerated #, the dose will be up-titrated to 4.5 mg, administered once a week for consecutive 4 weeks (the dose will be increased by 1.5 mg every 4 weeks to the target dose).

1If subjects in Cohort 2 are intolerant to 3.0 mg or 4.5 mg, the dose should be adjusted according to the criteria in Table 1.

Cohort 3

The subjects in Cohort 2 can only start the administration after the subjects in Cohort 2 have completed the administration of 1.5 mg for 4 weeks and are well tolerated; If 1.5 mg is not tolerated by subjects in Cohort 2, 2.0 mg and higher will not be explored for subjects in Cohort 3.

In this cohort, the starting dose is 2.0 mg once weekly for consecutive 4 weeks. If the subject has good tolerance #, the dose will be increased to 4.0 mg once weekly for consecutive 4 weeks. The tolerability will be observed again. If the subject has good tolerance #, the dose will be increased to 6.0 mg once weekly for consecutive 4 weeks. (The dose will be increased by 2mg every 4 weeks to the target dose).

Cohort 4

The starting dose of the subjects is 2.5 mg, once a week, after continuous administration for 4 weeks; If the subject is well tolerated #, the dose will be up-titrated to 5.0 mg once a week for consecutive 4 weeks; Observe the tolerance of the subjects again. If the tolerance is good #, the dose will be increased to 7.5 mg once a week for consecutive 4 weeks; Observe the tolerability of the subjects again. If the tolerability is good #, the dose will be increased to 10.0 mg once a week for consecutive 4 weeks (the dose will be increased by 2.5 mg every 4 weeks to the target dose).

Cohort 5

The starting dose of the subjects is 3.0 mg, once a week, after continuous administration for 4 weeks; If the subject is well tolerated #, the dose will be up-titrated to 6.0 mg, once a week for consecutive 4 weeks; Observe the tolerability of the subjects again. If the tolerability is good #, the dose will be increased to 9.0 mg once a week for consecutive 4 weeks (increase the dose by 3.0 mg every 4 weeks to the target dose).

(I) Stop criteria for dose escalation: If any of the following criteria are met, the dose escalation will be terminated.

- Heart rate > 100 beats per minute occurred in > 50% of subjects taking IBI362 within

the same dose.

- Occurrence of at least one treatment-related serious adverse event (other than those expected to be related to the pharmacological properties of IBI362, such as hypoglycemia).
- "No less than 50% of subjects experienced symptomatic hypoglycemic events with plasma glucose levels < 2.8 mmol/L at a given dose level, and these events were considered related to IBI362."
- No less than 2 subjects who receive IBI362 develop persistent (more than one week) symptoms characteristic of acute pancreatitis.
- Non-serious AEs of severe intensity that are related to IBI362 (refer to Section 7.3 of the protocol) occurring in no less than 2 subjects in the same dose group, regardless of whether or not the event is associated with the same organ or system (other than gastrointestinal effects).

Note: In case of AE related to dose escalation stop, please report to the sponsor as per Appendix 6.

(II) Intolerance criteria for a single subject:

- If venous plasma glucose < 2.8 mmol/L; Or if the venous plasma glucose does not reach the standard of < 2.8 mmol/L, but the symptoms of hypoglycemia are obvious and cannot be recovered spontaneously (within 15min), and the re-test before the next dose still meets the above standard, it is considered that the patient is intolerant at this dose;
- If there are other intolerance conditions related to the study drug that the investigator considers to be related to the study drug, and the investigator thinks it is necessary to stop the exploration of the subject at this dose after discussion with the sponsor, this case is considered to be intolerable at this dose.

3.2.2 Actions Taken to Avoid Bias

The possible bias was not avoided. The double-blind placebo-controlled study was used in this study. Meanwhile, the safety and efficacy measurements were objective measurements, and the artificial bias was small.

3.2.3 Definition of End of Study

The end of the study was defined as the time when the last subject completed the last visit.

4 Study Population

Subject Eligibility: Deviations from eligibility criteria may impact the scientific integrity of the study, regulatory acceptability, and/or subject safety and are not permitted. Therefore, subjects must meet the protocol-specified criteria.

4.1 Inclusion Criteria

Subjects met all of the following criteria to be included in the study:

1. Age 18 ~ 75 years (both inclusive), male or female;
2. Obese: BMI ≥ 28. 0 kg/m^2; Or overweight: 24 ≤BMI < 28.0 kg/m^2 with at least one of the following manifestations: i. Strong appetite, unbearable hunger before meal, and more food intake per meal; ii. Patients with one or more of pre-diabetes (impaired fasting glucose and/or impaired glucose tolerance), hypertension, dyslipidemia (see Annex 4 for reference standards), and fatty liver (within 6 months prior to screening); iii. Combined weight-bearing joint pain; iv. Obesity-induced dyspnea or obstructive sleep apnea syndrome;
3. Controlled by diet and exercise alone for at least 12 weeks at screening, with body weight change of less than 5%;
4. Able to understand the procedures and methods of this study, willing to strictly comply with the clinical trial protocol to complete this trial, and voluntarily sign the informed consent form.

4.2 Exclusion Criteria

Subjects will be excluded from the study if they meet any of the following criteria:

1. Subjects who are suspected by the investigator to be allergic or have allergic constitution to the study drug or ingredients;
2. Use of any of the following medications or treatments prior to screening:

   1）Previous use of GLP-1 receptor (GLP-1R) agonists or GLP-1R/GCGR agonists;

   2）Use of drugs that affect body weight within 3 months prior to screening, including systemic steroids (intravenous, oral or intra-articular administration), metformin, SGLT2 inhibitors, thiazolidinediones (TZDs), tricyclic antidepressants, psychiatric drugs or sedative drugs (such as imipramine, amitriptyline, mirtazapine, paroxetine, phenelzine, chlorpromazine, thioridazine, clozapine, olanzapine, valproic acid, valproic acid derivatives, lithium salts);

   3）Use of Chinese herbal medicine or health products affecting body weight within 3 months prior to screening.

   4）Have used or are currently using weight loss drugs within 3 months prior to screening, such as: sibutramine hydrochloride, orlistat, phentermine, phenylpropanolamine, chlorpheniramine, phentermine, bupropion, lorcaserin, phentermine/topiramate mixture, naltrexone/bupropion mixture, etc.;

   5）Participation in other clinical trials (treated with an investigational drug) within 3 months prior to screening.

3. History or evidence of any of the following prior to screening:

1) Subjects diagnosed with diabetes mellitus according to WHO1999 criteria;

2) Fasting venous blood glucose ≥ 7. 0 mmol/L at screening or venous blood glucose ≥ 11. 1 mmol/L 2 hours after 75g oral glucose tolerance test (OGTT) glucose load (for subjects with fasting blood glucose of 6.1-7.0 mmol/L at screening, venous blood glucose 2 hours after OGTT glucose load should be collected for confirmation);

3) Subjects with retinopathy in the past or at screening;

4) Obesity caused by secondary diseases or drugs, including: increased cortisol hormone (such as Cushing's syndrome), obesity caused by pituitary gland and hypothalamus injury, obesity caused by reduction/withdrawal of weight-reducing drugs, etc.;

5) Previous bariatric surgery or acupuncture for weight loss within 1 year before screening;

6) History of depression in the past; Or have a history of severe mental illness in the past, such as: schizophrenia, bipolar disorder, etc.;

7) Uncontrolled hypertension at screening after treatment with antihypertensive drugs for at least 4 weeks, defined as: systolic blood pressure > 140mmHg and/or diastolic blood pressure > 100mmHg;

8) Systolic blood pressure < 90mmHg and/or diastolic blood pressure < 50mmHg at screening;

9) History of malignancy (except cured basal cell carcinoma of the skin and carcinoma in situ of the cervix) at the time of screening;

10) Heart-related diseases (such as angina pectoris, myocardial infarction, cardiomyopathy, acute and chronic heart failure, etc.) at screening;

11) Hemorrhagic or ischemic stroke or transient ischemic attack within 6 months prior to screening;

12) History of thyroid C-cell carcinoma, MEN (multiple endocrine neoplasia) 2A or 2B syndrome, or relevant family history at screening;

13) History of acute or chronic pancreatitis, gallbladder disease, or pancreatic injury at screening;

14) Chronic gastrointestinal disease, systemic disease that may affect gastrointestinal motility at screening, or use of drugs that may alter gastrointestinal motility, appetite or absorption within 3 months prior to screening;

15） Existence of limb deformity or disability, unable to accurately determine height, weight and other indicators;

16） Major and medium-sized surgery, severe trauma, severe infection within 1 month prior to screening, which is not suitable to participate in the investigator as judged by the investigator;

17） Previous suicidal tendency or suicidal behavior;

18） Anticipated surgery during the trial, except for outpatient surgery that has no effect on the safety of subjects and trial results as judged by the investigator;

19） Subjects who are positive for human immunodeficiency virus (HIV) antibody or hepatitis B surface antigen (HBsAg) or hepatitis C (HCV) antibody or syphilis antibody at screening;

20） History of alcohol abuse within 1 month prior to screening. Average weekly alcohol intake of more than 21 units for men and 14 units for women, or unwillingness to stop drinking 24 hours before the dosing day and throughout the study (1 unit = 360ml of beer, or 150ml of red wine, or 45ml of distilled spirits/liquor);

21） Positive urine screening test for drugs and drugs of abuse at screening.

4. Any laboratory test indicator meeting the following criteria (if there is a clear reason for retest at screening, it can be retested within one week, and the investigator should record the reason for retest):

1) Serum calcitonin ≥ 15 ng/L at screening;

2) Alanine aminotransferase ≥ 2.0 × ULN and/or aspartate aminotransferase ≥ 2.0 × ULN and/or total bilirubin ≥ 1.0 × ULN and/or alkaline phosphatase ≥ 2.0 × ULN at screening;

3) Glomerular filtration rate eGFR < 60 mL/min/1. 73 m^2 at screening, as estimated by the CKD-EPI equation (see Appendix 2);

4) Abnormal thyroid function (FT3, FT4, or TSH) at screening;

5) Fasting triglycerides ≥ 5. 64 mmol/L (500 mg/dl) at screening, if the subject is on lipid-modifying therapy, the drug dose must be stable for 30 days prior to screening;

6) Blood amylase or lipase > 2.0 × ULN at screening;

7) International normalized ratio (INR) of prothrombin time greater than the upper limit of normal at screening.

5. Heart rate < 50 beats/min or > 90 beats/min on 12-lead ECG at screening;

6. Subjects with the following clinically significant 12-lead electrocardiograms (ECGs) abnormalities at screening: second or third degree atrioventricular block without a pacemaker, long QT syndrome or QTcF > 450ms (see Appendix 3 for calculation formula), PR interval < 120ms or PR interval > 220ms, QRS > 120ms, left or right bundle branch block, pre-excitation syndrome, or serious arrhythmia requiring treatment;
7. Pregnant or lactating females, males or females of childbearing potential not willing to use contraception throughout the study;
8. Blood donation and/or blood loss ≥ 400 mL or bone marrow donation within 3 months prior to screening, or presence of hemoglobinopathy, hemolytic anemia, sickle cell anemia, or hemoglobin < 110g/L (male) or < 100g/L (female);
9. The subject has any other factors that may affect the efficacy or safety evaluation of this study, and is not suitable for participation in this study in the opinion of the investigator.

4.3 Restrictions during the Study

- Subjects should not donate blood during their participation in this study and within 8 weeks after the last dose;
- Men or women of childbearing potential must use contraception during the study and for 8 weeks after the last dose.
- Not allowed to participate in other clinical trials;
- During the trial, the original diet, exercise and lifestyle were maintained steadily, and binge eating and strenuous exercise were taboo;
- Alcohol consumption was prohibited during the trial.

4.4 Subject Screening

4.4.1 Enrollment Procedure

The investigator will enroll subjects as follows:

1. Obtain informed consent signed by the subject or the subject's legally acceptable representative prior to any study-related procedures.

2. Subject eligibility will be formally determined by the Principal Investigator or appropriately trained designee after reviewing the inclusion/exclusion criteria.

Subjects who do not meet the relevant criteria for this study (screen failures) may be rescreened. If a subject is to be considered for re-screening, the investigator must contact the sponsor medical monitor. Each subject may be rescreened once. At the time of rescreening, the subject's legally acceptable representative must re-sign the Informed Consent Form (ICF) and will be reassigned with a screening number.

4.4.2 Handling Procedures for Incorrectly Enrolled Subjects

The inclusion criteria must be strictly followed. If a subject is found to be enrolled that does not meet the eligibility criteria, the sponsor's medical monitor and the investigator will discuss whether to continue the subject in the study.

4.4.3 Randomization and Blinding

"This was a multicenter, randomized, double-blind (subject, investigator), placebo-controlled study, as described in Section 8.6."

4.5 Subject Replacement, Discontinuation, and Withdrawal Criteria

4.5.1 Subject Replacement

Subjects who are not dosed after randomization will be replaced, unblinded and assigned a randomization number offline by the sponsor's unblinded statistician.

4.5.2 Discontinued medication

If a subject experiences any of the following conditions during the study, the investigator should consider discontinuing study drug after discussion with the sponsor:

1）Marked increase from baseline in liver enzymes as defined in any of the following Table 13:

Table 13. Liver Enzyme Elevations at Baseline and during the Treatment Period

Baseline	Treatment Period
ALT/AST < 1.5 x ULN	ALT Or AST ≥ 5 Times ULN
	ALT or AST ≥ 4 times ULN for more than 2 weeks
	ALT or AST ≥ 3 times ULN and TBIL > 2 times ULN
	ALT or AST ≥ 3 times ULN and International Normalized Ratio (INR) ≥ 1.5
	ALT or AST ≥ 3 times ULN with fatigue, nausea, vomiting, right upper quadrant pain/tenderness, fever, rash
	ALP > 3X ULN
	ALP ≥ 2.5 X ULN and TBIL > 2X ULN
	ALP ≥ 2.5 times ULN with fatigue, nausea, vomiting, right upper quadrant pain/tenderness, fever, rash
ALT/AST ≥ 1.5 and < 2X ULN	ALT or AST ≥ 4 times baseline level
	ALT or AST ≥ 3 times baseline level for more than 2 weeks
	ALT or AST ≥ 2 times baseline and TBIL > 2 times ULN
	ALT or AST ≥ 2 times baseline level and international normalized ratio (INR) ≥ 1.5

Baseline	Treatment Period
	ALT or AST ≥ 2 times baseline with fatigue, nausea, vomiting, right upper quadrant pain/tenderness, fever, rash

2) CK value > 5 times ULN;

3) Blood lipase and/or amylase ≥ 3 times ULN. Refer to Annex 1 for subsequent monitoring and treatment procedures;

4) The subject experienced a severe hypoglycemic event (Grade 3, refer to Section 7.7. 2);

5) Persistent Grade 1 or 2 hypoglycaemic events (persistent hypoglycaemia is defined as at least 1 hypoglycaemic event per day for ≥ 2 days).

4.5.3 Withdrawal Criteria

Including subject withdrawal on his own initiative and subject withdrawal at the discretion of the investigator:

1) The investigator considers that the subject should not continue to participate in the trial if the adverse event that the investigator considers is necessary to stop the treatment occurs (for gastrointestinal adverse reactions, it is considered to be related to drug effect, and it is recommended that the investigator may consider that the subject withdraws from the study if severe adverse reactions still exist after dose adjustment in combination with specific clinical conditions);
2) If pancreatitis is confirmed by clinical symptoms or imaging examination, the trial will be terminated immediately;
3) Subjects who have poor compliance, no longer receive medication or tests before completing all the trials, and cannot insist on completing the trial as planned, including subjects who are unable to control diet well, fail to take medication as prescribed, or have other factors that may affect the efficacy observation;
4) Participants in other clinical trials during the trial, and participation in other trials is defined as signing the informed consent form for other trials;
5) Withdrawal of informed consent by the subject;
6) Unblinding due to various reasons;
7) Female subject is pregnant;
8) The investigator considers that it is not suitable to continue to participate in this clinical trial.

4.5.4 Exit Procedure

If a subject decides to withdraw from the trial, an early withdrawal visit should be performed and then withdrawn from the study. Whenever possible, subjects were to return to the study site for an early withdrawal visit. The date of the subject's last injection of study drug and the reason for withdrawal were recorded in the eCRF.

4.6 Subject Withdrawal from the Study

If a subject discontinues study treatment and voluntarily withdraws consent for follow-up data collection, data collection should not continue; However, subjects who discontinue study drug but do not withdraw consent may continue to participate in study follow-up for safety/efficacy assessments.

5 Study Drug and Other Treatments

5.1 Treatment Assignment

5.1.1 Subject Number and Treatment Assignment

After signing the informed consent form, the subject will be given the subject number, and then the screening examination will be performed. If the screening is successful, the subject will be randomly assigned to five treatment groups of Cohort 1, Cohort 2, Cohort 3, Cohort 4 and Cohort 5, with investigational drug: placebo = 8: 4 in each cohort group, with a total of 12 subjects.

5.1.2 Randomization and Blinding

Randomized double-blind trial in which subjects, investigators, data analysts, and all medical personnel involved in the treatment or clinical assessment will remain blinded to the true status of the treatment from the time of randomization until the database is locked, using the following methods:

Different drug numbers correspond to random numbers in the centralized randomization system, and the blind codes of drug numbers and random numbers remain unknown to the subjects and investigators throughout the trial. The random list shall be kept by a specially-assigned person.

The investigational drug and placebo will use exactly the same packaging, labeling, administration time, appearance, taste and smell to conceal the true situation of the treatment drug.

Unblinding was to be performed only in the event of an emergency for a subject and at the end of the study.

Unblinded Sponsor personnel will not be blinded during the course of this study.

5.2 Study drug

5.2.1 Physical and chemical characteristics of the study drug

Molecular Weight:	4560.32 Daltons
Description:	White to off-white powder
PI:	5.2
Solubility:	Solvent: 20 mM Tris buffer at pH 8.0 and 150 mM sodium chloride.<br>Solubility: Not less than 10 mg/mL at 25 ℃.<br>Solubility Description: Sparingly or slightly soluble.

5.2.2 Study Drug Strength and Manufacturer

The IBI362 drug product is IBI362 for injection and consists of 2 mg IBI362 and the inactive ingredients tris (hydroxymethyl) aminomethane, mannitol and sucrose. The vial contents are reconstituted with sterile water for injection to obtain a clear solution of IBI362.

"The placebo is an IBI362 mimetic, and the placebo consists of the inactive ingredients tris (hydroxymethyl) aminomethane, mannitol, and sucrose." The vial contents are reconstituted with sterile water for injection to obtain a clear solution devoid of active ingredients.

The drug product strength used in this study was 2 mg/vial, and the placebo strength matched the drug product and was provided by the Sponsor.

5.2.3 Storage

The drug product and placebo should be stored under refrigerated conditions (2 °C to 8 °C).

5.2.4 Mode of administration

Both IBI362 and placebo will be administered by subcutaneous injection once a week. Each dose should be prepared and injected by the study nurse. See the Instructions for Use for details of the preparation and use of the study drug.

5.3 Concomitant Therapy

5.3.1 Prohibited Medications

The following medications and measures are contraindicated:

1. Growth hormone and its analogues;
2. Any systemic corticosteroid (including intravenous, oral, intra-articular) administered for ≥ 7 days. Corticosteroid hormone: Mainly for glucocorticoid hormone, including short-acting: hydrocortisone, cortisone; Intermediate effect: prednisone, prednisolone, methylprednisone, triamcinolone; Long-acting:

dexamethasone, betamethasone, etc.;

3. Drugs for weight control, such as sibutramine hydrochloride, orlistat, phentermine, phenylpropanolamine, chlorpheniramine, phentermine, bupropion, lorcaserin, phentermine/topiramate mixture, naltrexone/bupropion mixture and some health products with "weight loss" indications;
4. Drugs that have an effect on body weight: tricyclic antidepressants, drugs for psychiatric disorders and neuroleptics such as imipramine, amitriptyline, mirtazapine, paroxetine, phenelzine, chlorpromazine, thioridazine, clozapine, olanzapine, valproic acid, valproic acid derivatives, lithium salts; Antidiabetic agents: metformin, SGLT2 inhibitors, and other agents that can cause weight gain or loss.
5. Any drug or herbal medicine known to have common toxic effects on major organs, or any drug that may interfere with the interpretation of efficacy and safety data.

5.4 Dosing during pregnancy, childbearing potential, or lactation

5.4.1 Pregnancy

Pregnant women should not be enrolled in this study and contraception is required during the trial.

5.4.2 Childbearing age

Female subjects of childbearing potential who are sexually active with a non-sterilized male partner, and non-sterile male subjects who are sexually active with a female partner of childbearing potential must use at least 1 acceptable effective method of contraception from screening until 8 weeks after the last dose and should discuss the discontinuation of contraception with a responsible physician after that time point.

Women were considered postmenopausal after 12 months of menopause without an alternative medical cause. The requirements according to age are as follows:

- Women ≥ 50 years of age are considered postmenopausal if they have been amenorrheic for 12 months or more after cessation of exogenous hormone therapy and their luteinizing hormone and follicle-stimulating hormone levels are in the accepted postmenopausal range.
- Women < 50 years of age were considered postmenopausal if they had been amenorrheic for 12 months or more after cessation of all exogenous hormonal therapy, had been oophorectomized with the last menses occurring > 1 year earlier with radiation therapy, had been amenorrheic with the last menses occurring > 1 year earlier with chemotherapy, or had been surgically sterilized (bilateral oophorectomy or hysterectomy).

5.4.3 Lactation

Lactating women who are breastfeeding cannot be enrolled in this study.

5.5 Treatment compliance

"Study treatment was administered at the study site, and treatment compliance was monitored using drug receipt and dispatch records, subject & '92; s original medical records, and eCRFs."

5.6 Drug Recovery and Destruction

At the end of the study, all remaining study drug will be shipped back to the sponsor or its designee under the supervision of the investigator or designee for destruction in accordance with the institutional Standard Operating Procedure. If local procedures require destruction of study drug supplies at the study site, the study site (if local procedures permit) should retain the study drug supplies until the study monitor checks the accountability records to assess compliance and accuracy of the study site accountability records. If the study drug is destroyed by the site prior to the monitor's inspection, the monitor will evaluate the study drug according to the destruction record (in accordance with the site's SOP).

5.7 Records of Study Drug

The investigator is responsible for drug accountability at the study site, however some drug accountability responsibilities may be assigned to the appropriate pharmacist or other designee. Inventory and accountability records must be maintained and readily available for inspection by the study monitor and all applicable regulatory authorities. The investigator or designee must maintain the following records:

1. Study drug handover records at the study site.
2. Study drug inventory records at the study site.
3. A record of study drug administration by the subject, including the number of units supplied at each time.

The investigational product must be used as specified in the protocol. The investigator will also fully document whether or not the subject received study drug. These records should include the dates and quantities of investigational product dispensed to the study subject, as well as any available batch numbers of investigational product or unique codes assigned to the study subject.

Completed accountability records are archived at the study site. The investigator or designee is expected to collect and retain all used, unused, and partially used study drug containers until verification by the study monitor (unless otherwise agreed by the sponsor).

5.8 Complaint Handling

The sponsor collects product complaints for the study drug and drug delivery system used in clinical studies to ensure the safety of study subjects, monitor quality, and facilitate process and product improvement.

All product complaints related to packaging, labeling and released materials are reported to the sponsor.

The investigator or his/her designee is responsible for reporting complete information on product complaints via email or other written correspondence to the sponsor contact person or to the manufacturer's representative as documented in the packaging information. Any AE associated with a product complaint should be reported as described in Section 7 of this protocol.

If the investigator is required to return the product for investigation, a copy of the product complaint form will be returned with the product.

6 Study Assessments

6.1 Safety and Tolerability Assessments

Safety, tolerability

Incidence of various adverse events (including chief complaints of subjects, physical examination, laboratory tests (hematology, blood biochemistry, blood lipids, coagulation function, urinalysis, myocardial enzymes, blood amylase and lipase, thyroid function, serum calcitonin, etc.), vital signs (pulse, respiration, blood pressure, body temperature), 12-lead ECG abnormalities and hypoglycemic events, etc.) in different dose periods during the escalation process, The name, clinical characteristics, severity, onset and end time, treatment and outcome of adverse events were recorded, the correlation between adverse events and the study drug was determined, and the medication compliance was analyzed.

Maximum tolerated dose

If the "dose escalation stopping criterion" is met at a given dose level and dose exploration is stopped, the previous dose is the maximum tolerated dose; If the "dose escalation stopping criteria" are still not met when the maximum escalation dose is reached, the maximum tolerated dose is greater than or equal to the maximum escalation dose.

6.2 Safety and Other Assessments

6.2.1 Laboratory Tests

6.2. 1.1 Routine Laboratory Safety Assessments

Specific laboratory procedures/assessments are detailed below. The total amount of

blood drawn/collected throughout the trial (from pre-trial to post-trial visits), including the amount of blood drawn/collected per subject per visit and per specimen type. Refer to the laboratory assessments section of the trial flow chart.

6.2. 1.2 Laboratory Safety Evaluations

Laboratory tests including hematology, blood biochemistry, blood lipids, coagulation function, urinalysis, myocardial enzymes, blood amylase, blood lipase and serum calcitonin are shown in Table 14.

Table 14. Routine Laboratory Safety Assessments

Blood routine	WBC, RBC, PLT, HGB, HCT, WBC Differential (ANC, BASO, EOS, MONO, LYM)
Blood biochemistry	AST, ALT, TBIL, DBIL, ALB, TP, GGT, ALP, LDH, serum potassium, serum sodium, serum calcium, serum chloride, UA, Urea, Cr, FBG
Blood lipids	TC, TG, HDL-C, LDL-C
Urinalysis	Urine pH, URPO, UGLU, URBC, UWBC
Myocardial enzyme spectrum	CK, CK-MB
Thyroid function	FT3, FT4, TSH
Serum infectious disease indicators	HBsAg, HBsAb, HBcAb, HBeAg, HBeAb, HCV antibody, HIV antibody, syphilis antibody
Coagulation function indicators	PT, APTT, INR
Other	Blood amylase, blood lipase, serum calcitonin

ALB: albumin; ALP: alkaline phosphatase; ALT: alanine aminotransferase; ANC: neutrophil; APTT: activated partial thromboplastin time; AST: aspartate aminotransferase; BASO: basophils; CK: creatine kinase; CK-MB: creatine kinase isoenzyme; Cr: creatinine; DBIL: direct bilirubin; EOS: eosinophils; FBG: fasting serum glucose; FT3: free triiodothyronine; FT4: free thyroxine; GGT: glutamyl transpeptidase; HBcAb: hepatitis B core antibody; HBeAb: hepatitis B E antibody; HBsAb: hepatitis B surface antibody; HBeAg: hepatitis B E antigen; HBsAg: hepatitis B surface antigen; HCT: hematocrit; HCV: hepatitis C virus; HDL-C: high density lipoprotein cholesterol; HGB: hemoglobin; HIV: human immunodeficiency virus; INR: international normalized ratio; LDH: lactate dehydrogenase; LDL-C: low density lipoprotein cholesterol; LYM: lymphocytes; MONO: monocytes; PLT: platelet; PT: prothrombin time; RBC: red blood cell count; TBIL: total bilirubin; TC: total cholesterol; TG: triglycerides; TP: total protein; TSH: thyroid stimulating hormone; UGLU: urine glucose; URBC: urine red blood cells; URPO: urine protein; UWBC: urine white blood cells; WBC: white blood cell count.

6.2.2 Physical examination

6.2. 2.1 Complete physical examination

The investigator or clinical designee will perform a complete physical examination during the screening period. Complete physical examination includes: general condition, chest, abdomen, skin and mucous membranes, head and neck, lymph nodes, spine and extremities, and neurological evaluation. Clinically significant abnormalities should be

recorded as medical history. In addition, a complete physical examination was performed as specified in the trial flow chart. After signing the informed consent form, newly identified clinically significant abnormalities were recorded as AE.

Refer to Visit Schedule Table 2, Table 4 and Table 6 for examination time.

6.2. 2.2 Targeted Physical Examination

For cycles in the trial flow chart that do not require a full physical examination, the investigator or qualified designee will perform a targeted physical examination as clinically indicated, scheduled prior to dosing of each treatment cycle. New clinically significant abnormal findings should be recorded as AE.

Refer to Visit Schedule Table 2, Table 4 and Table 6 for examination time.

6.2.3 Vital Signs

Subjects' vital signs (including body temperature, pulse, respiration, and sitting blood pressure) will be monitored, recorded, and evaluated at scheduled visits according to the trial procedures and schedule of assessments, and any clinically significant abnormalities will be reported as AE.

Additional monitoring of vital signs assessments may be performed according to standard clinical practice or at the discretion of the investigator as clinically indicated. In the event of an AE/SAE, the investigator may collect additional vital sign records (if applicable) and record them in the original medical records and on the eCRF, along with the date and time of measurement.

Subjects should rest quietly in a seated position for at least 5 minutes prior to measurement of blood pressure, pulse, and respiratory rate, and refrain from drinking coffee, emptying bladder, or strenuous exercise for 30 minutes prior to measurement of blood pressure. At each visit, the sitting blood pressure of subjects should be measured for at least 2 consecutive times, with an interval of 1-2 minutes. The investigator should record the mean value of blood pressure, date and time of measurement in the original medical records and eCRF. For the same subject, the blood pressure of the ipsilateral upper arm should be measured at each visit.

Refer to Visit Schedule Table 2, Table 4 and Table 6 for examination time.

6.2.4 ECG

The subject must remain in a supine position for at least 5 minutes in a quiet state before performing the 12-lead ECG. If the subject was unable to lie supine, he/she was to lie recumbently as

12-lead electrocardiogram (ECG) parameters include: RR interval, PR interval, heart rate (HR), QT interval, QTcF (refer to Appendix 3 for calculation formula).

All ECGs were to be reviewed by the principal investigator or designated site

physician and any clinically significant abnormalities were to be reported as AE. After review and signature, the original ECG tracings should be kept together with the subjects' original records. When requested by the sponsor, a copy of the original ECG should be transmitted to the Innovent after the subject's identification has been concealed. Additional ECGs may be performed and recorded in the original medical records and eCRF at the discretion of the investigator according to standard clinical practice or as clinically indicated. The investigator should record the date and time of measurement in the appropriate part of the original medical records.

Refer to Table 2, Table 4 and Table 6 of visit schedule for examination time

6.2.5 Fasting body weight measurement

For each subject, weight measurements should be performed in a uniform manner at each clinic visit using a calibrated scale (either mechanical or electronic). Each weight measurement of the subject should be performed on the same scale after the subject has emptied his bladder. For weight measurement, subjects should remove weight-added clothing such as coats/hats/scarves/waistbands, and shoes.

a. Ensure that the pointer of the scale is zeroed or the electronic scale is zeroed before weighing.

b. Make sure the scale is placed on a firm and smooth surface before weighing (do not place on carpets or sloping surfaces or rough surfaces).

c. Before weighing, the symmetry area of the curtain should be pulled to isolate, so as to protect the privacy of the subject when changing clothing.

d. Ensure that the subject does not feel cold after undressing prior to weighing.

e. After verifying whether the subject has urinated, guide the subject into the weighing area, pull the curtain, and ask the subject to take off the clothing for weight gain such as coat/hat/scarf/waistband, and take off the shoes.

f. Subjects were asked to step on the scale with their feet on each side of the scale. Subjects were asked to stand still with their arms on their sides, and their weight was recorded in kilograms (kg).

Refer to Visit Schedule Table 2, Table 4 and Table 6 for examination time.

6.2.6 Pregnancy test

A blood pregnancy test is required for women of childbearing potential before enrollment. If the pregnancy test is positive, the subject is not eligible/must be discontinued from the study. If pregnancy is suspected during the study, it should be repeated.

6.3 **PK/PD assessment variables**

6.3.1 PK Sample Collection

PK sampling will be performed according to the Schedule of Visits (Table 2, Table 4, Table 6), PK/PD Sampling Schedule (Table 3-1, Table 3-2, Table 5-1, Table 5-2, Table 7-1, Table 7-2), and examinations will be performed at a central laboratory. 3 mL of whole blood was collected into a coagulant vacutainer, plasma was separated, aliquoted and stored frozen for PK analysis of IBI362. Details of sampling methods, sample storage, transportation and analysis were provided in the Laboratory Manual provided by the central laboratory designated by the sponsor.

6.3.2 Pharmacodynamic Sample Collection

Pharmacodynamic sampling will be performed according to the Schedule of Visits (Table 2, Table 4, Table 6), PK/PD sampling schedule (Table 3-1, Table 3-2, Table 5-1, Table 5-2, Table 7-1, Table 7-2), and examinations will be performed at a central laboratory. 9mL of whole blood will be collected for all PD variables (fasting insulin/fasting C-peptide/fasting plasma glucose/fasting glucagon/OXM/GLP-1). Details of sampling methods, sample storage, transportation and analysis were provided in the Laboratory Manual provided by the central laboratory designated by the sponsor.

6.3.3 PK/PD variables

- Pharmacokinetic parameters, including but not limited to Tmax, Cmax, product under the concentration-time curve (AUC), volume of distribution (Vd), half-life (T1/2), clearance (CL) and accumulation coefficient (AR);
- Pharmacodynamic variables, including fasting plasma glucose, fasting glucagon, fasting insulin, fasting C-peptide, endogenous OXM, GLP-1.

6.4 **Immunogenicity evaluation indicators**

6.4.1 Immunogenicity blood collection points

Immunogenicity specimens will be collected according to the schedule of visits (Table 2, Table 4, Table 6). Tests will be performed at a central laboratory.

6.4.2 Immunogenicity indicators

Anti-drug Antibody (ADA) and Neutralizing Antibody (NAb) against IBI362 in serum.

7 Safety Reporting and Adverse Event Management

7.1 **Definition of Adverse Events**

An Adverse Event (AE) is defined as any untoward medical occurrence in a clinical trial subject starting from signing the informed consent form, regardless of whether it is causally related to the study drug, which is judged as an AE, including but not limited to

the following situations:

- Exacerbation of pre-existing (before entering the clinical trial) medical condition/disease (including aggravation of symptoms, signs, laboratory test abnormalities);
- Any newly occurring untoward medical condition (including symptoms, signs, newly diagnosed diseases);
- Abnormal clinically significant laboratory values or results.

7.2 **Definition of Serious Adverse Events**

A serious adverse event is an adverse event that meets at least one of the following criteria:

- Results in death.
- Life-threatening ("life-threatening" in the definition is an AE in which the subject was at risk of death at the time of its occurrence, and does not include an AE that might have caused death if the event were to worsen).
- Requires inpatient hospitalization or prolongation of existing hospitalization, excluding the following:
  a) Rehabilitation facilities;
  b) Nursing home;
  c) Regular emergency room admissions;
  d) Same-day surgery (e.g. Outpatient/same-day/ambulatory surgery);
  e) Hospitalization or prolongation of hospitalization not associated with worsening of an AE is not per se an SAE. For example, hospital admission due to pre-existing disease, without occurrence of new adverse events or aggravation of pre-existing disease (e.g., to check for persistent laboratory abnormalities before the trial); Hospitalization for administrative reasons (e.g., routine annual physical examination); Hospitalization specified in the trial protocol during the clinical trial (e.g., operating according to the requirements of the trial protocol); Elective hospitalization (e.g., elective surgery) that is not associated with worsening of the adverse event; Scheduled treatments or surgeries should be recorded throughout the trial protocol and/or in the baseline data of the individual subject; Admitted for blood product use only.
- Results in permanent or significant disability/incapacity.
- Results in a congenital anomaly/birth defect.

Other Important Medical Events: Important medical events may not be

immediately life-threatening, death, or hospitalization, but are generally considered serious if medical action is required to prevent one of the above situations. Examples include critical treatment in an emergency room or allergic bronchospasm occurring at home, cachexia or convulsions that are not hospitalized, and the development of drug dependence or addiction.

7.3 Severity assessment of adverse events

The investigator will determine the severity according to the grading criteria for adverse events issued by the National Institute on Aging (NIA) [17].

- Mild: The symptoms or signs are perceptible, but easily tolerated and are minor irritants that do not affect normal activities, do not require treatment or medical identification, and are transient.
- Moderate: The event causes a low degree of inconvenience or concern to the subject and may interfere with daily activities, but usually improves with simple treatment; Moderate adverse events may cause some dysfunction.
- Severe: The event interrupts the subject's normal daily life and usually requires systemic medication or other treatment, often resulting in disability.

7.4 Causal relationship judgment between adverse event and investigational drug

The investigator was required to assess the causal relationship between the study drug and each AE and answer "Yes" or "No" to the question "You believe that there is a reasonable possibility that the AE occurred due to the study drug."

When determining that there is a "reasonable possibility" that an AE is due to a drug, the following factors should be considered:

• Time course. Suspect drug exposure. Did the subject actually receive treatment with the suspect drug? Is there a reasonable temporal relationship between the onset of the AE and the suspect drug?

• Consistency of known drug properties. Is the AE consistent with previously reported events for the suspect drug (pharmacology and toxicology) or drugs of the same pharmacology class? Is the occurrence of the AE expected from the pharmacological properties?

• Dechallenge. Did the AE resolve or improve after discontinuation or reduction of the suspect drug?

• No alternative factors. The AE cannot be reasonably explained by another pathology, such as underlying disease, other drugs, other intrinsic or environmental factors.

• Re-challenge. Did the AE reappear after the suspect drug was stopped?

• Possible other causes. Could the adverse event not be explained by an alternative etiology, such as underlying disease, other drugs/vaccines, or other host or environmental factors?

When one or more factors are present, a "reasonable possibility" of an AE needs to be considered.

In contrast, if the above criteria are not applicable, if there is no clear evidence of exposure and a reasonable time course, or if any rechallenge (if performed) is negative or another possible cause of the AE, there may be no "reasonable possibility" of a causal relationship.

Based on the above factors, the investigator's medical assessment of the relationship of the study drug to the AE or its role in the AE was divided into the following two categories:

• Related, i.e., there is a reasonable possibility that the study drug was associated with the onset of the AE: there is evidence of exposure to the study drug. An AE with a reasonable temporal sequence from administration of the sponsor product. The AE is more likely to be explained by the study drug than by other causes.

• Not related, i.e., there is no reasonable possibility of a relationship between the study drug and the onset of the AE: if the subject did not start treatment with the study drug;" Or the AE did not occur in a plausible temporal relationship to exposure to the study drug; Or other factors more likely to explain the occurrence of the AE than the study drug.

7.5 **Recording of Adverse Events**

The investigator should use medical terminology/concepts to record AE or SAE. The use of spoken language and abbreviations should be avoided. All AE (including SAE) should be recorded on the Adverse Event Form of the Electronic Case Report Form (eCRF).

7.5.1 **Adverse event collection and time interval**

Investigators were informed of adverse events by asking subjects non-inducing questions.

All adverse events, including serious adverse events, whether observed by the investigator or spontaneously reported by the subject, were collected from the time of signing the informed consent form until the end of the study. At the end of the study, if the investigator becomes aware that a subject has experienced a serious adverse event related to the study drug or procedure, the event should still be reported to the sponsor.

7.5.2 **Follow-up of adverse events**

Adverse events should be followed up until they have returned to baseline or the

investigator considers that no further follow-up is necessary for reasonable reasons (e.g., no recovery or improvement). If the adverse event cannot be recovered, a reasonable explanation should be recorded in the eCRF. The recovery of the subject's AE or SAE and its date should be recorded in the eCRF and medical records, whether or not related to the study drug.

7.5.3 **Contents of Adverse Event Records**

The investigator should fully record any adverse event, including diagnosis (if no diagnosis, record symptoms and signs including laboratory abnormalities), start and stop dates and times (if applicable), severity and change, whether it is a serious adverse event, action taken with the study drug, treatment given due to the AE and the outcome of the event, and the relationship between the adverse event and the study drug.

For serious adverse events, the investigator should also provide the date the AE meets the criteria for an SAE, the date the investigator learns of the SAE, the rationale why the AE is an SAE, the hospitalization date, the discharge date, the probable cause of death, the date of death, whether an autopsy was performed, causality assessment with study procedures, causality assessment with other drugs, and other possible causes of the SAE. The investigator should also provide the basis for the judgment of relatedness and the description of SAE. In the SAE description, the subject's number, age, gender, height and weight should also be included; Indications and disease stages of the subjects treated with the investigational drug and relevant systemic conditions; Occurrence, development, outcome and outcome of clinical course of SAE; Laboratory test results related to SAE (test time, unit and normal range must be provided); Previous history and concomitant diseases related to SAE as well as their occurrence and duration; Medication history related to SAE, concomitant drugs and their treatment initiation, duration, usage and dosage, etc.; Details of initiation, duration, and administration of study drug.

The items regarding AE recording are described below:

Diagnosis, symptoms and signs

If a diagnosis is already available, the diagnosis should be recorded on the eCRF rather than the individual signs and symptoms (e.g., liver failure should be recorded rather than jaundice, elevated transaminases, and asterixis). If signs and symptoms cannot be ascertained to be caused by the diagnosis at the time of reporting, they will be recorded as a separate AE/SAE. If it is determined that the signs and symptoms are caused by the diagnosis, only the diagnosis is reported separately and the symptoms and signs are included in the diagnosis. AE needs to delete the record of symptoms and signs, and SAE needs to send a follow-up update report.

Adverse Events Secondary to Other Events

In general, adverse events secondary to other events (e.g., caused by other events or

clinical sequelae) should be recorded as the primary event, unless the secondary event is serious or serious. However, secondary events with significant clinical significance should be recorded as separate adverse events in the eCRF if they occur at a different time from the primary event. If the relationship between the events is unclear, they should be recorded separately in the eCRF.

Persistent or Recurrent Adverse Events

A persistent adverse event is an adverse event that persists without resolution between the subject's two evaluation time points.

A recurrent adverse event is an adverse event that has resolved between the two evaluation time points but occurs later. The occurrence of the event should be recorded separately in the eCRF.

Laboratory test abnormality

Clinically significant laboratory abnormalities should be reported as AE. It is the responsibility of the investigator to review all laboratory abnormalities and to make medical judgment as to whether each laboratory abnormality should be reported as an AE.

Pre-existing medical condition

The existing symptoms/signs of subjects during the screening period should be recorded and reported as adverse events only when the severity, frequency and nature of the symptoms/signs are aggravated (except for the deterioration of the disease condition under study) after entering the trial. Changes from the previous state such as "increased headache frequency" should be reflected in the recording.

7.6 Expedited Reporting of SAE and Pregnancy

SAE Reporting:

Since the subject signs the informed consent form, if the subject experiences SAE, the investigator must immediately fill in the Serious Adverse Event Report Form, sign and date it, and immediately report it to the PV Department of the sponsor within 24 hours after the investigator is informed of it: drugsafety@innoventbio.com. For death and life-threatening serious adverse events, the investigator should urgently follow up the missing information and provide a complete SAE report.

At the end of the study, if the investigator becomes aware that a subject has experienced a serious adverse event related to the study drug or procedure, the event should still be reported to the sponsor.

Pregnancy

All subjects of childbearing potential who participate in the clinical trial must take effective contraceptive measures.

If a female subject becomes pregnant during the clinical trial at the time of drug exposure, the subject will be excluded from the study, and the investigator will report the pregnancy to the sponsor within 24 hours of becoming aware of the pregnancy, and the Pregnancy Report/Follow-up Form for Clinical Trials of Innovent will be completed.

If the partner of a male subject at the time of drug exposure becomes pregnant during the clinical trial, the subject continues the clinical trial, reports the pregnancy to the sponsor (drugsafety@innoventbio.com) and completes the Pregnancy Report/Follow-up Form for Innovent clinical trials within 24 hours of the investigator's knowledge of the pregnancy.

The investigator will continuously monitor the subjects who become pregnant and follow up the pregnancy results until 8 weeks after delivery of the mother, and report the results to the sponsor.

If the pregnancy results in stillbirth, spontaneous abortion, fetal anomaly (any congenital anomaly/birth defect) and induced abortion for medical reasons, it will be considered as SAE and should be reported according to the SAE procedure and time limit.

If a subject also experienced an SAE during the pregnancy, it was to be reported according to the SAE reporting procedure.

7.7 Adverse Events of Special Interest (AESI) and Reporting Process

The following adverse events were of special interest in this study: allergic reactions, injection site reactions, transaminase elevations, creatinine elevations, QTc prolongation, gastrointestinal reactions (nausea, vomiting, diarrhea), decreased appetite, dizziness, hypoglycemic events, acute pancreatitis, and severe adverse events of non-serious drug-related adverse events. Investigators should pay close attention to the occurrence of the above adverse events in the clinical study, and timely handle them.

Hypoglycemic events, acute pancreatitis and severe drug-related non-serious adverse events (refer to Section 7.3 of the protocol for severity) should be reported to the sponsor (drugsafety@innoventbio.com; 021-31837255) within 24 hours after awareness, and refer to Appendix 6 for specific procedures.

The above-mentioned Adverse Event of Special Interest (AESI) should be reported according to the SAE reporting process if it meets the criteria of SAE.

7.7. 1 Hepatic Function Abnormal Events

Abnormalities in AST and/or ALT levels accompanied by abnormally elevated total bilirubin levels that meet the conditions in Table 15 and have no other causes of liver injury will be considered as drug-induced liver injury. Such situations should always be considered important medical events.

Table 15. Hepatic Impairment Requiring Reporting as an SAE

**Baseline Period**	Normal (AST/ALT and total bilirubin)	Abnormal (AST/ALT and total bilirubin)
**Treatment Period**	ALT or AST ≥ 3 × ULN<br>With total bilirubin ≥ 2 × ULN<br>And alkaline phosphatase ≤ 2 × ULN<br>And no hemolysis	AST or ALT ≥ 8 × ULN<br>Concomitant total bilirubin increase ≥ 1 × ULN or total bilirubin value ≥ 3 × ULN

Subjects should return to the study site for evaluation as soon as possible (preferably within 48 hours) after learning of an abnormal result. The evaluation should include laboratory tests, detailed medical history and physical assessment, and the possibility of liver neoplasia (primary or secondary) should be considered. In addition to repeat AST and ALT, laboratory tests to be performed should include albumin, creatine kinase, total bilirubin, direct and indirect bilirubin, gamma-glutamyl transferase, prothrombin time/international normalized ratio, and alkaline phosphatase. At the same time, detailed medical history will be collected, including: history of alcohol consumption, acetaminophen, soft drugs, various supplements, traditional Chinese medicine, history of exposure to chemical drugs, family history, occupational exposure, sexual behavior history, travel history, history of contact with subjects with jaundice, surgery, blood transfusion, history of liver disease or allergic disease, history of heart disease, history of immune disease, etc. Further investigations may include tests for acute hepatitis A, B, C, and E, imaging of the liver (e.g., biliary tract), autoantibodies, and cardiac ultrasound. If repeat testing confirms that the laboratory criteria in the table above are met, the possibility of potential drug-induced liver injury should be considered in the absence of other causes of liver function test abnormalities, without waiting for all liver function etiological tests to be made. Such cases of potential drug-induced liver injury should be reported as SAE.

7.7. 2 Hypoglycemic Events

According to the classification criteria for hypoglycemia of the American Diabetes Association (ADA)/European Association of Diabetes (EASD) 2017 version, hypoglycemia is defined as follows:

- Warning level (Grade 1) of hypoglycaemia with a plasma glucose concentration of 70 mg/dl or less (3.9 mmol/L):

  Symptomatic hypoglycemia: a plasma glucose concentration of less than 70 mg per deciliter (3.9 mmol per liter) accompanied by symptoms associated with hypoglycemia.

  Asymptomatic hypoglycemia: a plasma glucose concentration of less than 70 mg per deciliter (3.9 mmol per liter) without symptoms associated with hypoglycemia.

Unclassified hypoglycaemia: plasma glucose concentration no higher than 70 mg per deciliter (3.9 mmol per liter) with no documented information on hypoglycaemic symptoms.

- Clinically significant hypoglycemia (Grade 2) with a plasma glucose concentration of up to 54 mg/dl (3.0 mmol/L):

Symptomatic hypoglycemia: a plasma glucose concentration of less than 54 mg per deciliter (3.0 mmol per liter) accompanied by symptoms associated with hypoglycemia.

Asymptomatic hypoglycaemia: plasma glucose concentration not greater than 54 mg per deciliter (3.0 mmol per liter) without symptoms associated with hypoglycaemia.

Unclassified hypoglycaemia: plasma glucose concentration no higher than 54 mg per deciliter (3.0 mmol per liter) with no documented information on hypoglycaemic symptoms.

- Severe hypoglycemia (Grade 3): Requires assistance of another person to administer carbohydrate, glucagon, or other behavior to aid recovery. During this period, the patient develops a change in state of consciousness, is unable to take care of the aforementioned restorative treatment, is unconscious or semi-unconscious, or becomes comatose (with or without seizure symptoms), and requires intravenous nutrition. During the onset of such symptoms, sometimes the plasma glucose concentration is undetectable at the time of symptom onset, but the symptoms are considered to be directly related to a decrease in blood glucose (plasma glucose concentration not higher than 70 mg per deciliter (3.9 mmol per liter) if neurological symptoms resolve after recovery of the glucose concentration.

  Severe hypoglycaemia requiring medical attention: Severe hypoglycaemic events that require treatment by a healthcare provider (e.g., emergency care provider, emergency room staff).

Other Hypoglycaemic Events

- Relative hypoglycaemic events: symptoms associated with hypoglycaemia occur with a plasma glucose concentration above 70 mg per deciliter (3.9 mmol per liter), but it is expected that the plasma glucose level may rapidly approach the threshold of 70 mg per deciliter (3.9 mmol per liter).
- Possible symptomatic hypoglycaemic events: hypoglycaemic symptoms occur without a plasma glucose level measurement, but are thought to be associated with a decrease in the plasma glucose concentration not higher than 70 mg per deciliter (3.9 mmol per liter).

The investigator will determine whether a hypoglycemic event is severe based on the patient's need for medical assistance in addition to the expected assistance that is routinely received by the patient. All hypoglycaemic events will be recorded in the

hypoglycaemic event module of the electronic event report form, and all severe hypoglycaemic events must be reported as serious adverse events.

Blood glucose self-monitoring

During the study, subjects can monitor their fingerstick blood glucose according to their own conditions, and record it in the subject diary. In case of hypoglycemia (< 2.8 mmol/L), they should contact the investigator in time, and instruct to bring the subject diary and blood glucose meter to visit when coming to the hospital.

7.7. 3 Gastrointestinal Reactions

Nausea, vomiting, and diarrhea are the primary gastrointestinal reactions of interest and will be recorded as AE in the eCRF. Each event was assessed for severity, duration (start and stop dates), and relationship to study drug or protocol procedures as deemed by the investigator.

7.7. 4 Acute pancreatitis

Serum amylase and lipase will be monitored at the time points specified in the protocol, and additional tests may be added at the investigator's clinical discretion.

"Blood amylase and/or lipase ≥ 3 times ULN, even if the subject does not have symptoms of acute pancreatitis, further diagnostic evaluation is required (refer to Appendix 1)"

8 Data Analysis/Statistical Methods

8.1 **Statistical Hypothesis**

The primary endpoint of this study is the safety and tolerability of IBI362. No formal statistical tests will be performed.

8.2 **Sample Size Estimation**

Five dose cohorts were planned for enrollment. A total of 12 subjects per cohort were planned to be enrolled as 8: 4 to IBI362, placebo-controlled. Approximately 60 subjects in total.

8.3 **Statistical Analysis Populations**

Safety set: subjects who signed informed consent and took at least one dose of study drug.

Efficacy Analysis Set: A subset of the Safety Set who had at least one post-baseline assessment.

PK concentration set: includes all subjects who received at least one dose of the study drug and had at least one valid concentration data of the tested components after administration.

PD analysis set: including all subjects who received at least one dose of study drug and had at least one valid test result at baseline and after drug administration.

Anti-drug antibody analysis set: includes all subjects who received at least one dose of study drug and had at least one valid test result.

8.4 **Statistical Analysis**

8.4. 1 General Methods

Measurement data are described with mean, standard deviation, median, maximum and minimum; Count data were described by frequency and percentage. Unless otherwise specified, data will be analyzed separately for subjects within each IBI362 dose group and for placebo subjects.

All statistical analyses were performed using SAS9.2 (or higher).

8.4. 2 Efficacy Analysis

All efficacy analyses were performed on the Efficacy Analysis Set. Descriptive statistics will only be performed for efficacy endpoints in this study. For continuous efficacy endpoints, the corresponding mean and two-sided 95% confidence interval will be calculated for IBI362 subjects and all placebo subjects within each dose group, respectively. The p-value for each dose group of IBI362 versus placebo will be calculated using a two-sample t-test and the corresponding point estimate and 95% confidence interval for the difference will be presented. Missing values will be imputed using the LOCF method. The rates within each dose group will be calculated and 95% confidence intervals will be calculated using Clopper-Pearson for the typed efficacy endpoints, and the chi-square test will be used to compare the differences between IBI362 and placebo groups and the corresponding 95% confidence intervals will be calculated. Missing values for typing endpoints will be imputed using the default non-response method.

8.4. 3 Safety Analysis

Safety analyses were performed in the Safety Set.

Safety Analysis

The number of subjects with each AE was summarized by Medical Dictionary for Regulatory Activities (MedDRA) system organ class, MedDRA preferred term, and adverse event grade. The number and percentage of subjects with each category of adverse events (including causality, severity, SAE, etc.) will be summarized, and the events within each category will be further summarized by MedDRA system organ class and preferred term.

Safety indicators include: laboratory tests, ECG, vital signs, immunogenicity indicators, hypoglycemic events and injection site reactions.

Laboratory Tests

Measured and changed values of hematology, blood biochemistry, blood lipid, coagulation function, urinalysis, myocardial enzyme spectrum, blood amylase and lipase, thyroid function, serum calcitonin and fasting plasma glucose (tested by central laboratory) before and after treatment were described using mean ± standard deviation, maximum, minimum and median, and normal and abnormal changes before and after treatment were described using cross classification table.

Urinalysis: cross classification table will be used to describe the changes of normal and abnormal before and after treatment.

Describe the proportion of "abnormal and clinically significant" among subjects with abnormal changes, where the abnormality is clinically significant or not as judged by the investigator.

Other

Details of subjects who died were listed.

Describe ECG measurements and changes. The changes between normal and abnormal before and after treatment will be described using cross-categorical tables. Descriptive statistics will be provided for changes in vital signs of subjects.

8.4. 4 Immunogenicity

The occurrence of anti-IBI362 antibodies (ADA) and neutralizing antibodies (NAb) in the serum of subjects before and after dosing will be summarized.

8.4. 5 Analysis of Pharmacokinetic and Pharmacodynamic Parameters

Each PK parameter, including but not limited to Tmax, Cmax, product under the concentration-time curve (AUC), volume of distribution (Vd), half-life (T1/2), clearance (CL) and accumulation coefficient (AR), will be summarized separately for each dose group.

Pharmacodynamic parameters, including fasting plasma glucose, fasting glucagon, fasting insulin, fasting C-peptide, endogenous OXM, and GLP-1, were summarized by group at baseline and at each time point after dosing, and changes from baseline at each time point were summarized.

The changes from baseline in fasting body weight, waist-to-hip ratio, BMI, blood pressure, pulse rate and blood lipids before administration and at 4 weeks (D29), 8 weeks (D57), 12 weeks (D85) and 16 weeks (D113) after administration were summarized and analyzed by groups. The change from baseline in HbA1c at pre-dose and at 12 and 16 weeks post-dose (D113) was analyzed in a pooled manner.

Changes from baseline in islet function (HOMA model) at each time point after treatment were summarized by group.

Changes from baseline in serum uric acid and alanine aminotransferase levels at each

time point after treatment were summarized by group.

8.4. 6 Exploratory Analyses

Changes from baseline in lean body mass (lean mass = body mass-fat mass) were summarized for total body fat content, waist fat content, hip fat content, and waist-to-hip fat ratio measured by dual-energy X-ray absorptiometry (DEXA) and for intra-abdominal fat area (VFA), subcutaneous fat area (SFA), and total abdominal fat area (TFA) measured by MRI after 12 and 16 weeks of dosing.

8.5 **Comparison of multiplicity**

Not applicable.

8.6 Control of bias

8.6.1 Randomization and blinding

The double-blind treatment phase was divided into five cohorts, Cohort 1 (n=12), Cohort 2 (n=12), Cohort 3 (n=12), Cohort 4 (n=12), and Cohort 5 (n=12), and subjects in each cohort were randomized in a 2: 1 ratio to IBI362 (n=8) or placebo (n=4).

Subject randomization will be completed by the IWRS. Successfully randomized subjects will be given a randomization number and will receive drug treatment according to the drug number assigned by the system. Subject and medication blinding was completed by the unblinded statistician and transmitted to the unblinded randomization administrator for preparation and management of the IWRS blinding.

A central randomization method was used, with competing enrollments from each center. A central randomization system will be used for the central randomization procedure. If the subject completes all screening assessments of the study and meets the enrollment criteria, he/she will receive a randomization number generated by IWRS, which will connect the subject to the assigned treatment group and can be assigned to dispense the investigational product according to the amount required. The randomization number of a randomized subject will be retained regardless of withdrawal from the study for any reason. IWRS personnel will only write the randomization table and will not be involved in any specific trial operations. Dispensed study medication must be started within 48 hours after randomization.

8.6.2 Assessment of Blinding Maintenance

The study remained blinded to the subjects and all investigators, monitors, sponsor personnel and representatives involved in the treatment or clinical evaluation of the subjects, except for other personnel from the sponsor who remained unblinded.

8.6.3 Unblinding and Emergency Unblinding

Unblinding of all subjects must be performed after the database is locked, and the statistician shall apply to the sponsor and the principal investigator, and unblinding shall

be performed after the consent of the three parties.

In the clinical study, the investigator may unblind the subject in case of emergency medical events due to the safety of the subject when the investigator needs to know the investigational drug used by the subject. Emergency unblinding must be performed in the system by authorized personnel designated by the site as per procedure. Before unblinding of the investigational drug, relevant personnel of the sponsor should be notified. After obtaining the approval from the principal investigator and the sponsor, the subject should be entered into the IWRS system for emergency unblinding so as to obtain the specific grouping information of the subjects. The investigator should record the time, location and reason for unblinding (the grouping information after unblinding should not be recorded on the eCRF).

9 Quality Assurance and Quality Control

In accordance with GCP guidelines, the sponsor is responsible for implementing and maintaining quality assurance and quality control systems according to corresponding standard operating procedures to ensure that the conduct of clinical trials and the collection, recording and reporting of data comply with the protocol, GCP and corresponding regulatory requirements.

9.1 Clinical Monitoring

The sponsor or a contract research organization (CRO) authorized by the sponsor will perform clinical monitoring of this study. The Clinical Research Associate (CRA) shall perform monitoring in accordance with the standard operating procedures of the Sponsor or CRO and shall have the same rights and responsibilities as the Sponsor's monitor. The monitor should maintain regular communication with the investigator and the sponsor.

Prior to the start of the study, the monitor will assess the competence of each study site and report the relevant problems of facilities, technical equipment, or medical personnel to the sponsor. During the study, the monitor will be responsible for monitoring whether the investigator has obtained written informed consent from all subjects and whether the data records are correct and complete. At the same time, the monitor will also compare the data entered into the eCRF with the original data and inform the investigator of any errors or omissions. The monitor will also control protocol compliance at the study site, arrange for the supply of study drug, and ensure that the drug is stored under appropriate conditions.

Monitoring visits will be conducted as required by applicable laws and regulations. Beginning with subject enrollment, each site will undergo regular monitoring visits. After each visit to the investigator, the monitor should submit a written report to the sponsor.

9.2 Data Management/Coding

Electronic Data Capture (EDC) system will be used in this study, and study data will be entered into the eCRF by the investigator or authorized study personnel. Prior to site initiation or data entry, the investigator and authorized study personnel will be appropriately trained and appropriate security measures will be taken for the computers and other equipment used.

Data entry into the eCRF should be completed as soon as possible during or after the visit and updated at any time to ensure that it reflects the latest developments of the subjects participating in the study. To avoid differences in the assessment of results by different evaluators, it is recommended that baseline and all subsequent efficacy and safety assessments for the same subject be performed by the same person. The investigator was required to review the data to ensure the accuracy and correctness of all data entered into the eCRF. If certain assessments are not performed during the course of the study, or certain information is not available, not applicable, or unknown, the investigator should record it in the eCRF. The investigator should electronically sign the data after verification.

The Clinical Research Associate (CRA) will review the eCRFs against the source documents and assess their completeness and consistency, and the CRA will compare the eCRFs with the source documents to ensure the consistency of key data. All data entries, corrections, and modifications will be the responsibility of the Investigator or his/her designee. The data in the eCRF were submitted to the data server and any changes to the data were recorded in the audit trail, i.e. The reason for the change, operator name, time and date of the modification were recorded. The roles and permissions of the site personnel responsible for data entry will be pre-determined. If there are data queries, CRA or data management personnel will issue the queries in EDC, and the site staff will be responsible for answering the queries. The EDC system will record the audit trail of queries, including the investigator's name, time, and date.

Unless otherwise specified, the eCRF will only be used as a form for data collection and will not be used as source data. Source documents are all records used by the investigator or hospital, related to the subject, and capable of proving the existence of the subject, the inclusion/exclusion criteria and his/her participation in this study, including laboratory records, ECG results, pharmacy dispensing records, subject folders, etc.

The investigator is responsible for maintaining all source documents and for monitoring them by the CRA at each visit. In addition, the investigator was required to submit a completed eCRF for each enrolled subject, regardless of the duration of the enrolled subject's participation in the study. All supporting documents (e.g., laboratory or hospital records) submitted with the eCRF should be carefully verified for the protocol number and subject number, and all personal privacy information (including subject name)

should be deleted or illegible to protect subject privacy. The investigator certifies by electronically signing the record that he/she has reviewed the record and vouch for the accuracy of the data in the record. The electronic signature will be completed using the user ID and password of the investigator. The date and time of the signature will be automatically attached by the system. The investigator may not share the user ID and password with other personnel. Changes to data in the eCRF should be made according to the workflow defined in the EDC system. All changes and reasons for changes will be documented in the audit trail.

Adverse events, concomitant diseases/medical history will be coded. The coding dictionary will be described in the Clinical Study Report (CSR).

9.3 Quality Assurance Audit

Quality assurance audits of the study site, study database, and associated study documents may be conducted by the Sponsor or an authorized representative of the Sponsor during the course of the study, and inspections of the study site, study database, and associated study documents may be conducted at the discretion of the appropriate regulatory authorities. When notified of an inspection by a regulatory authority, the investigator was to notify the sponsor immediately.

Site audits were conducted by the sponsor's Quality Assurance Unit. Audits included: drug supplies, required trial documents, records of the informed consent process, and consistency of the case report forms with source documents. Audit content and scope may also be added as appropriate. After reasonable notification, the investigator should allow auditors entrusted by the sponsor to conduct trial-related audits and inspections by regulatory authorities. The main purpose of the audit or inspection is to verify that the rights or health of the subjects participating in the trial are protected, that the informed consent is signed and the trial process is properly conducted, and that all data related to the evaluation of the study drug are handled and reported in accordance with the pre-planned arrangement, protocol, facilities, ethical Standard Operating Procedure, GCP and applicable regulatory requirements. The investigator should have direct access to all trial documents, original records and raw data.

10 Ethics

10.1 Ethics Committee

The sponsor or its authorized representative of the sponsor will prepare relevant documents to be submitted to the Ethics Committee (EC) of the study site, including the trial protocol, informed consent form, investigator's brochure, subject recruitment materials or advertisements and other documents required by laws and regulations, and submit them to the corresponding EC for review and approval. Written approval from the EC must be obtained and provided to the Sponsor prior to initiation of the study. The EC approval letter must clearly describe the name, number and version number of the study

protocol and the version number of other documents (such as informed consent form) and approval date. The Investigator was required to notify the Sponsor of the EC's written comments on the delay, suspension, and re-approval.

The site must comply with the requirements of the site's EC. It may include protocol amendments, ICF amendments, subject recruitment materials amendments to be submitted to EC for review and approval, local safety reporting requirements, periodic reports and updates according to EC regulations, and final report submission. All of the above documents and EC approvals must be provided to the Sponsor or its designee.

10.2 Ethical Conduct in the Study

The study process and informed consent shall comply with the Declaration of Helsinki, relevant GCP requirements and relevant laws and regulations of China concerning drug and data protection.

GCP provides ethical, scientific and global quality standards for the design, conduct, recording, and reporting of clinical studies involving human subjects. This study will be conducted in accordance with GCP and relevant national regulations and in accordance with the relevant ethical principles in the Declaration of Helsinki to protect the rights, safety and well-being of the subjects.

The investigator is required to comply with the procedures specified in this trial protocol and shall not make changes without the permission of the sponsor. Any protocol deviations will be reported to the EC, Sponsor or Regulatory Authorities.

10.3 Subject Information and Informed Consent

Prior to any study procedures, the possible risks and benefits of the study will be explained to potential subjects using an informed consent form (ICF) that will be easily understood. The ICF statement should specify that the informed consent is voluntary and the possible risks and benefits of participating in the study should be specified, and the subject may withdraw from the study at any time. The investigator can only enroll a subject after fully explaining the details of the study, satisfactorily answering the subject's questions and giving sufficient time for consideration, and obtaining the written consent of the subject or his/her legal representative. All signed informed consent forms must be in the investigator's file or in the subject's folder.

The investigator is responsible for explaining the content of the informed consent to the subject and obtaining the informed consent form signed and dated by the subject or his/her legally acceptable representative prior to the start of the study. After signing, the investigator should send the subject a copy of the signed informed consent form. The investigator should record the informed consent process in the trial source documents.

The initial informed consent form, any subsequent amendments to the written informed consent form, and any written information provided to subjects should be

subject to IRB/IEC opinion prior to use. If new information becomes available that may be relevant to the subject's willingness to continue participation in the trial, the subject or his/her legally acceptable representative should be informed in a timely manner. Communication of this information will be provided and documented via a revised informed consent form or an addendum to the original informed consent form (obtaining the subject's dated signature or the subject's legally acceptable representative's dated signature).

10.4 Data Protection

Information on data protection and privacy will be included in the ICF (or, in some cases, along with the use of separate documents).

Precautions were taken to ensure the confidentiality of documents and to prevent identification of subjects. However, under special circumstances, some individuals may see genetic data and personal identification codes for a subject. For example, in the event of a medical emergency, the sponsor, its representative physician, or investigator will be aware of the subject identification code and have access to the subject's genetic data. In addition, access to relevant documents is required by the relevant regulatory authorities.

10.5 **Protocol Violation**

A protocol violation is defined as any non-compliance with the clinical study protocol, International Conference on Harmonisation Good Clinical Practice (ICH GCP), or Manual of Operations (MOP) requirements. Non-compliance may come from the subject, investigator, or study site staff. In response to the violation, corrective action shall be taken and completed in a timely manner.

11 Study Management

11.1 Data Handling and Record Retention

The documents in the clinical trial (protocol and protocol amendment, completed eCRF, signed ICF, etc.) should be kept and managed in accordance with the requirements of GCP. The site should retain these documents for 5 years after the end of the study.

Study documents should be properly retained for future access or data traceability. Safety and environmental risks should be considered when preserving documents.

No study documents will be destroyed without the written permission of the Sponsor and the Investigator. Only after notifying and obtaining written consent from the Sponsor, the Investigator/study site may transfer the study documents to other parties who comply with the document retention requirements or to other locations where they meet the requirements.

11.2 Access to Raw Data/Documents

The Investigator agrees that the Sponsor, CRO and relevant authorized regulatory

authorities have direct access to all study-related documents, including the subject's medical records.

11.3 Protocol Amendment

Any amendments to the protocol that may be appropriate during the course of the study will be communicated and agreed upon by the Sponsor and the Investigator. The sponsor should ensure that protocol amendments are submitted to regulatory authorities in a timely manner.

All amendments to the protocol will be retained as protocol supplements. Any amendment to the protocol should be submitted to the Ethics Committee for approval or filing according to the provisions of the Ethics Committee. If required, it should also be submitted to regulatory authorities for approval and, if required, approved by the EC and regulatory authorities before implementation (except for changes to the protocol to eliminate an immediate hazard to trial subjects).

11.4 Investigator Responsibilities

The investigator will conduct this study in accordance with the protocol, ethical principles in the Declaration of Helsinki, China GCP and relevant regulatory requirements.

The detailed responsibilities of the relevant investigators are listed in the Chinese GCP (2020 No.57), Chapter 5.

11.5 Publication Policy

All data generated in this study are confidential information of the Sponsor. The Sponsor has the right to publish the results of the study. Information on the publishing policies of the sponsor and investigators will be described in the clinical trial agreement.

All information related to this trial (not limited to the following documents: protocol, Investigator's Brochure) must be strictly confidential. The investigator must be aware that the scientific or medical information derived from this trial may have commercial value to the sponsor. The investigator shall keep the information and data related to this trial confidential. If the information related to this trial or the conclusions drawn from the trial are to be published publicly, the investigator shall negotiate with the sponsor in advance and obtain the written consent of the sponsor. In order to protect their own rights and interests, the sponsor may require the investigator not to publish information related to the trial before the investigational product is approved for marketing.

The sponsor has the right to publish or publish information or data related to this trial or to report it to the drug regulatory authorities. If the sponsor needs to include the name of the investigator in the publication, publication, or advertisement, the investigator's consent should be obtained.

11.6 Finance and Insurance

The sponsor will purchase insurance for subjects participating in this study in accordance with local regulations and minimum requirements. The terms of the insurance will be kept in the study binder.

12 Protocol Amendment History

Version	Date	Description of Change
V1.0	02/28/2020	N/A, this is a new protocol
V1.1	24/04/2020	See Protocol Amendment Record for details
V2.0	11/13/2020	See Protocol Amendment Record for details
V2.1	2020/12/14	See Protocol Amendment Record for details

13 References

[1]. Pocai A. Action and therapeutic potential of oxyntomodulin. Mol Metab. 2013; 3 (3): 241-251.

[2]. Tan TM, Field BC, McCullough KA, Troke RC, Chambers ES, Salem V, Gonzalez Maffe J, Baynes KC, De Silva A, Viardot A, Alsafi A, Frost GS, Ghatei MA, Bloom SR. Coadministration of glucagon-like peptide-1 during glucagon infusion in humans results in increased energy extension and amelioration of hyperglycemia. Diabetes. 2013; 62 (4): 1131-1138.

[3]. Campbell JE, Drucker DJ. Nature ReviewsEndocrinology, 2015, 11 (6): 329-338.

[4]. Obesity Group, Chinese Society of Endocrinology, Chinese Medical Association. Expert consensus on prevention and treatment of adult obesity in China. Chinese Journal of Endocrinology and Metabolism, 2011, 27 (9): 711-717. Tab, 2008, 93: 4576-4599.

[5]. Marie Ng 1, Tom Fleming, et al. Global, Regional, and National Prevalence of Overweight and Obesity in Children and Adults During 1980-2013: A Systematic Analysis for the Global Burden of Disease Study 2013. Lancet, 384 (9945), 766-81).

[6]. Https://www.cdc.gov/obesity/data/index.html

[7]. Snow V, Barry P, Fitterman N, et al. Pharmacological and surgical management of obesityin primary care: a clinical practice guideline from the American College of Physicians [J]. Ann Intern Med, 2005, 142: 525.

[8]. George A Bray, et al. Management of Obesity. Lancet, 387 (10031), 1947-56.

[9]. Knowler WC, Barrett-Connor E, Fowler SE, Hamman RF, Lachin JM, Walker EA et al. Reduction in the incidence of type 2 diabetes with lifestyle intervention or metformin. N Engl J Med 2002; 346 (6): 393-403.

[10]. Dattilo AM, Kris-Etherton PM. Effects of weight reduction on blood lipids and lipoproteins: a meta-analysis. Am J Clin Nutr 1992; 56 (2): 320-328.

[11]. Pocai A. Unraveling Oxyntomodulin, GLP1's enigmatic brother. J Endocrinol. 2012; 15: 335-346.

[12]. Day JW, Ottaway N, Patterson JT, et al. A new glucagon and GLP-1co-agonist eliminates obesity in rodentis.Nat Chem Biol. 2009; 5: 749-757.

[13]. Kosinski JR, Huber J, Carrington PE, et al. The glucagon receptor is evolved in mediating the body weight-lowering effects of oxyntomodulin. Obesity. 2012; 20: 1566-1571.

[14]. Lao J, Hansen BC, DiMarchi R, et al. Effect of GLP1R/GCGR dual agonist in monkeys. Diabetes. 2013; 62 (suppl 1): A257.

[15]. Ralf Elvert, Andreas W. Herling. Running on mixed fuel-dual agonistic approach of GLP-1 and GCG receptors leads to beneficial impact on body weight and blood glucose control: A comparative study between mice and non-human primate. Diabetes Obes Metab. 2018; 20: 1836-1851.

[16]. Jorgensen R, Kubale V, Vrecl M, Schwartz TW & Elling CE 2007 Oxyntomodulin differential affectives glucagon-like peptide-1 receptor β-arrestin receptor and signaling through Ga (s). Journal of Pharmacology and Experimental Therapeutics 322 148-154.

[17]. NIA Adverse Event and Serious Adverse Event Guidelines (2017).

14 Appendix

Appendix 1: Pancreatic Enzyme Monitoring and Management Procedures for Patients without Symptoms of Pancreatitis 1, 2

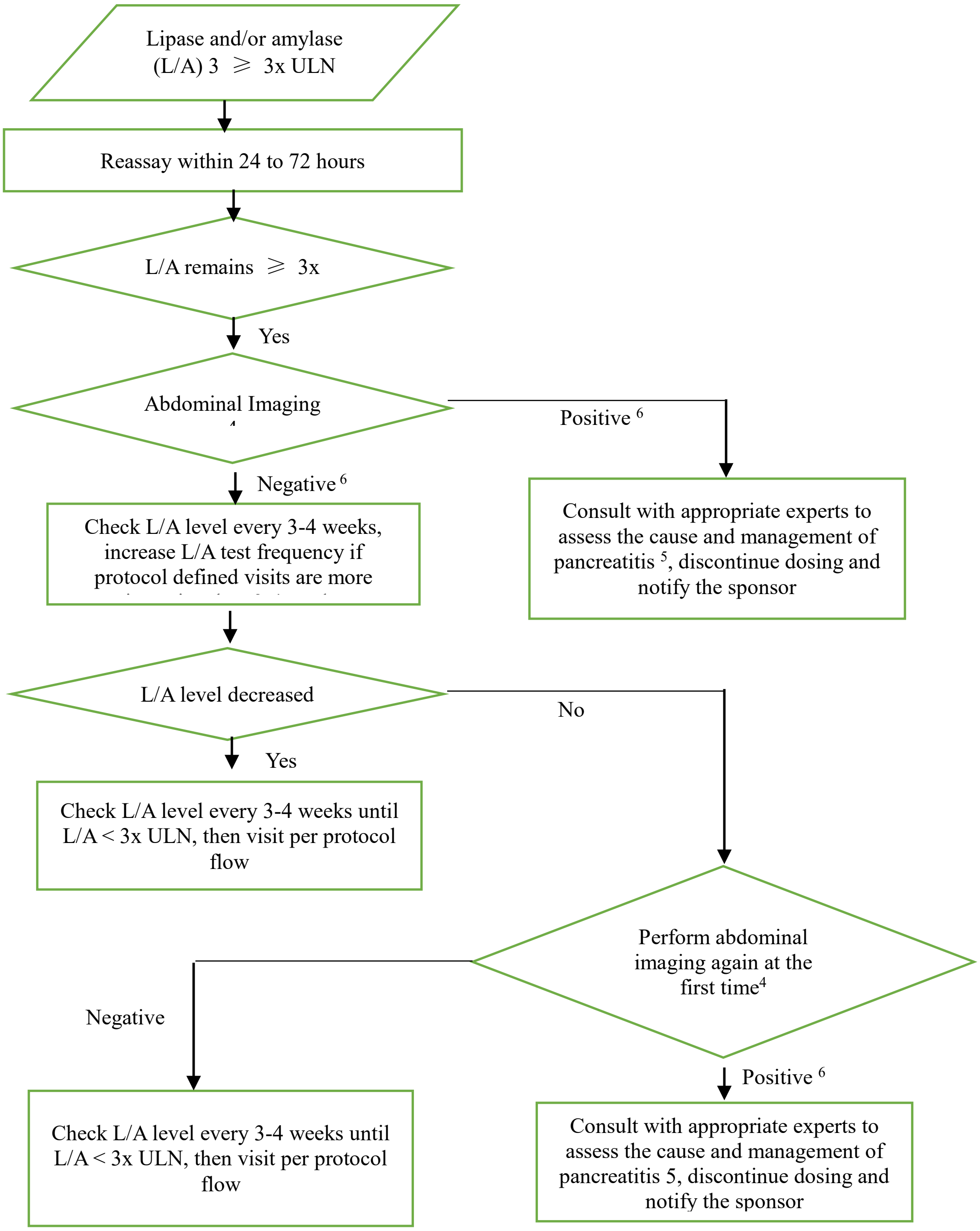

1. Symptomatic refers mainly to abdominal pain associated with pancreatitis, and severe nausea, vomiting, and other symptoms can also be considered as related symptoms by the investigator.
2. If at any time the investigator believes that a subject has symptoms of acute pancreatitis, regardless of whether based on L/A test results, the investigator should consult a specialist for evaluation, management, assessment of the cause of pancreatitis, discontinuation of dosing and notification of the sponsor.
3. Either or both of serum lipase and serum amylase can be used as the evaluation criteria.
4. The optimal time for abdominal imaging is when elevated pancreatic enzymes are just found. If judged safe by the investigator or imaging physician, it is best to use enhanced

Appendix 2: CKD-EPI formula (eGFR estimation formula)

Estimated by CKD-EPI formula: eGFR = a × [(serum creatinine (μ mol/L)/b)] c × (0.993) age;

Sex	A Value	B-value	C-value	
			Serum creatinine ≤ 0.7mg/dl	Serum creatinine > 0.7 mg/dl
Female	144	0.7	-0.329	-1.209
Male	141	0.9	-0.411	-1.209

Appendix 3: QTcF Calculation Formula

QTc Fridericia formula: QTcF = QT/(RR ^ 0.33)

Appendix 4: Dyslipidemia Reference Standards

Refer to Guidelines for Prevention and Treatment of Dyslipidemia in Chinese Adults (2016 Revision)

Appendix 5: Oral Glucose Tolerance Test (OGTT) Methods

1. Beginning at 7-9 a.m., subjects were orally administered 75 g of anhydrous glucose dissolved in 300 mL of water after fasting (8-10 h), or 82.5 g if 1 molecule of water was used. Children are given 1.75 g per kilogram of body weight, not to exceed 75 g. Sugar water was taken within 5 minutes.
2. Blood samples were taken from forearm before and 2 hours after taking glucose from the first mouth of glucose.
3. During the course of the study, the subjects did not drink tea or coffee, did not smoke, did not do strenuous exercise, but were not absolutely bed-ridden.
4. Blood samples should be submitted as soon as possible.
5. The daily carbohydrate intake shall not be less than 150 g within 3 days prior to the test.
6. The drugs that may affect OGTT, such as contraceptives, diuretics or phenytoin sodium, were stopped for 3 ~ 7 days before the test.

Appendix 6: AE Reporting Process Related to Dose Escalation Discontinuation

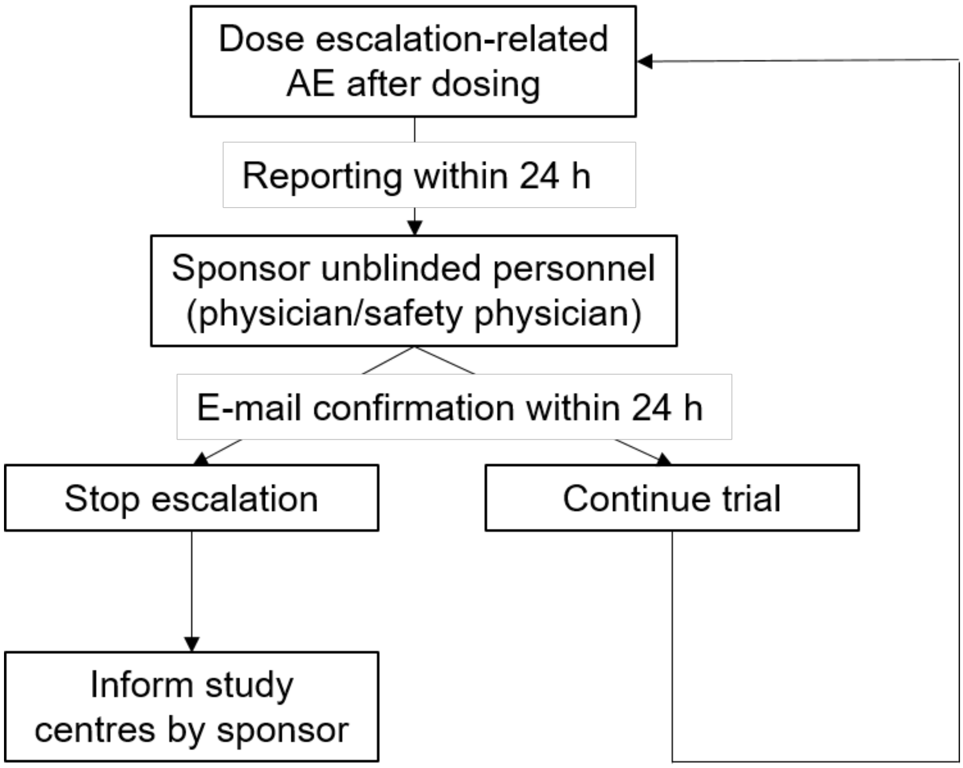

Supplement: Supplementary file 1 [file mmc1.pdf]
